# A large dataset of brain imaging linked to health systems data: curation and access to a whole system national cohort from NHS Scotland

**DOI:** 10.1093/gigascience/giag072

**Published:** 2026-06-09

**Authors:** Michael P J Camilleri, Dorian Gouzou, Salim Al-Wasity, Muthu R K Mookiah, María Valdes Hernandez, Bea Alex, Sotirios A Tsaftaris, Andrew Brooks, Ruairidh MacLeod, Honghan Wu, Brenda Bauer, Claire Grover, Parminder Reel, Susan Krueger, Richard Tobin, J Douglas Steele, Grant Mair, Joanna Wardlaw, Alexander Doney, Emanuele Trucco, William Whiteley

**Affiliations:** Computing, School of Science and Engineering, University of Dundee, Dundee, DD1 4HN, UK; School of Engineering, University of Edinburgh, Edinburgh, EH9 3JL, UK; Institute for Neuroscience and Cardiovascular Research, School of Medicine, University of Edinburgh, EH16 4TJ, UK; School of Medicine, Ninewells NHS and University Hospital, Dundee, DD2 1GZ, UK; School of Medicine, Ninewells NHS and University Hospital, Dundee, DD2 1GZ, UK; Institute for Neuroscience and Cardiovascular Research, School of Medicine, University of Edinburgh, EH16 4TJ, UK; School of Informatics, University of Edinburgh, Edinburgh, EH8 9AB, UK; School of Engineering, University of Edinburgh, Edinburgh, EH9 3JL, UK; Edinburgh Parallel Computing Centre, University of Edinburgh, Edinburgh, EH8 9BT, UK; Edinburgh Parallel Computing Centre, University of Edinburgh, Edinburgh, EH8 9BT, UK; Usher Institute, School of Medicine, University of Edinburgh, Edinburgh, EH16 4UX, UK; School of Health and Wellbeing, University of Glasgow, Glasgow, G11 6EW, UK; Institute for Neuroscience and Cardiovascular Research, School of Medicine, University of Edinburgh, EH16 4TJ, UK; School of Informatics, University of Edinburgh, Edinburgh, EH8 9AB, UK; Health Informatics Centre, School of Medicine, University of Dundee, Dundee, DD2 1FD, UK; Health Informatics Centre, School of Medicine, University of Dundee, Dundee, DD2 1FD, UK; School of Informatics, University of Edinburgh, Edinburgh, EH8 9AB, UK; School of Medicine, Ninewells NHS and University Hospital, Dundee, DD2 1GZ, UK; Institute for Neuroscience and Cardiovascular Research, School of Medicine, University of Edinburgh, EH16 4TJ, UK; Institute for Neuroscience and Cardiovascular Research, School of Medicine, University of Edinburgh, EH16 4TJ, UK; UK Dementia Research Institute Centre at the University of Edinburgh, EH16 4SB, UK; Cardiovascular Research, School of Medicine, University of Dundee, Dundee, DD1 9SY, UK; Computing, School of Science and Engineering, University of Dundee, Dundee, DD1 4HN, UK; Institute for Neuroscience and Cardiovascular Research, School of Medicine, University of Edinburgh, EH16 4TJ, UK; Usher Institute, School of Medicine, University of Edinburgh, Edinburgh, EH16 4UX, UK; Health Data Research UK, London, NW1 2BE, UK

**Keywords:** MRI, CT, neuroimaging, artificial intelligence, healthcare data

## Abstract

**Background:**

We present the design and implementation of a data curation framework to generate a large-scale clinical brain imaging dataset suitable for artificial intelligence (AI) enabled image analysis.

**Findings:**

The dataset is accessible through the Brain Health Data (BHD) initiative, which includes ~417,341 magnetic resonance imaging (MRI) and 846,077 computerized tomography head studies, linked electronic health records, and associated free-text imaging reports from clinical practice between 2010 and 2018 in Scotland, exceeding 185 TB in size. The data curation framework was developed during the SCottish AI in Neuroimaging to Predict Dementia and Neurodegenerative Disease (SCANDAN) study, which used a subset of 41,966 MRI series from the BHD for dementia prediction.

We describe the processing of the BHD metadata and our multilabel classification output. We discuss the strengths of the BHD, including clinical relevance thanks to its unprecedented scale, population-wide representativeness of a national free-at-the-point-of-delivery healthcare, long-term follow-up to neurodegenerative disease, and real-world variability. We describe the challenges and lessons learnt in developing a framework to curate data, including the time needed to obtain permissions, the need for easily accessible, secure, responsive and affordable computational environments, the variability of clinical data, and the challenge of extracting linked clinical data and images at scale.

**Conclusion:**

This resource will be crucial for clinical research, fostering the development of personalized medicine approaches, and fast-tracking the implementation of AI models in clinical workflows. We encourage the use of the BHD data through a streamlined application to the Public Benefit and Privacy Panel for Health and Care via the electronic Data Research and Innovation Service of Public Health Scotland (eDRIS).

## Introduction

Brain imaging plays a crucial role in the diagnosis of neurological disorders. However, clinical imaging services are under great demand, highlighting the need for new tools to improve radiology workflows. These tools should accelerate image assessment, reduce the workload for radiologists, and ultimately improve patient care. Artificial intelligence (AI) methods show promise for faster diagnosis, for example, in acute ischaemic stroke [1], and similar improvements are possible in head  injury, neurodegeneration, dementia, and brain cancers [2, 3]. To develop and test AI models that are clinically relevant, researchers need access to large datasets of clinically acquired images, and secure, ethical data provision. Such large datasets, however, are complex to process due to the computational bottlenecks of several open-source software suites [4]. Hence, secure environments should also provide sufficient computing resources.

A survey conducted between December 2024 and February 2025 across UK secure data environments revealed the lack of brain imaging resources with nationwide coverage. For instance, the Diagnostic Imaging Dataset curated by NHS England includes patient-level metadata on the 501 million diagnostic imaging procedures performed in NHS England since April 2012, but it lacks imaging data and associated reports [5].

The preparation of large repositories of routinely collected imaging data is challenging, particularly in privacy-protecting secure data environments. Despite adherence to Digital Imaging and Communications in Medicine (DICOM) [6] standards, real-world medical imaging datasets vary significantly in quality, format, and acquisition protocols, which makes standardization across different imaging sources necessary. Determining imaging sequences (e.g., T1- or T2-weighted magnetic resonance imaging [MRI]) is essential for analysis but can be difficult in practice. DICOM metadata tags provide rapid but sometimes unreliable classification, while image-based classification is more accurate but computationally demanding, and not free from uncertainty [7, 8]. Natural language processing (NLP) of radiology reports can facilitate sequence identification and filter out scans with artefacts or missing structures. However, automated image quality assessment is paramount. Pre-processing and data retrieval are easier with automation of pipelines and integration of structured clinical records.

Compliance with governance frameworks is important to access large-scale unconsented clinical imaging datasets within safe havens, and needs ethical approval, data governance approval, and compliance with privacy regulations, all with costs. These administrative barriers, although necessary, can significantly delay or completely deter research.

To address these challenges, we developed the Brain Health Data (BHD) framework, which unifies all clinical brain imaging data acquired in Scotland with linked clinical information, to facilitate access to data through the Electronic Data Research and Innovation Service (eDRIS) of Public Health Scotland (PHS) [9]. The data within the BHD framework include ~417,341 MRI and 846,077 computerized tomography (CT) head studies, linked electronic health records (EHRs), and free text radiology reports that were collected between 2010 and 2018. It offers clinical relevance with its unprecedented scale, population-wide representativeness of healthcare, long-term follow-up to neurodegenerative disease, and real-world variability. To curate this large-scale clinical brain imaging dataset in a format suitable for AI analysis, we designed and implemented a data processing pipeline within the SCottish AI in Neuroimaging to Predict Dementia and Neurodegenerative Disease (SCANDAN) study, which aimed to develop AI algorithms for reliable dementia risk estimation from routine brain imaging and clinical records. This paper describes SCANDAN’s methods and outputs, highlights key lessons learned from working within Scotland’s data governance frameworks, and describes how to access the data through the BHD.

## Methods

### Permissions and research governance

The SCANDAN study obtained multicentre research ethics permission from the North of Scotland Research Ethics Committee (23/NS/0017). Permission to access the data for SCANDAN and the pilot phase for the BHD was provided by the NHS Scotland Public Benefit and Privacy Panel for Health and Social Care (PBPP), which scrutinises applications for access to NHS Scotland health data for non-direct care (PBPP applications 2223-0200 and 2223-0005, respectively) [10]. During the application process and throughout the study, the SCANDAN team engaged with several Scottish public and patient groups.

### Computing environment

The Scottish National Safe Haven (NSH), commissioned by PHS, where all processing is done, is hosted in Edinburgh Parallel Computing Centre’s (EPCC) Trusted Research Environment (TRE), a secure infrastructure which currently hosts 12 Safe Havens. Each Safe Haven is operated under the “Five Safes” framework [11] and the Scottish Government Charter for Safe Havens [12]. Researchers access a secure data sharing and analysis environment with a virtual desktop, under the terms and conditions prescribed by the data controllers. Standard software packages such as R and Python are available in the NSH; additional software packages can be installed from repositories such as the comprehensive R archive network and the Python package index. Safe Havens have access to large shared-memory, high-performance computer clusters, including one with graphical processing unit (GPU) accelerators for large-scale analysis. For example, SCANDAN was provided a virtual environment with a GPU (NVIDIA A100 40GB), large storage (several TB), and RAM (100GB). All EPCC Safe Haven Services are operated at EPCC’s Advanced Computing Facility, located in Edinburgh, Scotland. The EPCC TRE is accredited by ISO27001 [13] for information security practices and self-certified under Cyber Essentials and NHS England’s Data Security and Protection Toolkit (DSPT). In addition, the NSH is accredited under the Digital Economy Act 2017 by the UK Statistics Authority, and all Safe Havens in the TRE are operated to the same standard.

### Data sources

PHS’ eDRIS provided brain CT and MRI head studies in adults performed in Scotland between 2010 and 2018 from the Scottish Medical Imaging service [14]. Study refers here to a complete imaging session, encompassing all images obtained during a single scanning session. Each scan contains 3 hierarchical levels: study, series, and images. Within each study, there are one or more series that group together images acquired using the same imaging technique and settings. Each series is, in turn, composed of multiple single 2-dimensional images or “slices.” A patient may have had multiple independent studies. Additionally, imaging reports are associated with studies and contain text about the imaging process and clinical interpretation of images. eDRIS and EPCC linked studies to patients deterministically with pseudonymized identifiers based on the Community Health Index (CHI) number, which is the unique patient identifier used across NHS Scotland. We linked them with outpatient records (SMR00), hospital admission records (SMR01), dementia records from mental health hospitalizations (SMR04), cancer registry (SMR06), community dispensed prescriptions from Prescribing Information System, death records (National Records of Scotland [NRS]), and demographics (birth year, sex, deprivation index) since the year 2000. All the data were processed and stored within the NSH.

### Data description

**Figure 1:**
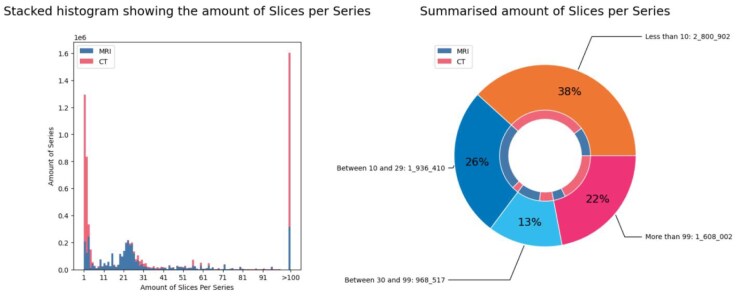
The distribution of slices per series within the data available through the BHD, for both MRI and CT. The stacked histogram shows the 2D MRI has a normal distribution of scans centred around 22, while the 3D MRI has over 100 slices. The CT has mostly more than slices. Both modalities have a large amount of localizer scans with under 10 slices.

The data on 830,884 patients were provided to SCANDAN and are available through the BHD. It includes 417,341 MRI studies, 846,077 CT studies, and 1.8 million radiological reports. The studies contain 3.37 million MRI series and 3.15 million CT series. Figure 1 shows the distribution of slices per series for both CT and MRI for which the metadata were available at the start of the project. There were 356 million events from EHR, divided between the outpatient emergency and inpatient records (38 million), death records (327,000), prescription records (312 million), and accident and emergency records (4.5 million). For the 410 million DICOM slices available (130.3 million MRI and 279.7 million CT), DICOM metadata were limited by the governance approval, with each tag being subject to approval. Consequently, the accepted metadata were provided separately in CSV format. Table 1 describes key metadata on dataset heterogeneity.

**Table 1:** Number of unique values and percentage of empty fields for key brain scans DICOM tags.

		% series with empty field		
DICOM tag	Number MRI	CT	MRI	CT
“Protocol Name” (0018,1030)	185,591	4,029	1.59%	12.27%
“Series Description” (0008,103E)	56,624	17,826	1.73%	<1%
“Performed Procedure Step Description” (0040,0254)	3,962	3,125	3.69%	79.13%
“Body Part Examined” (0018,0015)	1,821	243	53.88%	41.13%
“Institution Name” (0008,0080)	722	611	1.32%	<1%

Tags like “Institution Name” or “Body Part Examined” contains different values with the same meaning (e.g., “Head&Neck,” “head and neck,” “HEAD_NECK”). These were counted as different occurrences.

### SCANDAN project

**Figure 2:**
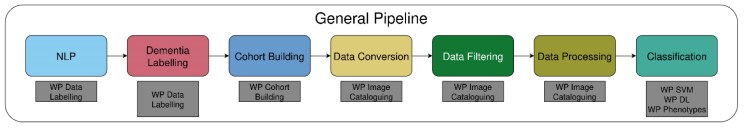
Work packages in the SCANDAN project: Data labelling, cohort building, image cataloguing, and processing for classification into being indicative of having dementia or not using deep learning (DL), support vector machine (SVM), and from the analysis of extracted imaging phenotypes.

The SCANDAN sequential work packages (WP) are illustrated in Fig. 2. The NLP WP identified MRI sequences, CT type, and brain pathologies from the radiological reports. The dementia labelling WP phenotyped dementia with EHRs. The cohort building WP selected a subset of MRI studies to carry out dementia classification. The image cataloguing WP labelled DICOM series with body part imaged, sequence, and presence of contrast and then filtered out non-desired scans based on the label. Images were then processed for AI analysis.

### SCANDAN: NLP of brain imaging reports

We applied a clinical NLP tool, the Edinburgh Information Extraction for Radiology (EdIE-R) [15, 16], which was originally developed and validated for radiology reports of brain imaging in the Edinburgh Stroke Study and NHS Tayside [17]. EdIE-R processes radiology reports through a pipeline that identifies entities, detects negation, extracts relationships, and assigns document-level labels to identify phenotypes. The tool was later adapted and validated for use with data from other areas in Scotland provided by Generation Scotland. EdIE-R can extract 24 distinct phenotypes, including different stroke types (ischaemic, haemorrhagic, and underspecified, with time and location details), brain tumours (meningiomas, gliomas, metastases, or underspecified), small vessel disease, microbleeds, atrophy, and other abnormalities. Additionally, it marked up MRI sequence types (T1, T2, and Fluid-Attenuated Inversion Recovery [FLAIR]).

To improve data selection, EdIE-R was enhanced to identify scans of non-head and non-brain body parts, and flag them for exclusion. We improved the tool’s ability to identify where distinct sections begin and end within reports, such as the boundary between the clinical history preamble and the main report body, enabling us to extract phenotype mentions exclusively from the relevant report text.

EdIE-R contains several processing components. After pre-processing and linguistic analysis (e.g., tokenization, sentence detection, lemmatization, and part-of-speech tagging) of the text in the input radiology report, EdIE-R performs named entity recognition, negation detection and relation extraction before conducting document-level classification of the 24 phenotypes. The output is the radiology report and its accompanying metadata as well as the information identified by EdIE-R represented in XML format, which was then converted to CSV for follow-on analysis. It is keyed by the study identifier and does not contain information about a specific series.

The refined EdIE-R pipeline [16] was applied to all radiology reports in the SCANDAN project, producing structured outputs to guide data selection for image analysis. By processing radiology reports within the Scottish NSH, the tool allows exclusion of scans (e.g., those showing tumours or non-brain regions) and serves to validate outputs from imaging type classification and phenotype extraction.

Structured report DICOM contains TextValue elements of various kinds. Some are clinical reports; others contain non-clinical information. We aimed to select the best clinical report in each study for NLP processing. Not all study directories contained structured reports, and some contained more than one. For the latter, the process of choosing a report was as follows. From inspection of examples, it appeared that DICOM files containing real reports normally contained exactly one TextValue element. If a study contained one or more such DICOM files, we used the largest of those. In the cases where no report contained exactly one TextValue element, we just used the largest DICOM file, and processed the first TextValue element. If this was not a clinical report, it would usually be marked as “empty” or “nocontent” by the NLP pipeline. Some reports were withheld because they were potentially identifiable, which accounts for studies with no report or no real report.

### SCANDAN: phenotyping dementia

We follow the phenotype specification for dementia [18] based on prior studies in Scottish EHRs [19]. Dementia was defined as the presence of relevant ICD-10 codes, and, in addition for the specific case of Alzheimer’s disease (AD), the prescription of AD medications. Each patient interaction with the health system, taken from the EHR, was used, including a single stay in hospital, multiple consecutive stays, a prescription, or a death record. We defined labels for “any dementia” and five dementia subtypes: AD, vascular dementia (VaD), other rare dementias, unspecified dementia, and possible dementia. The subtype was defined as the most frequently occurring dementia phenotype in each person’s electronic record (Table 2). All individuals with a dementia label were categorized as cases, and individuals with no mention of dementia in any record were considered controls.

**Table 2:** International Classification of Diseases 10 (ICD-10) and British National Formulary (BNF) codes to define dementia subtypes.

Subtype	ICD10	BNF
Alzheimer’s disease	F00* G30*	0411000
Vascular dementia	F01*	
Other rare dementias	F02*, G31.0, A81.0	
Unspecified dementia	F03, F05.1	
Possible dementia	F05.0, G31	

The definition of the codes follows the phenotyping employed previously [18]. * indicates a wild-card, meaning that all child-codes in the hierarchy are included.

### SCANDAN: cohort building

We built a matched case-control study cohort with MRI brain images. A matched case-control design was chosen for several reasons. First, most deep learning and other algorithms work best with balanced cases and controls. Second, we had limited computing capacity at the beginning of the project. Third, the rate of image delivery was limited by the need to copy data from a preparation area to a research area, which had limited storage capacity. We selected images with no NLP label of tumour or haemorrhagic stroke in the radiology report from patients who were aged over 40 years at the time of scan and had an associated EHR record. We excluded dementia cases without scans taken more than 1 year before the time of diagnosis. For each individual, the first study chronologically was chosen. Dementia cases were matched to controls based on age at the time of scan (within 1 year of the matched case) and recorded sex from the linked demographic information. Cases with no matched controls were discarded. Age and sex matching was verified by analysing the resulting distributions over the entire cohort. A table was generated containing the identifier of the selected patient, their match, the selected study, demographic information, and the dementia ground truth.

### SCANDAN: data validation and quality control

To generate ground truth labels for an initial evaluation of the automatic labelling process described in the next section, we developed a custom Python-based graphical user interface (GUI) optimized for MRI and CT DICOM files. The GUI allowed users to load DICOM images from a single or nested folder structure. It utilized DICOM header metadata to stack slices according to the acquisition order using the DICOM tag “Instance Number” (0020,0013), in ascending, descending, or interleave format, constructing and saving the 3D volumes for subsequent analysis; filter CT scans with the tag “Modality” (0008,0060) and adjust their intensities, e.g., brain-windowing, using the tag “Rescale Intercept” (0028,1052); determine the orientation of the imaging planes (i.e., axial, sagittal, or coronal) using the tag “Image Orientation (Patient)” (0020,0037) to display mid-axial, mid-coronal, and mid-sagittal views for assessment; and calculate the aspect ratio using the “Slice Thickness” (0018,0050) and “Pixel Spacing” (0028,0030) tags for accurate scaling and visualizing of mid-view slices within the designated display area.

**Figure 3:**
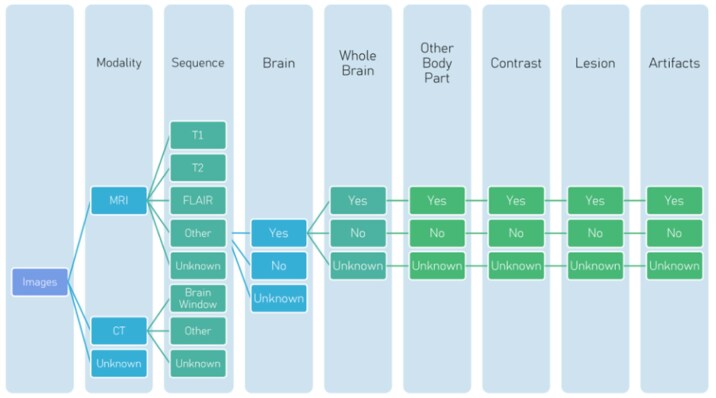
Criteria used by the annotators to label the test imaging set using the GUI developed.

Randomly sampled example series (1,000) were selected prior to the large data delivery without stratification for image review. Among these, 287 were excluded for potential disclosive information. The remaining 390 MRI and 323 CT were annotated by 5 experts (3 clinicians and 2 trained imaging scientists) with modality, sequence, presence of contrast, lesion and artefacts, presence of full brain, and presence of body parts (Fig. 3). The 713 series were evenly divided among the 5 annotators, with a subset of 100 images overlapping for cross-validation. The Generative model of Labels, Abilities, and Difficulties [14] probabilistic framework was used to estimate the true label for each image while accounting for annotator expertise and image difficulty, by using the overlapping 100 annotations as "truth” and correcting the rest of the annotations.

The ground-truth labels were compared with the labels obtained from the automatic labelling. Scan sequence type and contrast presence were further reviewed by a neuroradiologist and an experienced imaging scientist independently for the disagreement with the automatic labels, or for previous “unknown” or “uncertain” annotations. A third round of annotations resolved disagreements between the neuroradiologist and the imaging scientist. The presence of brain, of other body part, and the fullness of the brain were re-annotated by a trained image scientist as an independent re-annotation. After re-annotation, the ground-truth was updated using the most recently agreed version. We report the final comparison of the automatic tools and the manual annotations.

### SCANDAN: identification of MRI scan type and sequence

**Figure 4:**
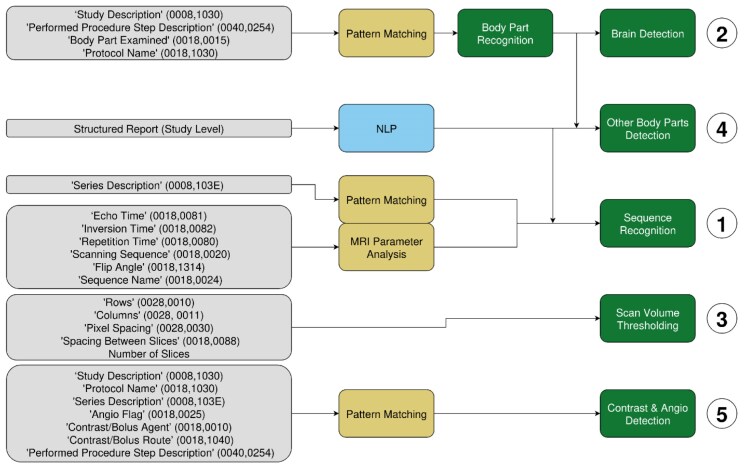
The exclusion process for the SCANDAN cohort. It is based on the automatic labelling , including the pattern matching and the MRI parameter analysis) and the NLP of the radiological report. The steps were performed in the order from 1 to 5, numbered on the right. (1) Non-T1/T2/FLAIR scans are excluded. (2) Scans without a brain are excluded. (3) Scans too small to contain a full brain are excluded. (4) Scans with other body parts (such as the spine) are excluded. (5) Contrast and angio scans are excluded.

DICOM tags were used to produce 5 labels for each image series: imaging sequence, presence of brain, presence of other body part, angiography, and imaging with contrast. We aimed to retain MRI series with sequences T1, T2, and FLAIR that contained a brain and no other body part than the neck, without angiography or contrast, and with a 3D image volume of over 5 litres. The volume was empirically determined on another study using Scottish medical data, compared to the manual annotations, and visually asserted to separate 2 different normal distribution of scans volume [20]. These labels were subsequently combined with the results from the NLP tool, the MRI acquisition parameters, and the computed volume of the image series, to exclude those that did not meet SCANDAN criteria. Figure 4 illustrates the methods and data sources for the labelling and exclusion of images.

To produce these labels, the DICOM tags were parsed with regular expressions (RRID:SCR_028365, biotools:scandan-dicom-labelling) [21]. For example, the expressions /(?i)(?<!pa)t2/(case insensitive and ignoring occurrences starting with “pa”) and/*se2d1/ were associated with the intermediate label “tmp-T2” (T2-weighted), while the expressions /TOF/and/MRA/ were associated with the intermediate label “tmp-MRA” (MR angiography). Then, the final labels were created by grouping all the intermediate labels of a series. For example, for an image series to be labelled “T1,” it had to match the intermediate label “tmp-T1,” and could optionally match the intermediate labels “FLAIR,” “GRE” (gradient echo), and “FAT SAT” (fat saturation), which are not T1-weighted exclusive, but no any other intermediate label.

For sequence identification, the DICOM tag “Series Description” (0008,103E) was used. To identify the body part, we used the tags “Body Part Examined” (0018,0015), “Protocol Name” (0018,1030), “Performed Procedure Step Description” (0040,0254) and “Study Description” (0008,1030). Angiograms were identified with the tags “Angio Flag” (0018, 00), “Study Description” (0008,1030), “Protocol Name” (0018,1030), and “Series Description” (0008,103E). Contrast identification used “Study Description” (0008,1030), “Contrast/Bolus Agent” (0018,0010), “Contrast/Bolus Route” (0018,1040), “Performed Procedure Step Description” (0040,0254), and “Series Description” (0008,103E).

The results from the NLP tool provided additional information for the sequence identification and the presence of other body parts, validating the presence of a sequence within a study. Finally, to complement the identification based on the series description the MRI sequence was also defined with the tags “Echo Time” (0018,0081), “Inversion Time” (0018,0082), “Repetition Time” (0018,0080), “Scanning Sequence” (0018,0020), “Flip Angle” (0018,1314), “Sequence Name” (0018,0024) based on “optimal” value [7, 8] adapted to the data through observation on manually annotated data.

Each regular expression rule was based on prior research carried on in-house clinical studies. They were expanded to ignore conflict and formatting due to the greater distribution of value from the 35 hospitals which the data originates from. The most common occurrence for each DICOM tag was compared to their label using the metadata of the entire cohort, to ensure the rules were not including unwanted samples. Finally, they were refined to agree with the manual annotation during each re-annotation.

## Results

### SCANDAN: output dataset

Taking the first chronological study in each sequence of studies (MR or CT) for each person gave 1.1 million studies, of which 311,000 were MRI and 789,000 CT. Among these, 16,000 MRI and 119,000 CT were associated with a record of dementia. After applying exclusion criteria, as described in Table 3, 10,709 MRI and 57,242 CT dementia cases were age and sex matched with the same number of healthy controls. We eliminated 1,171 MRI and 3,302 CT dementia cases with a text report containing a mention of tumour or haemorrhagic stroke; 70 MRI and 100 CT dementia cases due to the patient being under 40 at the time of scans; and 4,869 MRI and 58,779 CT cases because the scans occurred within 1 year prior to the dementia diagnosis.

**Table 3:** Selection process for the MRI and CT cohorts, showing both the overall and dementia counts, with addition of characteristics.

	MRI		CT	
Criterion	Overall	Dementia	Overall	Dementia
With scan	294,422	16,819	669,539	119,423
+No reported tumour or haemorrhagic stroke	280,549	15,648	640,400	116,121
+Hospitalized electronic health record	279,004	15,648	638,680	116,121
+Diagnosis other than “possible” dementia	275,970	15,648	627,911	116,121
+Age at scan > 40	207,876	15,578	523,855	116,021
+Dementia diagnosis or follow-up > 1 year	190,582	10,709	391,356	57,242

MRI: magnetic resonance imaging of brain; CT: computerized tomography of brain.

Of the 1,481,643 study directories, 449,369 had no structured report, 655,450 study directories contained exactly 1 report, and 376,824 study directories contained more than 1 report. Each selected report was processed using the EdIE-R NLP pipeline. Across the full dataset, the most frequently detected phenotypes were small vessel disease (25.0% of studies) and atrophy (23.2%), reflecting their high prevalence in an ageing clinical population. Ischaemic stroke findings were also common (e.g., old deep ischaemic stroke was detected in 9.4% of studies), while haemorrhagic stroke subtypes were comparatively rare (0.2–2.9%). Tumour-related findings were detected in 0.4–4.1% of studies, depending on subtype. Regarding imaging sequences, T2 was the most frequently recorded (13.4% of studies), followed by T1 (8.3%) and FLAIR (7.8%). These NLP-derived labels formed the basis for the phenotypic exclusion criteria applied to the dementia cohort.

Of the 21,418 MRI studies requested, 21,197 were successfully received, with 221 being excluded for privacy reasons. These studies contained 128,257 series of which 73,457 were identified as T1, T2, or FLAIR; 18,681 series were localizers; 4,372 as unknown; 30,267 were other MRI sequences (DWI, SWI, T2*, etc.); and 1,464 series had a series description that did not differentiate T1 and T2*. Table 4 describes the filtering process making use of the DICOM labelling process, which resulted in the exclusion of 2,641 FLAIR, 11,820 T1, and 10,863 T2. After restricting the selection to the first chronological series for each study, 41,966 series were kept from 15,558 studies. Later, 277 studies were excluded when they failed to convert to NIfTI and subsequent process.

**Table 4:** Selection process based on the automatic labelling for the MRI cohort.

	Reason for exclusion						
Sequence	Baseline	Brain absent	PartialBrain	Other body parts present	Contrast or angio present	More than one series per study	Selected
FLAIR	16,871	−60	−211	−1,742	−628	−714	13,516
T1	27,627	−1,290	−4,670	−2,707	−3,153	−2,156	13,651
T2	28,959	−1,407	−4,828	−3,738	−890	−3,297	14,799

The “Baseline” column shows the number of series for each sequence type prior to exclusion. The “Selected” column shows the number of series kept after the exclusion process ready for analysis. Every column in between is associated with an excluding step and shows the number of series excluded. Non-brain scans, scans with only partial brain, brain scans including other body parts, scans with contrast, or angio scans were excluded iteratively. Finally, only one series of each sequence was kept per study.

The MRI studies requested originated from 35 hospitals across Scotland using 27 unique MRI scanner models were identified (14 models with <100 studies), with 60% of the studies using a Siemens model, 20% General Electric, and 20% Philips, and a handful from 2 other manufacturers. 94% of the scans were done with a 1.5 Tesla, and 6% a 3 Tesla MRI scanner.

The MRI cohort contained 8,145 cases (53.2% female) and 7,236 controls (54.1% female) (Table 5). The mean age at scan was 74 years. The mean time from scan to first mention of dementia was 5 years for cases, and the mean follow-up time for controls was 6 years 9 months. Of the 8,145 dementia cases, there was non-exclusive record of AD in 3,774, VaD in 3,386, unspecified dementia in 3,784, and other dementia types in 508. The mean number of hospitalizations in the year prior to scan was 1.1 (standard deviation ([SD] 1.52) for cases and 1.0 (SD 1.50) for controls. During the same period, the mean number of prescriptions was 15.4 for cases and 14.2 for controls.

**Table 5:** Distribution of subjects with mention of each dementia type and controls grouped by key characteristics.

		Alzheimer’s	Vascular	Other or rare	Unspecified	Controls
Sex	Female	2,057	1,681	207	2,047	3,917
	Male	1,717	1,705	301	1,737	3,319
Age in years	Mean (SD)	73 (8.8)	75 (8.7)	67 (9.3)	74 (8.9)	74 (9.0)
	40–50	36	32	19	38	87
	51–60	282	169	90	227	434
	61–70	968	706	179	813	1611
	71–80	1,694	1,503	182	1,664	3,182
	81+	794	976	38	1,042	1,922
SIMD	Mean (SD)	2.8 (1.3)	2.7 (1.3)	2.8 (1.3)	2.7 (1.3)	2.8 (1.3)
	1	719	768	102	842	1429
	2	732	650	102	724	1380
	3	753	635	94	703	1381
	4	909	691	113	780	1523
	5	363	242	42	325	804
Hospitalization 1 year before scan, mean (SD)		0.9 (1.4)	1.2 (1.5)	1.0 (1.6)	1.1 (1.5)	1.0 (1.5)
Prescriptions 1 year before scan, mean (sD)		14.5 (9.5)	16.1 (0.2)	14.3 (9.7)	15.6 (10.2)	14.2 (9.4)

SD: standard deviation; SIMD: Scottish index of multiple deprivation.

### SCANDAN: data validation and quality control

For simplicity, we refer to the results of the manual annotations as “annotations,” and the results of the automatic tools described in Fig. 4 as “labels.”

During the first round, 707 annotations were obtained from 713 images. Four images could not be read due to acquisition errors. Two images were only partially annotated due to visual perception errors and discarded. In 29 (4.5%) cases, image modality was wrongly annotated because the series contained only one slice (i.e., a localizer) or did not contain a brain. Of CTs, 24 (7.52%) were classed as “Unknown.” In the labelling process, we used the previously validated DICOM tag “Modality” (0008,0060) to identify CT and MRI series. The identification of the body parts was easier for the annotators than the modality or sequence type. “Unknown” was given for 23 (3.3%) series when annotators were questioned whether they contained a brain or not, 40 (5.7%) when questioned if the brain was acquired in full, and in 36 (5.1%) series the annotators could not assert whether there was another body part. Annotators could not identify the sequence type for 85 (12.0%) series and the presence of contrast in 104 (14.7%) series.

In this first round of annotations, the main disagreements between annotations and labels were the presence of non-brain images or localizers. For further analyses, the series labelled as “localizer” in the first round of annotations, defined as series with less than 15 slices, were ignored. If the annotation and label agreed on the absence of brain, the images were not re-annotated.

In the second round of annotations, images with an unknown sequence type (16 series), and those with disagreement between label and annotation (“T1,” “T2,” or “FLAIR,” 48 series), were re-annotated. Additionally, a subset of images was selected from 66 with partial agreement between at least one sequence label and the annotation, to validate commonly occurring combination of labels which were not similar (e.g., “T1” + “GRE” instead of “T1” + “T1”). Series that contained at least one mention of “T1,” “T2,” or “FLAIR” in either the annotation or the label were re-annotated for the presence of contrast when disagreement was found or when they were annotated as “Unknown.” Series with “Unknown” annotation for questions regarding brain presence (13 series), other body part presence (8 series), and whole brain (11 series) were also re-annotated. Disagreement between the annotation and the labels were also re-annotated, respectively 7,143 and 20 series. In case of a whole brain, the disagreement was ignored if other body parts were present in both the label and the annotation. In total, 143 series were re-annotated for the presence of brain and other body parts, and for full brain coverage. Additionally, there were 84 images re-annotated for sequence type and contrast.

To resolve conflict between the 2 re-annotators, or between them and the labelling tools, 27 series were then annotated a third time. Some conflicts could not be resolved, such as 7 images having the same “Series Description” (0008,103E) tag value, and thus the same label. Three of them were identified as T1 and 4 as T2* by the 2 annotators in agreement.

Between each round of annotation, the regular expressions used by the labelling tools were updated to reflect previously unknown, and to solve conflicting information and errors.

The results of the labelling tools compared to the final annotation as ground truth were very good. The true positive rate ranged from 87 to 97% and the positive predictive value from 81 to 99% (Table 6). For consistency with our metrics, we evaluated the absence of contrast and other body part, respectively, as “positive.” This value excludes localizers for sequence type, and series without the presence of brain for “whole brains.” The lower precision for detection of other body parts is explained by the lack of mention of any parts in the different DICOM tags, sometimes due to missing data, as well as the detection of some other head parts, such as the jaw, without mention of the brain, which often, but not always, indicate non-brain scans. The lower recall for the absence of contrast is caused by the low number of studies that used intravenous (IV) contrast. During a scanning session that used IV contrast, a first image will normally be captured free of contrast, prior to the injection; however, the “Study Description” (0008,1030) will indicate the presence of IV contrast nonetheless for this first series, as was commonly found.

**Table 6:** The metrics of the comparison between the automated labels and the manual annotation as ground truth for image characteristics.

Algorithm identified image characteristics	Recall (%)	Precision (%)
Sequence type	94.1%	98.6%
Study contains brain	97.1%	91.0%
Absence of other body parts	93.1%	81.9%
Whole brain in study	96.4%	95.6%
Absence of contrast in study	87.3%	95.4%

### SCANDAN contribution and BHD data

The SCANDAN project produced data which were added to the BHD. In addition to the 1.2 million brain studies from 830,000 patients, the 1.8 million radiological reports and the 356 million EHR available as raw data, researchers can also access 5 additional tables: (1) summary of the valid radiological reports generated by the NLP; (2) dementia phenotyping table, with dementia subtype probability and date of diagnosis; (3) patient history, curated and listing all relevant information from all EHR; (4) the manual annotations of the 708 series; (5) the labelling of the 21 K MRI of the SCANDAN cohort for MRI sequence, body part imaged, brain fullness, and contrast presence. The latter is planned to be expanded to the totality of MRI and then CT scans.

### Permissions and governance

The SCANDAN application to the PBPP, which included an industry partner and aimed to develop an AI algorithm, required 210 days spanning 4 iterations for approval from the initial submission and over 17,000 words across 33 pages.

**Figure 5:**
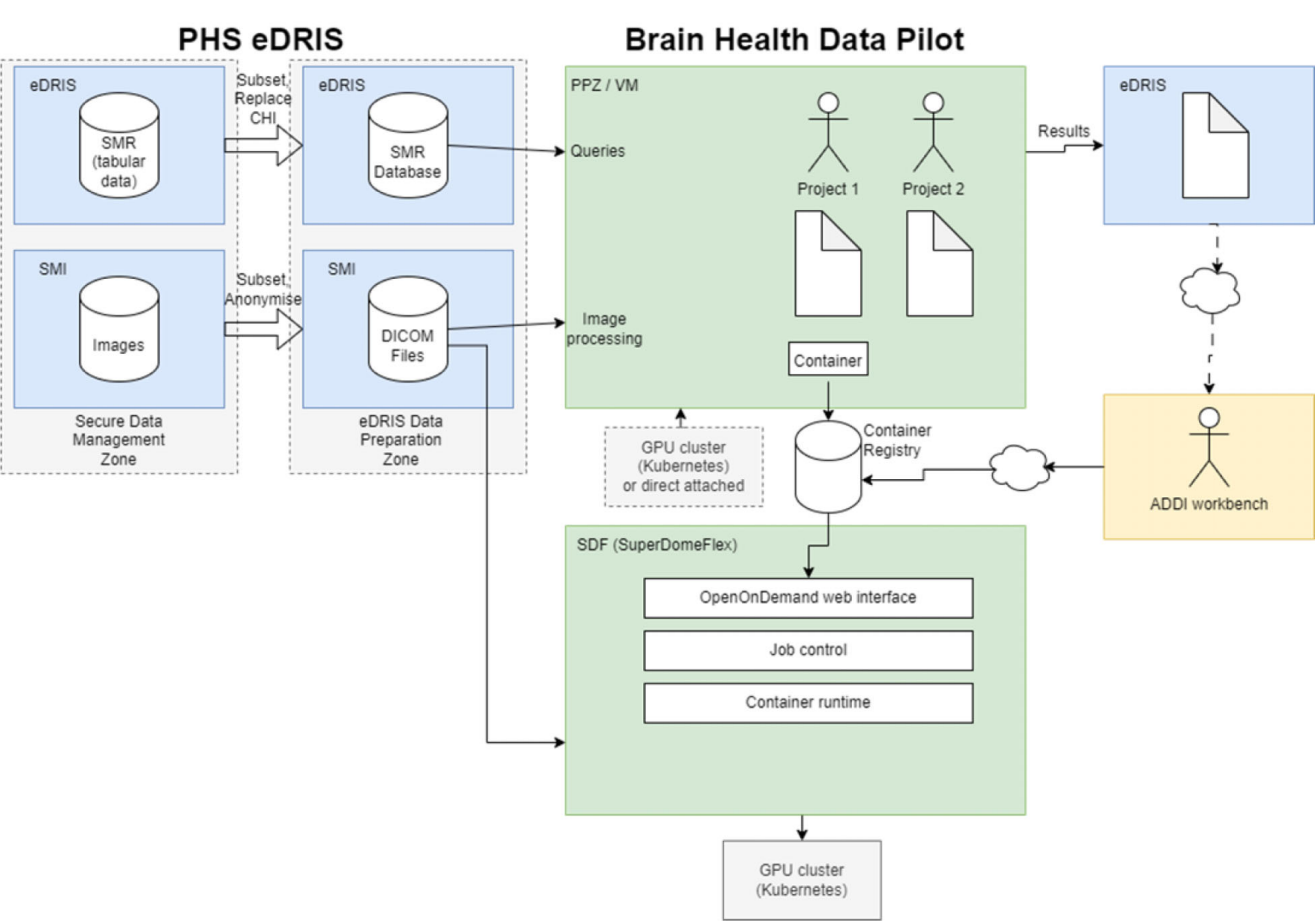
Diagram to illustrate the data flow and data linkage process in the BHD service. eDRIS: Electronic Data Research and Innovation Service, SMR: Scottish Morbidity Record, CHI: Community Health Index number, PPZ: privacy-preserving zone, VM: virtual machine, DICOM: Digital Imaging and Communications in Medicine.

However, with the development of the BHD, a researcher can now apply to PBPP to access these data with a shorter application and streamlined process. The data flow and linkage process for the BHD framework are schematically illustrated in Fig. 5. Researchers can log into a workspace running in the NSH with data and tools to perform analysis. To run externally developed tools, they can build a container outside the NSH and pull it from a public registry after approval. It should be noted that no data leaves the NSH during this process. The TRE is divided into several zones (Fig. 5). The blue zones, where eDRIS store the data, are not accessible to the researchers. Researchers have access to green zones with access to subsets of the data, as defined by their access permission.

PHS eDRIS will prepare suitable subsets of the data for a particular research group and copy it to their working space. Any results required outside the NSH, e.g., for publication, are subject to disclosure control performed by eDRIS.

## Discussion

The BHD is a large-scale, curated brain imaging clinical dataset relevant to dementia research that is available to researchers via moderated public access. The dataset offers several advantages in addition to its large size: clinical relevance, long-term follow-up, co-location with a GPU cluster in a safe haven, greater population representativeness compared to many research cohorts, and accessibility for clinical researchers. The resource continues to grow in data size and computing power.

Working with health systems data presents challenges. One of them is the time taken for governance approval. In our case, if governance had been applied for once funding had been awarded, it would have represented 58% of a 1-year postdoctoral award. This would not only impact negatively on career development of the post holder but also delay the project goals, an issue addressed by the streamlined process of the BHD. Data provision was initially constrained by the limitations of the virtual machine environment, limited staff availability, and increased procedural complexity resulting in delayed access to imaging data and complicating project planning. The experience gained through the SCANDAN project, which piloted the access to the data, allowed PHS to streamline the governance process and improve the data provisioning to future projects. It is important, however, to note that all research outputs generated within the NSH must undergo review by PHS staff prior to release.

Most imaging research is based on uniformly acquired research data. In contrast, clinical scans acquired in routine free-at-the-point-of-service healthcare are sometimes incomplete, may be obscured by movement or other artefacts, show signs of non-relevant pathologies, may have been obtained with non-standardized protocols, and on different machines. However, such real-world data with inherent variability are essential for the development of software tools suitable for robust applications in clinical practice where such heterogeneity is the norm.

Using EHRs for dementia diagnosis has limitations. Currently, primary care data are unavailable through PHS and thus cannot be provided by the BHD. Hence, we relied on recorded diagnosis after an inpatient stay or death. Hospital and death records under-ascertain (false negatives) dementia in the short term and have modest reliability for dementia subtypes [22]. However, they have also previously shown high positive predictive value for all dementia diagnoses [14]. Referral reasons for scan acquisitions are not currently available, although further NLP work with reports could achieve this.

The use of head scans does raise privacy concerns due to facial recognition risks. We have mitigated these by working only in a safe haven environment, examining only brain slices, prohibiting facial reconstruction, limiting access to approved researchers, who accept the restrictions and conditions of working in the NHS specified in the eDRIS User Agreement, which includes PHS strictly checking all outputs from the secure environment, to exclude any identifiable data. Future work aims to further mitigate privacy risks by limiting the need for direct human access to data, for example, by implementing software via containers. However, this work needs training of the research community, better labelling of metadata (so the data are truly FAIR), and further development of technology within the NSH environment.

There are many opportunities for further linkage to other datasets (e.g., community retinal imaging [23]). Such work will require further engagement with public contributors, use of federated analysis and federated learning with ongoing adoption of tools and techniques to assess disclosure risks of different AI models.

The SCANDAN project piloted the access to the data now provided through the BHD. While its primary goal was to establish a proof of concept for dementia classification using clinical data, it produced several secondary outputs which are now available to other researchers using the BHD data. As more projects will use the data, additional output will be added, compounding with time to an unvaluable resource for brain imaging research. Researchers can access the BHD data by applying to PBPP via eDRIS. Proposals must demonstrate a clear public benefit, and researcher–generated outputs must be added back to the dataset so every project strengthens the next. We strongly encourage cross–group collaboration. The resources available through the BHD are growing in terms of data availability, storage capacity, and computing power that are provided to researchers. We hope that this, and similar global initiatives, will ultimately contribute to improve the brain health of people worldwide.

## Abbreviations

ADDI: Alzheimer’s Disease Data Initiative; AI: artificial intelligence; AD: Alzheimer’s disease; BHD: Brain Health Data; CRAN: comprehensive R archive network; CSV: comma separated values; CT: computerized tomography; DICOM: Digital Imaging and Communications in Medicine; DSPT: NHS England’s Data Security and Protection Toolkit; eDRIS: electronic Data Research and Innovation Service of Public Health Scotland; EHR: electronic health records; EPCC: Edinburgh Parallel Computing Centre; EdIE-R: Edinburgh Information Extraction for Radiology; FAT-SAT: fat saturation; GE: General Electric; GLAD: Generative model of Labels, Abilities, and Difficulties; GPU: graphical processing unit; GRE: gradient echo; GUI: graphical user interface; HDRUK: Health Data Research; UKMRI: magnetic resonance imaging; NHS: National Health Service; NIfTI: Neuroimaging Informatics Technology Initiative format; NLP: Natural language processing; NSH: National Safe Heaven; NRS: National Records of Scotland; PBPP: Public Benefit and Privacy Panel for Health and Social Care; PHS: Public Health Scotland; PIS: Prescribing Information System; PyPI: Python package index; SCANDAN: SCottish AI in Neuroimaging to predict Dementia and Neurodegenerative Disease; SD standard deviation; TRE: Trusted Research Environment; VaD: vascular dementia; WP: work package.

## Supplementary Material

giag072_Authors_Response_To_Reviewer_Comments_original_submission

giag072_Authors_Response_To_Reviewer_Comments_revision_1

giag072_GIGA-D-25-00442_original_submission

giag072_GIGA-D-25-00442_revision_1

giag072_GIGA-D-25-00442_revision_2

giag072_Reviewer_1_Report_original_submission Reviewer 1 -- 12/14/2025

giag072_Reviewer_1_Report_revision_1 Reviewer 1 -- 4/13/2026

giag072_Reviewer_1_Report_revision_2 Reviewer 1 -- 5/30/2026

giag072_Reviewer_2_Report_original_submission Reviewer 2 -- 12/21/2025

giag072_Reviewer_2_Report_revision_1 Reviewer 2 -- 4/15/2026

giag072_Reviewer_2_Report_revision_2 Reviewer 2 -- 5/10/2026

## Data Availability

Application to use these data should be made to the Electronic Data Research and Innovation Service (eDRIS) of NHS Scotland. Applicants will be supported through the application process by a co-ordinator. All applications are reviewed by the NHS Scotland Public Benefit and Privacy Panel for Health and Social Care (HSC-PBPP).
