# Supplementary material for: A large dataset of brain imaging linked to health systems data: curation and access to a whole system national cohort from NHS Scotland
Source: Gigascience. 2026 Jun 9;15:giag072. doi: 10.1093/gigascience/giag072 (PMC13347094; doi:10.1093/gigascience/giag072)
Supplement: giag072_GIGA-D-25-00442_revision_1 [file giag072_giga-d-25-00442_revision_1.pdf]

GigaScience

A large dataset of brain imaging linked to health systems data: curation and access to a whole system national cohort from NHS Scotland

--Manuscript Draft--

**Manuscript Number:**	GIGA-D-25-00442R1
**Full Title:**	A large dataset of brain imaging linked to health systems data: curation and access to a whole system national cohort from NHS Scotland
**Article Type:**	Data Note
**Funding Information:**	Eisai — Dr Michael P J Camilleri<br>LifeArc — Dr Michael P J Camilleri<br>Gates Ventures — Dr Michael P J Camilleri<br>HDRUK — Dr Michael P J Camilleri<br>Alzheimer's Disease Data Initiative — Dr Michael P J Camilleri
**Abstract:**	We present the design and implementation of a data curation framework to generate a large-scale clinical brain imaging dataset suitable for artificial intelligence (AI) enabled image analysis. The dataset is accessible through the Brain Health Data (BHD) initiative, which includes approximately 417,000 magnetic resonance imaging (MRI) and 846,000 computerized tomography (CT) head scans, linked electronic health records (EHRs), and associated free-text imaging reports from clinical practice between 2010 and 2018 in Scotland, exceeding 185 TB in size. The data curation framework was developed during the SCottish AI in Neuroimaging to predict Dementia and Neurodegenerative Disease (SCANDAN) study, which used a subset of 45,000 MRI from the BHD for dementia prediction.<br>We describe the processing of the BHD metadata and our multilabel classification output. We discuss the strengths of the BHD, including clinical relevance thanks to its unprecedented scale, population-wide representativeness of a national free-at-the-point-of-delivery healthcare, long-term follow-up to neurodegenerative disease, and real-world variability. We describe the challenges and lessons learnt in developing a framework to curate data, including the time needed to obtain permissions, the need for easily accessible, secure, responsive and affordable computational environments, the variability of clinical data, and the challenge of extracting linked clinical data and images at scale.<br>This resource will be crucial for clinical research, fostering the development of personalized medicine approaches, and fast-tracking the implementation of AI models in clinical workflows. We encourage the use of the BHD data through a streamlined application to the Public Benefit and Privacy Panel for Health and Care via the Data Research and Innovation Service of Public Health Scotland (eDRIS).
**Corresponding Author:**	William Whiteley<br>The University of Edinburgh Edinburgh Neuroscience<br>University of Edinburgh, UNITED KINGDOM
**Corresponding Author Secondary Information:**	
**Corresponding Author's Institution:**	The University of Edinburgh Edinburgh Neuroscience
**Corresponding Author's Secondary Institution:**	
**First Author:**	Michael P J Camilleri
**First Author Secondary Information:**	
**Order of Authors:**	Michael P J Camilleri<br>Dorian Gouzou<br>Salim Al-Wasity

Powered by Editorial Manager® and ProduXion Manager® from Aries Systems Corporation

	Muthu R K Mookiah
	María Valdes Hernandez
	Bea Alex
	Sotirios A. Tsaftaris
	Andrew Brooks
	Ruairidh MacLeod
	Honghan Wu
	Brenda Bauer
	Claire Grover
	Parminder Reel
	Susan Krueger
	Richard Tobin
	J. Douglas Steele
	Grant Mair
	Joanna Wardlaw
	Alexander Doney
	Emanuele Trucco
	William Whiteley
**Order of Authors Secondary Information:**	
**Response to Reviewers:**	A large dataset of brain imaging linked to health systems data: the curation and access to a whole system national cohort from NHS Scotland<br><br>Response to Reviewers<br><br>We thank the reviewers for the time they spent and their helpful comments. Both of them pointed out inconsistencies in the scope and objectives of the paper, and their insight was very valuable in reframing the narrative of this note to align with our original goals. We also thank the editor for their time they allocated to our paper.<br><br>Reviewer 1:<br>1. The abstract and introduction emphasize the full raw scale of the dataset (hundreds of thousands of MR/CT scans) and the curation framework, whereas the Results section focuses primarily on a derived dementia-matched cohort and a subset of retrieved studies. Please clearly separate these aspects and report, with explicit counts, the total number of raw imaging studies available in SMI (MRI and CT), as well as the number of unique patients.<br>answer: Agree - revised<br>-ABSTRACT: We clarified the abstract by adding the size (45K scans) of the SCANDAN cohort and the type of images used (MRI). We have made clear the data is from a subset of the BHD data (Abstract, par. 1).<br>-INTRODUCTION: We clarified the data available in the BHD (MRI, CT, electronic health record (EHR) and free text radiology report). We also clarified the distinction between the BHD data and the SCANDAN pipeline, introducing clearly the aims of both and their relationship (Introduction, par. 5).<br>-METHODS: We added a new subsection called “Data Availability” which lists the number of scans, report and EHR available through the BHD, as well as the number of patients. We included the count for different subset of data sources, such as the different EHR.<br>-FIGURE: We added Figure 5, which shows the distribution of slices per series, emphasising the scale of the dataset. (Methods, Data Availability)<br>-RESULTS: We changed the previous subsection “Dataset description” to “SCANDAN Output Dataset”, to now reflect properly the distinction between the BHD data (the raw

	dataset) and the results of the curation framework.<br>-NARRATIVE: The addition of those two subsections, as well as the reorganisation of the introduction was done to resolve the first point of the reviewer here, highlighting the difference in focus within the paper between the introduction and the results. To this end, we also renamed the Methods subsection relevant to SCANDAN by prefixing them with SCANDAN. Overall, our change aim to make sure the aim of the paper is clear; the introduction of the BHD, the presentation first pilot project carried with its data, and the additional data which it provided to the BHD<br><br>2. Please provide more detailed information on the subset of scans that successfully passed the pipeline filters (e.g., T1/T2/FLAIR sequences, whole-brain coverage, absence of contrast or angiography). Although Table 5 partially addresses this, its framing could be clearer—for example, please clarify whether the "Final" category corresponds to the number of research-ready MRI series per sequence.<br>answer: Agree – revised<br>-TABLE: We renamed the last column of table 5 from “final” to selected. We also expanded the caption to clarify the meaning of each column.<br>-RESULTS: We referenced Table 5 in the results, providing additional precision on the number of scans excluded by the label and the number of scans selected as the "first chronologically within the study" and listing the total amount of studies. (Results, SCANDAN Output Dataset, par. 3)<br><br>3. Please clarify what data are currently accessible to external researchers through eDRIS/BHD. For instance, does access include raw DICOM files, NIfTI conversions, derived labels, structured report phenotypes, or predefined example cohorts?<br>answer: Agree – revised<br>-METHODS: The “Data Availability” subsection lists all the data contained within the BHD<br>-RESULTS: A new section named "SCANDAN Contribution and BHD data" was added, explaining which data are available within the BHD (referencing the one listed in the "Data Availability" subsection), as well as listing the 5-output produced by SCANDAN. It describes each of these outputs, including their scope (whether it refers to the whole dataset, or the SCANDAN cohort). Each of these outputs are described in more details in the other Results subsection "SCANDAN Output Dataset".<br><br>4. The manuscript describes rule-based parsing of multiple DICOM tags using regular expressions, together with MRI parameter checks (e.g., TR/TE/TI), to define sequence labels. Please provide a complete list of the regular expression patterns used for sequence, body-part, angiography, and contrast identification, along with information on versioning and how updates were handled across iterative annotation rounds.<br>answer: Partially Agree - revised<br>-A paragraph was added to clarify the creation of the regular expressions (based on prior in-house study, expanded to include variation from all the hospitals, then using the most common occurrence of each tag to make sure nothing is widely excluded, before finally using the manual annotations to refine them). (Methods, SCANDAN: Identification of MRI scan type and sequence, par. 5)<br>-We provided a link to a GitHub repository containing the regular expression (Methods, SCANDAN: Identification of MRI scan type and sequence, par. 2)<br><br>5. Please clarify how the criterion "3D image volume > 5 litres" is computed and provide a rationale for the choice of this threshold.<br>answer: Agree – revised<br>-We clarified how the threshold was chosen based on empirical test, from a previous study using similar data. We compared the volume to the annotations to assert it was correct. We added a citation to it (Singh Reel et al). (Methods, SCANDAN: Identification of MRI scan type and sequence, par. 1).<br><br><br>6. The expert annotation study (n = 713 scans across MRI and CT, with 100 overlapping cases and GLAD adjudication) is a strong component of the work. Please clarify how these 713 scans were sampled (e.g., randomly across sites, scanners, and years; stratified by modality; or enriched for challenging or ambiguous cases).<br>answer: Agree - revised<br>-We clarified that the data was randomly sampled by EPCC to be provided as example

Powered by Editorial Manager® and ProduXion Manager® from Aries Systems Corporation

data (Methods, SCANDAN: Data validation and quality control, par. 2)

7. The manuscript currently lacks a concise summary of key imaging metadata. For neuroimaging researchers, it would be highly valuable to include aggregate summaries of:
(1) scanner vendors/models and magnetic field strengths (for MRI), and their distribution across sites and over time;
(2) typical voxel sizes, slice thickness distributions, and acquisition planes for the retained sequences; and
(3) rates of partial brain coverage or localizer scans.
answer: Partially Agree - revised
-We added the number of localiser (along other labels) (Results, SCANDAN Output Dataset, par 3.)
-We added the amount of hospitals site, MRI devices and vendors contained within the data delivered to SCANDAN (21K studies MRI). (Results, SCANDAN Output Dataset, par 4.)
-As a proxy to slice thickness/voxel sizes, we added the distribution of slices per series within Figure 5. This data is obtained on the whole data from the BHD, including CT
-The information asked could not be provided on the entire scale of the BHD for some case due to the metadata not being available. (Methods, Data Availability)

8. Please indicate whether defacing is performed or feasible within the Trusted Research Environment for MR T1-weighted volumes, and whether defacing is mandatory for any form of data export (recognizing that raw images may not be exported, but derived images could be generated).
answer: Partially Agree – partial revision
-No defacing was performed.
-No unconsented images can be exported from the secure data environment.
-The discussion already provides a paragraph on privacy concern (Discussion, par 5.). It also includes two mentions that any data taken out of the Trusted Research Environment need to be validated by PHS (Discussion, par. 2 & par. 5).
-We added to the discussion the mention that PHS review the data to “excludes any identifiable data.” (Discussion, par 5).

9. The manuscript alternates between the terms "scan," "study," "series," and "image." Although these are defined earlier, some later statements could be clarified to avoid ambiguity. For example, the phrase "each study (i.e., one per person)" could be interpreted as implying that each individual has only one study, whereas later sections refer to selecting the "earliest studies associated with these subjects." Please clarify whether "one per person" reflects a deliberate selection criterion or an inherent property of the source data.
answer: Agree - revised
-We clarified how the linkage was done by eDRIS and EPCC (not us) and how it linked the study (scans) to "one person" (i.e. a patient) from the EHR (Methods, Data Sources, par. 1)

Reviewer 2:
1. Several figures aim to provide an overview of the data curation and processing pipeline, but the relationship between the overall SCANDAN project, the specific scope of this Data Note, and the individual curation steps described in the Methods is not always clear. In particular, Figure 1 illustrates the work packages of the overall SCANDAN project; however, it is not immediately clear how these relate to the specific focus of this manuscript. While SCANDAN aims to develop and evaluate dementia classifiers, this paper appears to focus primarily on the data curation and preparation framework. I encourage the authors to clarify in the text and figure legend which components of Figure 1 are directly covered in this manuscript, and which are shown for broader project context.
answer: Agree – revised
-ABSTRACT: we clarified the data curation pipeline was linked to the SCANDAN project (Abstract, par. 1), and was processing a subset of the BHD MRI data, adding the details on the relationship between the two projects (Abstract par. 1).
-INTRODUCTION: We reformulated the definition of the BHD, by focusing on the data it provides. We then explained SCANDAN, a project aiming to perform dementia classification, was the first to use the BHD data. We further explained that SCANDAN

produced several outputs when processing the data which were added to the BHD, such as parsing of the radiological text report with NLP, or the dementia phenotyping. Finally, we summarised the relevant part of SCANDAN which are discussed in this paper, aiming to create a research ready dataset, demonstrating what is available within the BHD (Introduction par. 5).
-METHODS: We added a new subsection "SCANDAN Project", referencing Figure 1, which replace the previous explanation from the introduction, and explains in detail how each part interact together, clarifying the SCANDAN project (Methods, SCANDAN project)
-RESULTS: We added a new subsection "SCANDAN Contribution and BHD data" showing the relationship between SCANDAN output and the BHD. The SCANDAN output will be available through the BHD for other researcher, significantly speeding up their research by saving them the time to carry the same, complex, data curation and processing (Results, SCANDAN Contribution and BHD data).
-NARRATIVE: The addition of these 2 subsections, as well as the reorganisation of the introduction was done to address the reviewer point on the confusing relationship between SCANDAN and the BHD, highlighting the goal and scope of the paper were not clear. The BHD provides access to data and easier governance, and the SCANDAN project uses this data, demonstrating its usefulness, providing important demographic information on the dataset, and provided several key outputs which were added to the BHD. We hope those changes clarify the interlinked relationship the BHD and SCANDAN have.

2. Multiple data selection and refinement steps are described across the manuscript, including DICOM-based filtering, NLP-driven exclusions, manual annotation, and iterative re-annotation, but their sequencing and respective roles can be difficult to follow. Figure 4 appears to attempt to bring these elements together, yet it is not explicitly referenced in the text, and its legend lacks sufficient explanation to map the visual elements (including the numbered markers) to the corresponding methodological steps. More explicit cross-referencing between text and figures, and a concise summary (e.g. a short paragraph, table, or expanded figure legend) outlining the full sequence of automated and manual steps, would help clarify how each contributes to the final curated dataset.
answer: Agree – revised
-FIGURE: Figure 4 was referenced within the text (Methods, SCANDAN: Identification of MRI scan type and sequence, par. 1). Its caption was extensively rewritten to explain what each colour means, what each block does, and in which order they were performed. (Figure 4, Methods, SCANDAN: Identification of MRI scan type and sequence).
-METHODS: the new subsection "SCANDAN Project" also clarified the order in which the SCANDAN work package were used and how they interacted together, making it clearer what was done and when (Methods, SCANDAN Project).
-RESULTS: The subsection "SCANDAN Output Dataset" replace the previous "Dataset description" and provides a detailed description of the exclusion steps, with input and output numbers, referencing the summary Tables (3, 4, 5) which list the information more concisely (Results, SCANDAN Output Dataset)
-NARRATIVE: We reorganised the Methods, moving "SCANDAN: Identification of MRI scan type and sequence" after "SCANDAN: Data validation and quality control". We also clarified which parts were linked to SCANDAN by prefixing the subsection title with "SCANDAN".

3. Data sources section: the description of data linkage and processing (e.g. "We linked each study…", "All data was processed and stored…") would benefit from clarification on which steps are automated and which require manual intervention.
answer: Disagree – minor revision for clarification
-We clarified how the linkage was done by eDRIS and EPCC (not us) and how it linked the study (scans) to "one person" (i.e. a patient) from the EHR (Methods, Data Sources, par. 1)
-The storage, just like the linkage, is performed by ePCC and is inherent to the National Safe Haven. If the reviewer is referring to the rest of the processing described in the Methods, we believe each relevant section highlight the steps were done automatically, save for the annotation.

4. NLP tool: it would be useful to clarify whether the NLP tool is openly available (e.g.

as code or a container), as this could be of significant value to the community. In addition, the sentence on page 6 describing the "enhancement of the detection of section boundaries" is highly technical and difficult to follow in this context; a brief rephrasing or explanation of what is meant by section boundaries would help. More generally, a short high-level description of the NLP inputs, outputs, and how these outputs are used within the broader framework would improve readability.
answer: Agree – revised
-We added a citation with a link to EdIE-R (Methods, SCANDAN: Natural language processing of brain imaging reports, par. 4).
-We re-phrased the sentence about section boundaries to simplify it, explaining we refer to the boundary between the report and the clinical history (Methods, SCANDAN: Natural language processing of brain imaging reports, par. 3).
-We provided an additional paragraph summarising the NLP methodology to select the radiological report used as input and their filtering (Methods, SCANDAN: Natural language processing of brain imaging reports, par. 5).
-We also provided a summary of the NLP report output in the Results, listing some of the finding, as well as the numbers of report processed (Results, SCANDAN Output Dataset, par. 2)

5. Phenotyping dementia: this section appears to be missing a reference to Table 1. In addition, Table 1 would benefit from a more informative legend explaining how it should be interpreted. For example, it is unclear why a BNF code is reported only for Alzheimer's disease. While the use of wildcards is understandable, the rationale and intended use of these wildcards should be explicitly stated in the table legend.
answer: Agree – revised
-We added two references to support out method for phenotyping dementia (Doney et al (2019), Doney et al (2025)). We also clarified why the BNF code is only used for Alzheimer's as it's the only that have specific medication. (Methods, SCANDAN: Phenotyping Dementia)
-We added the reference to Doney et al (2025) to the caption of Table 1, and clarified the meaning of the wildcards as well as the reason for their usages, i.e., to that all child-codes in the hierarchy are included (Table 1, Methods, SCANDAN: Phenotyping Dementia)
-The reference to Table 1 was fixed.

6. Cohort building: the description of cohort selection (e.g. "We first selected… gone through the pipeline… and had no NLP label") would benefit from clearer specification of the order of steps, and of which steps are automated versus manual. Explicit input/output descriptions and a reference to a visual flowchart would help clarify this process.
answer: Partially agree – revised
-The order of the steps for the cohort building was clarified, by removing some steps happening later, which were introducing confusion by being mentioned this early. Specifically, we removed the mention to the image filtering based on the DICOM tag. We kept the exclusion using NLP mention of tumour or strokes. (Methods, SCANDAN: Cohort Building, par. 2)
-The cohort building input, output and the number of excluded samples, referenced in Table 3, were added to the results (Results, SCANDAN: Data).
-We did not add another flowchart. However, we expect the rest of the modification on the paper, about signposting, cross-referencing, and clarification of the order of the SCANDAN project, will clarify the order in which each step was done. None of these steps were "manual".

7. DICOM tags: similarly, the description of how DICOM-derived labels are combined with NLP outputs would benefit from clearer sequencing and explicit reference to a flowchart illustrating the full pipeline.
answer: Agree - revised
-A reference to Figure 4 has been added (Methods, SCANDAN: Identification of MRI scan type and sequence, par. 1). The caption of Figure 4 has been improved to explain the sources of the data, illustrating exactly which tags are used for which task, through which methods. It also shows the combination of the NLP and the pattern matching methods using DICOM (Figure 4, Methods, SCANDAN: Identification of MRI scan type and sequence).
-We added a sentence about how the NLP is used to validate the presence of a

Powered by Editorial Manager® and ProduXion Manager® from Aries Systems Corporation

	sequence within a study (Methods, SCANDAN: Identification of MRI scan type and sequence, par. 4).<br><br>8. Data validation and quality control: Table 2 appears to relate to this section but is only explicitly referenced in the Results section. It is also unclear what components are included in the "automated labelling process described above" (e.g. DICOM-based rules only, NLP-derived information, or both). Clarification would also be helpful on what constitutes the "ground truth" used for this comparison, in particular whether the results in Table 2 reflect the final labels after all rounds of annotation, GLAD aggregation, and expert adjudication.<br>answer: Disagree – partially revised anyway<br>-Table 2 is only explicitly referenced in the results section as it shows the results of the Data validation and quality control. We clarified in the methods that we are reporting in the results the final comparison of the labelling and the annotation (Methods, SCANDAN: Data validation and quality control, par. 3).<br>-The automatic label refers the output of the tools described in the section "SCANDAN: Identification of MRI scan type and sequence" (now "next section" previously "above"). There are one group of labels described in this section, described in paragraph 1, with the process to obtain them described in paragraph 1 to 3. Those labels described match the one which were annotated. We hope the clarification concerning NLP usage and the expanded caption for Figure 4, now properly referenced in the relevant section, will explain that the label compared are the output of the agglomeration of those.<br>-We clarified the caption of Table 2 to reflect that the manual annotation is used as ground truth to assess the labelled obtained automatically (Table 2, Results, SCANDAN Output Dataset)<br>-We also added clarification regarding the comparison between the automatic tools and the manual annotations using the final versions (Methods, SCANDAN: Data validation and quality control, par 3.; Results, SCANDAN: Data validation and quality control, par 7.)<br><br>9. Use of GLAD: the role of GLAD in the workflow would benefit from further clarification. Specifically, it would be helpful to briefly state what inputs are provided to GLAD, what outputs are produced, and how these outputs are used in the evaluation of the automated labelling. It is currently unclear whether the results in Table 2 are based on GLAD-inferred labels or on subsequent manually adjudicated labels.<br>answer: Agree – revised<br>-We updated Table 2 caption to reflect that the ground truth is the manual annotation (Table 2, Results, SCANDAN: Data validation and quality control)<br>-We updated the explanation of GLAD usage as it had been previously erroneous. GLAD was not used to infer automatic label but used on the automatic label. It used the overlapping 100 annotations as "ground truth" and corrected the rest of the annotation (Methods, SCANDAN: Data validation and quality control, par. 2 & 3).<br>-We hope the clarification of how GLAD was used will answer the reviewer comment and may clarify some of their other comments. We thank the reviewer for pointing out the confusion.<br><br>10. Results of data validation and quality control: again, clarification on what is included under "automated tools" would help ensure consistency with the workflow described in the Methods. The authors might also consider illustrating the iterative annotation and adjudication process in a dedicated flowchart.<br>answer: Disagree – partially revised anyway<br>-We added a mention of Figure 4 in the results section to ensure the automatic tools and labels are referenced (Results, SCANDAN: Data validation and quality control).<br>-We added a sentence mentioning how we report the final comparison between the automatic tools and the label (Methods, SCANDAN: Data validation and quality control, par. 3)<br><br>11. Discussion: the authors note that the dataset will continue to expand in size; a brief summary of future plans for cohort growth and linkage would be of interest.<br>answer: Agree - revised<br>-We expanded on the plan to add NIfti and other processed images available to other project, in a similar fashion to what SCANDAN provided with the metadata and table output (Discussion, par. 7)<br>-We also clarified that one of the output of SCANDAN, the sequence/body/contrast/...

Powered by Editorial Manager® and ProduXion Manager® from Aries Systems Corporation

	labelling, was carried only on the 21K study from the SCANDAN sub cohort, but are planned to be expanded to the rest of the data in the BHD (Results, SCANDAN Contribution and BHD data)<br><br>12. Phenotyping dementia - a reference appears to be missing.<br>answer: Agree - revised<br>-Reference to table 1 fixed<br><br>13. Figure 3 legend: consider expanding it to include the explanation of colours and acronyms currently provided only in the text.<br>answer: Agree - revised<br>-Figure 3 legend now updated to: Figure 3. Diagram to illustrate the data flow and data linkage process in the Brain Health Data service (BHD). eDRIS Electronic Data Research and Innovation Service (eDRIS), SMR: Scottish Morbidity Record, CHI Community Health Index number, PPZ: privacy preserving zone, VM: virtual machine, DICOM: Digital Imaging and Communications in Medicine<br><br>14. Page 11: "sizer" appears to be a typo and should read "size".<br>answer: Agree - revised
**Additional Information:**	
**Question**	**Response**
Are you submitting this manuscript to a special series or article collection?	No
**Experimental design and statistics**<br><br>Full details of the experimental design and statistical methods used should be given in the Methods section, as detailed in our Minimum Standards Reporting Checklist. Information essential to interpreting the data presented should be made available in the figure legends.<br><br>Have you included all the information requested in your manuscript?	Yes
**Resources**<br><br>A description of all resources used, including antibodies, cell lines, animals and software tools, with enough information to allow them to be uniquely identified, should be included in the Methods section. Authors are strongly encouraged to cite Research Resource Identifiers (RRIDs) for antibodies, model organisms and tools, where possible.<br><br>Have you included the information requested as detailed in our Minimum	Yes

Standards Reporting Checklist?	
**Availability of data and materials**<br><br>All datasets and code on which the conclusions of the paper rely must be either included in your submission or deposited in publicly available repositories (where available and ethically appropriate), referencing such data using a unique identifier in the references and in the “Availability of Data and Materials” section of your manuscript.<br><br>Have you have met the above requirement as detailed in our Minimum Standards Reporting Checklist?	Yes
GigaScience has policies and guidelines in place for the use of generative AI-writing tools such as ChatGPT. If you have used such writing tools to assist with writing the manuscript this must be declared and cited in the text. Authors should not list AI-writing tools and other AI-assisted technologies as an author or co-author and should acknowledge that they are fully responsible for text generated or refined by AI-writing tools.<p><br><br>A summary of use (particularly in the introduction or among methods) needs to be included at the end of the paper, and the outputs should also be included as a supplementary file hosted in GigaDB or other open repositories. Please read our guidelines for more information. <p><br><br>By submitting to GigaScience, you are aware of the journal's AI-writing tools policy, and if you have declared use of such tools below, you have acknowledged this where appropriate in your manuscript and have made a summary of use and outputs available. <p><br><b>AI-assisted writing tools have been used in the preparation of this manuscript?	No

Revised manuscript with markup
Click here to access/download;LaTeX - Main Document (TeX file);Methodology_v10_final_track_change.docx

A large dataset of brain imaging linked to health systems data: the curation and access to a whole system national cohort from NHS Scotland

Michael P J Camilleri[1,2]*

Dorian Gouzou[3]*

Salim Al-Wasity[4]

Muthu R K Mookiah[4]

María Valdes Hernandez[3]

Bea Alex[5]

Sotirios A. Tsaftaris[2]

Andrew Brooks[6]

Ruairidh MacLeod [6]MacLeod[6]

Honghan Wu[7,8]

Brenda Bauer[3]

Claire Grover[5]

Parminder Reel[9]

Susan Krueger[9]

Richard Tobin[5]

J. Douglas Steele[4]

Grant Mair[3]

Joanna Wardlaw[3,10]

Alexander Doney[11]

Emanuele Trucco[1]**

William Whiteley[3,7,12]**

[1] Computing, School of Science and Engineering, University of Dundee, Dundee, UK.

[2] School of Engineering, University of Edinburgh, Edinburgh, UK.

[3] Institute for Neuroscience and Cardiovascular Research, School of Medicine, University of Edinburgh, UK.

[4] School of Medicine, Ninewells NHS and University Hospital, Dundee, UK.

[5] School of Informatics, University of Edinburgh, Edinburgh, UK.

[6] Edinburgh Parallel Computing Centre, University of Edinburgh, Edinburgh, UK.

[7] Usher Institute, School of Medicine, University of Edinburgh, Edinburgh, UK.

[8] School of Health and Wellbeing, University of Glasgow, Glasgow, UK.

[9] Health Informatics Centre, School of Medicine, University of Dundee, Dundee, UK.

[10] UK Dementia Research Institute Centre at the University of Edinburgh

[11] Cardiovascular Research, School of Medicine, University of Dundee, Dundee, UK.

[12] Health Data Research UK, London, UK

* equal contribution

** equal contribution

Correspondence: william.whiteley@ed.ac.uk

ABSTRACT

We present the design and implementation of a data curation framework to generate a large-scale clinical brain imaging dataset suitable for artificial intelligence (AI) enabled image analysis~~,~~. The dataset is accessible through the Brain Health Data (BHD) initiative~~. The raw data accessible through the BHD~~, which includes approximately ~~417K~~417,000 magnetic resonance imaging (MRI) and ~~846K~~846,000 computerized tomography (CT) head scans, linked electronic health records (EHRs), and associated free-text imaging reports from clinical practice between 2010 and 2018 in Scotland, ~~totally exceeding 185 TB storage of brain imaging and associated data.~~ exceeding 185 TB in size. The data curation framework was developed during the SCottish AI in Neuroimaging to predict Dementia and Neurodegenerative Disease (SCANDAN) study, which used a subset of 45,000 MRI from the BHD for dementia prediction.

We ~~present~~describe the ~~work curating~~processing of the ~~dataset~~BHD metadata and our multilabel classification output. We discuss the strengths of the BHD, including clinical relevance thanks to its unprecedented scale, population-wide representativeness of a national free-at-the-point-of-~~service~~delivery healthcare, long-term follow-up to neurodegenerative disease, and real-world variability. We ~~discuss~~describe the challenges and lessons learnt in developing ~~the~~a framework to curate ~~the~~ data ~~initially available~~, including the time needed to obtain ~~relevant~~ permissions, the need for easily accessible, secure, responsive and affordable computational environments, the variability ~~and inconsistencies~~ of clinical data ~~and records~~, and the challenge of extracting linked clinical data and images at scale~~, among others.~~ .

This resource will be crucial for clinical research, fostering the development of personalized medicine approaches, and fast-tracking the implementation of AI models in clinical workflows. We encourage the use of the BHD data through a streamlined application to the Public Benefit and Privacy Panel for Health and Care via the Data Research and Innovation Service ~~(eDRIS)~~ of Public Health Scotland (~~PHS~~eDRIS).

INTRODUCTION

Brain imaging plays a crucial role in the diagnosis of neurological disorders. However, clinical imaging services are under great demand, highlighting the need for new tools to improve radiology workflows. These tools should accelerate image assessment, reduce the workload for radiologists, and ultimately improve patient care. Artificial intelligence (AI) methods show promise for faster diagnosis (for example in acute ischaemic stroke)[1] and similar improvements are possible in head injury, neurodegeneration, dementia and brain cancers [2,3]. To develop and test AI models that are clinically relevant, researchers need access to large datasets of clinically acquired images, with sufficient computing resources, and secure, ethical data provision.

We present the Brain Health Data (BHD) initiative, a framework that unifies all clinical brain imaging data with related information collected in Scotland, to facilitate their access through the Electronic Data Research and Innovation Service (eDRIS) of Public Health Scotland (PHS) for other researchers. The BHD dataset includes magnetic resonance imaging (MRI) and computerized tomography (CT) head scans, linked electronic health records (EHRs), and free text radiology reports. We also present the design and implementation of the MRI data processing framework to curate this large-scale clinical brain imaging dataset in a format suitable for AI analysis, which we make available through the BHD. The work was undertaken during the SCANDAN project (SCottish AI in Neuroimaging to predict Dementia and Neurodegenerative Disease), which aimed to develop AI algorithms for reliable dementia risk estimation with routine brain imaging and clinical records. The SCANDAN work packages are illustrated in Figure 1. SCANDAN used Scotland-wide clinical practice data from 2010 to 2018 from more than 830 thousand individuals, now made accessible through the BHD. The BHD dataset offers clinical relevance with its unprecedented scale, population-wide representativeness of healthcare, long-term follow-up to neurodegenerative disease, and real-world variability.

A survey conducted between December 2024 and February 2025 across UK secure data environments found no comparablerevealed the lack of brain imaging resourceresources with nationwide coverage. For instance, the Diagnostic Imaging Dataset curated by NHS England includes patient-level metadata on the 501 million diagnostic imaging procedures performed withinin NHS England facilities since April 2012, but it lacks actual imaging data and associated reports. [4]

The preparation of large repositories of routinely collected imaging data is challenging, particularly withinin privacy-protecting secure data environments that are designed to protect patient privacy.. Despite adherence to Digital Imaging and Communications in Medicine (DICOM)[5] standards as best practice, real-world medical imaging datasets vary significantly in quality, format, and acquisition protocols, which makes standardizationstandardisation across different imaging sources necessary. Determining imaging sequences (e.g., T1- or T2-weighted magnetic resonance imaging (MRI))) is essential for analysis, but can be difficult in practice. DICOM meta-data tags provide rapid but sometimes unreliable classification, while image-based classification is more accurate but computationally demanding, and not free from

ambiguities.uncertainty. [6,7] Natural language processing (NLP) of radiology reports can aid infacilitate sequence identification if recorded in the free textand filter out scans with artefacts or missing structures. However, automated image quality assessment is paramount. Pre-processing and data retrieval is easier with automation of pipelines and integration of structured clinical records.

Filtering out unusable scans, such as those with artefacts or missing brain structures, is a critical step. Automated AI-based image quality assessment can help, but manual verification may be necessary. The automation of data pipelines, and the integration of structured clinical records with imaging data, can facilitate data retrieval and pre-processing. Compliance with governance frameworks is important to access large-scale unconsented clinical imaging datasets within safe havens, and needs ethical approval, data governance agreementsapproval, and compliance with privacy regulations, all with costs. These administrative barriers, although necessary, can significantly delay or completely deter research. To address these challenges, SCANDAN developed an automated framework that extracted, cleaned and prepared structured brain imaging and clinical data for AI analysis, creating a research-ready dataset. This paper presents SCANDAN's methods

To address these challenges, we developed the Brain Health Data (BHD) framework, which unifies all clinical brain imaging data acquired in Scotland with linked clinical information, to facilitate access to data through the Electronic Data Research and Innovation Service (eDRIS) of Public Health Scotland (PHS).[8] The data within the BHD framework includes approximately 417,000 MRI and 846,000 computerized tomography (CT) head scans, linked electronic health records (EHRs), and free text radiology reports collected between 2010 and 2018. It offers clinical relevance with its unprecedented scale, population-wide representativeness of healthcare, long-term follow-up to neurodegenerative disease, and real-world variability. To curate this large-scale clinical brain imaging dataset in a format suitable for AI analysis we designed and implemented a data processing pipeline within the SCottish AI in Neuroimaging to predict Dementia and Neurodegenerative Disease (SCANDAN) study, which aimed to develop AI algorithms for reliable dementia risk estimation from routine brain imaging and clinical records. This paper describes SCANDAN's methods and outputs, highlights key lessons learned from working within Scotland's data governance frameworks, and describes how to access the data through the BHD.

METHODS

Data sources

PHS' eDRIS provided brain CT and MR head studies in adults performed in Scotland between 2010 and 2018 from the Scottish Medical Imaging (SMI) service [6]. Study refers here to a complete imaging session, encompassing all images obtained during a single scanning session. Each scan contains three hierarchical levels: study, series, and images. Within each study, there are one or more series that group together images acquired using the same imaging technique or settings. Each series is, in turn, composed of multiple single images or "slices". We linked each study (i.e., one per person) deterministically with pseudonymized identifiers based on the

Community Health Index (CHI) number which is the unique patient identifier used across NHS Scotland. We linked them with hospital admission records (SMR01), dementia records from mental health hospitalisations (SMR04), dispensed prescriptions from Prescribing Information System (PIS), death records (National Records of Scotland (NRS)) and demographics (birth year, sex, deprivation index) since the year 2000. All the data was processed and stored within the National Safe Haven (NSH) a secure data environment provisioned by the Edinburgh Parallel Computing Centre (EPCC) (https://edinburgh-international-data-facility.ed.ac.uk/services/safe-haven-services/scottish-national-safe-haven).

Permissions and research governance

ForThe SCANDAN, study obtained multicentre research ethics permission from the NHS Human Research Authority was obtained from the North of Scotland Research Ethics Committee (23/NS/0017). Permission to access the data for SCANDAN and the pilot phase for the BHD was provided by the NHS Scotland Public Benefit and Privacy Panel for Health and Social Care (HSC-PBPP), a patient advocacy panel which scrutinises applications for access to NHS Scotland health data for non-direct care (PBPP applicationapplications 2223-0200 and 2223-0005). The respectively). [9] During the application process and throughout the study, the SCANDAN team engaged with several Scottish public and patient groups during the application process and throughout the project.

Computing environment

The Scottish National Safe Heaven (NSH), commissioned by PHS, where all processing is done, is hosted in EPCC'sEdinburgh Parallel Computing Centre's (EPCC) Trusted Research Environment (TRE), a secure infrastructure which currently hosts twelve Safe Havens. Each Safe Haven is operated under the "Five Safes" framework,[7] designed by the Office for National Statistics, [10] and the Scottish Government Charter for Safe Havens.[8][11] Researchers access a secure data sharing and analysis environment with a virtual desktop, under the terms and conditions prescribed by the data providerscontrollers. Standard software packages such as R and Python are available in the NSH; additional software packages can be installed from repositories such as the comprehensive R archive network (CRAN) and the Python package index (PyPI). Safe Havens have access to large shared-memory, high-performance computer clusters, including one with graphical processing unit (GPU) accelerators for large-scale analysis. For example, SCANDAN was provided a virtual environment with a GPU (NVIDIA A100 40GB), large storage (several TB) and RAM (100GB). All EPCC Safe Haven Services are operated at EPCC's Advanced Computing Facility, located in Edinburgh, Scotland. The EPCC Trusted Research Environment (TRE) is accredited by ISO27001 [9][12] for information security practices and self-certified under Cyber Essentials and NHS Digital's Data Security and Protection Toolkit (DSPT). In addition, the NSH is accredited under the Digital Economy Act 2017 by the UK Statistics Authority, and all Safe Havens in the TRE are operated to the same standard.

Data sources

PHS' eDRIS provided brain CT and MRI head studies in adults performed in Scotland between 2010 and 2018 from the Scottish Medical Imaging (SMI) service [13]. Study refers here to a complete imaging session, encompassing all images obtained during a single scanning session. Each scan contains three hierarchical levels: study, series, and images. Within each study, there are one or more series that group together images acquired using the same imaging technique and settings. Each series is, in turn, composed of multiple single two-dimensional images or "slices". A patient may have had multiple independent studies. Additionally, imaging reports are associated with studies and contain textual information regarding the imaging process and clinical interpretation of images. eDRIS and EPCC linked studies to patients deterministically with pseudonymized identifiers based on the Community Health Index (CHI) number, which is the unique patient identifier used across NHS Scotland. We linked them with outpatient records (SMR00), hospital admission records (SMR01), dementia records from mental health hospitalisations (SMR04), cancer registry (SMR06), community dispensed prescriptions from Prescribing Information System (PIS), death records (National Records of Scotland (NRS) and demographics (birth year, sex, deprivation index) since the year 2000. All the data was processed and stored within the NSH.

Data availability

The data on 830,000 patients was provided to SCANDAN and is available through the BHD. It includes 417,000 MRI studies, 846,000 CT studies and 1.8 million radiological reports. The studies contain 3.37 million MRI series and 3.15 million CT series. Figure 5 shows the distribution of slices per series for both CT and MRI for which the metadata was available at the start of the project. There were 356 million events from EHR, divided between the outpatient emergency and inpatient records (38 million), death records (327,000), prescription records (312 million) and accident and emergency records (4.5 million). For the 409 million DICOM slices available (MRI and CT), DICOM metadata were limited by the governance approval, with each tag being subject to approval. Consequently, the accepted metadata were provided separately in CSV format.

SCANDAN Project

The SCANDAN sequential work packages (WP) are illustrated in Figure 1. The NLP WP identified MRI sequences, CT type and brain pathologies from the radiological reports. The dementia labelling WP phenotyped dementia with EHRs. The cohort building WP selected a subset of MRI studies to carry out dementia classification. The image cataloguing WP labelled DICOM series with body part imaged, sequence, and presence of contrast and then filtered out non-desired scans based on the label. Images were then processed for AI analysis.

SCANDAN: Natural language processing of brain imaging reports

We applied a clinical NLP tool, the Edinburgh Information Extraction for Radiology (EdIE-R), ~~[10,11]~~[14,15] which was originally developed and validated for radiology reports of brain imaging in the Edinburgh Stroke Study and NHS Tayside ~~[12]~~[16]. EdIE-R processes radiology reports through a pipeline ~~architecture~~ that identifies entities, detects negation, extracts

relationships and assigns document-level labels to identify phenotypes~~ referring to brain abnormalities.~~. The tool was later adapted and validated for use with data from other areas in Scotland ~~.~~[13~~]~~]. EdIE-R ~~was used to~~can extract 24 distinct phenotypes, including different stroke types~~ of strokes~~ (ischaemic, haemorrhagic and underspecified, with time and location details), brain tumours (meningiomas, gliomas, metastases or underspecified), small vessel disease, microbleeds, atrophy and other ~~neurological~~ abnormalities. Additionally, it marked up MRI sequence types (T1, T2, and FLAIR~~) to support further analysis.~~).

To improve data selection, EdIE-R was enhanced to identify scans of non-head and non-brain body parts, and flag them for exclusion. ~~This ensured that only relevant imaging~~We improved the tool's ability to identify where distinct sections begin and end within reports~~ were included in the analysis. Enhancement of~~, such as the ~~detection of section boundaries allowed more precise separation of~~ boundary between the clinical history ~~from~~preamble and the main report ~~text so that~~body, enabling us to extract phenotype mentions ~~were only extracted~~exclusively from the ~~latter~~relevant report text.

EdIE-R contains several processing components. After pre-processing and linguistic analysis (e.g. tokenisation, sentence detection, lemmatisation and part-of-speech tagging) of the text in the input radiology report, EdIE-R performs named entity recognition, negation detection and relation extraction before conducting document-level classification of the 24 phenotypes. The output is the radiology report and its accompanying metadata as well as the information identified by EdIE-R represented in XML format which was then converted to CSV for follow-on analysis. It is keyed by the study identifier and does not contain information about a specific series.

The refined EdIE-R pipeline [15] was applied to all radiology reports in the SCANDAN project, producing structured outputs to guide data selection for image analysis~~ and predictive modelling.~~. By processing radiology reports within the Scottish NSH, the tool allows exclusion of scans (e.g., those showing tumours or non-brain regions) and served to validate outputs from imaging type classification and phenotype extraction. ~~This process streamlined the subsequent image analysis and predictive modelling processes.~~

Structured report DICOM contains TextValue elements of various kinds. Some are clinical reports, others contain non-clinical information. We aimed to select the best clinical report in each study for NLP processing. Not all study directories contained structured reports, and some contained more than one. For the latter, the process of choosing a report was as follows. From inspection of examples, it appeared that DICOM files containing real reports normally contained exactly one TextValue element. If a study contained one or more such DICOM files, we used the largest of those. In the cases where no report contained exactly one TextValue element, we just used the largest DICOM file, and processed the first TextValue element. If this was not a clinical report it would usually be marked as "empty" or "nocontent" by the NLP pipeline. Some reports were withheld because they were potentially identifiable, which accounts for studies with no report or no real report.

SCANDAN: Phenotyping dementia

~~We defined~~ We follow the phenotype specification for dementia ~~with~~ [18] based on prior studies in Scottish EHRs [19]. Dementia was defined as the presence of relevant ICD-10 codes ~~or prescribed medicines that are generally used for~~ , and, for the specific case of Alzheimer’s disease (AD~~) [14].~~), the prescription of AD medications. Each patient interaction with the health system, taken from the EHR, was used~~,~~ including a single stay in hospital, multiple consecutive stays, a prescription, or a death record. We defined labels for ‘any dementia’ and five dementia subtypes: AD, vascular dementia~~,~~ (VaD), other rare dementias, unspecified dementia and possible dementia. The subtype was defined as the most frequently occurring dementia phenotype in each person’s electronic record. ~~(Table 1).~~(Table 1). All individuals with a dementia label were categorised as cases, and individuals with no mention of dementia in any record were considered controls.

SCANDAN: Cohort building

~~To test the BHD data capability, we~~We built a matched case-control study cohort with ~~MR~~MRI brain images. A matched case-control design was chosen for several reasons. First, most deep learning and other algorithms work best with balanced cases and controls. Second, we had limited computing capacity at the beginning of the project.

Third, the rate of image delivery was limited by the need to copy data from a preparation area to a research area which had limited storage capacity. We ~~first~~ selected ~~useable~~ images ~~from those that had gone through the data pipeline to define image sequence, ensure presence of whole brain, absence of other body parts and absence of intravenous contrast, and had~~with no NLP label of tumour or haemorrhagic stroke in the radiology report~~. We selected people~~ from patients who were aged over 40 years at the time of scan ~~or~~and had an associated EHR record. ~~To exclude people with a~~We excluded dementia ~~diagnosis at time of scan, we defined controls as those~~ cases without ~~a diagnosis of dementia at any point in their record, and cases where a diagnosis of dementia was made~~scans taken more than one year ~~or more after their first scan~~before the time of diagnosis. For each individual, the first study chronologically was chosen. Dementia cases were matched to controls based on age at the time of scan (within one year of the matched case) and recorded sex from the linked demographic information. Cases with no matched controls were discarded. Age and sex matching was verified by analysing the resulting distributions over the entire cohort. A table was generated containing the identifier of the selected patient, their match, the selected study, demographic information, and the dementia ground truth.

~~Identification of MR scan type and sequence~~

~~DICOM tags were used to produce five labels for each image series: imaging sequence, presence of brain, presence of other body part, angiography, and imaging with contrast. We aimed to retain MRI series with sequences T1, T2 and Fluid-Attenuated Inversion Recovery (FLAIR), containing a brain and no other body part than the neck, without angiography or contrast, and with a 3D image volume of over 5 litres. These labels were subsequently combined with the results from the NLP tool, the MRI acquisition parameters, and the computed volume of the image series, to exclude those which did not meet SCANDAN criteria.~~

To produce these labels, the DICOM tags were parsed with regular expressions. For example, the expressions /(?i)(?<!pa)t2/ (case insensitive and ignoring occurrences starting with “pa”) and /*se2d1/ were associated with the intermediate label "tmp-T2" (T2-weighted), while the expressions /TOF/ and /MRA/ were associated with the intermediate label "tmp-MRA" (MR angiography). Then, the final labels were created by grouping all the intermediate labels of a series. For example, for an image series to be labelled "T1", it had to match the intermediate label "tmp-T1", and could optionally match the intermediate labels "FLAIR", "GRE" (gradient echo), and “FAT SAT” (fat saturation), which are not T1-weighted exclusive, but not any other intermediate label.

For sequence identification, the DICOM tag 'Series Description' (0008,103E) was used. To identify the body part, we used the tags 'Body Part Examined' (0018,0015), 'Protocol Name' (0018,1030), 'Performed Procedure Step Description' (0040,0254) and ‘Study Description' (0008,1030). Angiograms were identified with the tags ‘Angio Flag’ (0018, 00), ‘Study Description' (0008,1030), 'Protocol Name' (0018,1030) and ‘Series Description' (0008,103E). Contrast identification used ‘Study Description' (0008,1030), ‘Contrast/Bolus Agent’ (0018,0010), ‘Contrast/Bolus Route’ (0018,1040), 'Performed Procedure Step Description' (0040,0254) and 'Series Description' (0008,103E).

The results from the NLP tool provided additional information for the sequence identification and the presence of other body parts. Finally, the MRI sequence was defined with the tags ‘Echo Time' (0018,0081), 'Inversion Time' (0018,0082), 'Repetition Time' (0018,0080), 'Scanning Sequence' (0018,0020), 'Flip Angle' (0018,1314), 'Sequence Name' (0018,0024) based on "optimal" value [7-8] adapted to the data through observation on manually annotated data, to complement the identification based on the series description.

SCANDAN: Data validation and quality control

To generate ground truth labels for an initial evaluation of the automatic labelling process described abovein the next section, we developed a custom python-based graphical user interface (GUI) optimized for MRI and CT DICOM files. The GUI allowed users to load DICOM images from a single or nested folder structure. It utilized DICOM header metadata to: stack slices according to the acquisition order using the DICOM tag ‘Instance Number‘ (0020,0013), in ascending, descending, or interleave format, constructing and saving the 3D volumes for subsequent analysis; filter CT scans using the tag ‘Modality’ (0008,0060) and adjust their intensities, e.g. brain-windowing, using the tag ‘Rescale Intercept’ (0028,1052); determine the orientation of the imaging planes (i.e., axial, sagittal, or coronal) using the tag ‘Image Orientation (Patient)’ (0020,0037) to display mid-axial, mid-coronal, and mid-sagittal views for assessment; and 4c alculatecalculate the aspect ratio using the ‘Slice Thickness’ (0018,0050) and ‘Pixel Spacing’ (0028,0030) tags for accurate scaling and visualising of mid-view slices within the designated display area.

FiveRandomly sampled example scans (1,000) were selected prior to the large data delivery without stratification for image review. Among those, 287 were excluded for potential disclosive information. The remaining 390 MRI and 323 CT were annotated by five experts (3 clinicians

and 2 trained imaging scientists~~) annotated 713 randomly selected scans (388 MRI, 319 CT~~) with modality, sequence, presence of contrast, lesion and artefacts, presence of full brain, and presence of body parts (Figure 2). The 713 scans were evenly divided among the five annotators, with a subset of 100 images overlapping for cross-validation. The Generative model of Labels, Abilities, and Difficulties (GLAD) [14] probabilistic framework was used to estimate the true label for each image while accounting for annotator expertise and image difficulty, by using the overlapping 100 annotations as "truth" and correcting the rest of the annotations.

The ground-truth labels were compared with ~~those~~the labels obtained from the automatic labelling~~ using GLAD~~. Scans sequence type and contrast presence were further reviewed by a neuroradiologist and an experienced imaging scientist independently ~~where~~for the ~~ground truth and~~disagreement with the automatic labels~~ disagreed regarding sequence type and contrast~~, or ~~the ground-truth labels were~~for previous “unknown~~"~~” or ~~"~~“uncertain~~".~~” annotations. A third round of annotations resolved disagreements between the neuroradiologist and the imaging ~~scientists regarding the sequence type.~~scientist. The presence of brain, of other body part and the fullness of the brain was re-annotated by a trained image scientist~~, due to the simpler amount of 'unknown' result~~ as an independent re-annotation. After re-annotation, the ground-truth was updated using the most recently agreed version. We report the final comparison of the automatic tools and the manual annotations.

SCANDAN: Identification of MRI scan type and sequence

DICOM tags were used to produce five labels for each image series: imaging sequence, presence of brain, presence of other body part, angiography, and imaging with contrast. We aimed to retain MRI series with sequences T1, T2 and Fluid-Attenuated Inversion Recovery (FLAIR), that contained a brain and no other body part than the neck, without angiography or contrast, and with a 3D image volume of over 5 litres. The volume was empirically determined on another study using Scottish medical data, compared to the manual annotations, and visually asserted to separate two different normal distribution of scans volume.[20] These labels were subsequently combined with the results from the NLP tool, the MRI acquisition parameters, and the computed volume of the image series, to exclude those which did not meet SCANDAN criteria. Figure 4 illustrates the methods and data sources for the labelling and exclusion of images.

To produce these labels, the DICOM tags were parsed with regular expressions.[21] For example, the expressions /(?i)(?<!pa)t2/ (case insensitive and ignoring occurrences starting with “pa”) and /*se2d1/ were associated with the intermediate label "tmp-T2" (T2-weighted), while the expressions /TOF/ and /MRA/ were associated with the intermediate label "tmp-MRA" (MR angiography). Then, the final labels were created by grouping all the intermediate labels of a series. For example, for an image series to be labelled "T1", it had to match the intermediate label "tmp-T1", and could optionally match the intermediate labels "FLAIR", "GRE" (gradient echo), and “FAT SAT” (fat saturation), which are not T1-weighted exclusive, but no any other intermediate label.

For sequence identification, the DICOM tag 'Series Description' (0008,103E) was used. To identify the body part, we used the tags 'Body Part Examined' (0018,0015), 'Protocol Name' (0018,1030), 'Performed Procedure Step Description' (0040,0254) and ‘Study Description'

(0008,1030). Angiograms were identified with the tags 'Angio Flag' (0018, 00), 'Study Description' (0008,1030), 'Protocol Name' (0018,1030) and 'Series Description' (0008,103E). Contrast identification used 'Study Description' (0008,1030), 'Contrast/Bolus Agent' (0018,0010), 'Contrast/Bolus Route' (0018,1040), 'Performed Procedure Step Description' (0040,0254) and 'Series Description' (0008,103E).

The results from the NLP tool provided additional information for the sequence identification and the presence of other body parts, validating the presence of a sequence within a study. Finally, to complement the identification based on the series description the MRI sequence was also defined with the tags 'Echo Time' (0018,0081), 'Inversion Time' (0018,0082), 'Repetition Time' (0018,0080), 'Scanning Sequence' (0018,0020), 'Flip Angle' (0018,1314), 'Sequence Name' (0018,0024) based on "optimal" value [7-8] adapted to the data through observation on manually annotated data.

Each regular expression rule was based on prior research carried on in-house clinical studies. They were expanded to ignore conflict and formatting due to the greater distribution of value from the 35 hospitals which the data originates from. The most common occurrence for each DICOM tag were compared to their label using the metadata of the entire cohort, to ensure the rules were not including unwanted samples. Finally, they were refined to agree with the manual annotation during each re-annotation.

RESULTS

SCANDAN: Output Dataset ~~description~~

~~MR and CT brain images were available for 830,884 people. Exclusion criteria at subject levels and their effects are listed~~Taking the first chronological study in ~~Table 3.~~each sequence of studies (MR or CT) for each person gave 1.1 million studies of which 311,000 were MRI and 789,000 CT. Among these, 16,000 MRI and 119,000 CT were associated with a record of dementia. After applying exclusion criteria, as described in Table 3, 10,709 MRI and 57,242 CT dementia cases were age and sex matched ~~to~~with the same number of healthy controls. ~~The earliest 21,418~~We eliminated 1,171 MRI and ~~114,484 CT studies associated with these subjects were~~3,302 CT dementia cases with a text report containing a mention of tumour or haemorrhagic stroke; 70 MRI and 100 CT dementia cases due to the patient being under 40 at the time of scans; and 4,869 MRI and 58,779 CT cases because the scans occurred within one year prior to the dementia diagnosis.

Of the 1,481,643 study directories, 449,369 had no structured report, 655,450 study directories contained exactly one report and 376,824 study directories contained more than one report. Each selected report was processed using the EdIE-R NLP pipeline. Across the full dataset, the most frequently detected phenotypes were small vessel disease (25.0% of studies) and atrophy (23.2%), reflecting their high prevalence in an ageing clinical population. Ischaemic stroke findings were also common (e.g. old deep ischaemic stroke was detected in 9.4% of studies), while haemorrhagic stroke subtypes were comparatively rare (0.2–2.9%). Tumour-related findings were detected in 0.4–4.1% of studies, depending on subtype. Regarding imaging sequences, T2 was the most frequently recorded (13.4% of studies), followed by T1 (8.3%) and

FLAIR (7.8%). These NLP-derived labels formed the basis for the phenotypic exclusion criteria applied to the dementia cohort.

Of the 21,418 MRI scans requested, ~~of which~~ 21,197 ~~and 94,740~~ were successfully ~~retrieved.~~ received, with 221 being excluded for privacy reasons. These studies contained 128,257 series of which 73,457 were identified as T1, T2 or FLAIR, 18,681 series were localisers, 4,372 as unknown, 30,267 were other MRI sequences (DWI, SWI, T2* etc.), and 1,464 series had a series description that did not differentiate T1 and T2*. Table 5 describes the filtering process making use of the DICOM labelling process, which resulted in the exclusion of 2,641 FLAIR, 11,820 T1 and 10,863 T2. After restricting the selection to the first chronological series for each study, 41,966 series were kept from 15,558 studies. Later, 277 studies were excluded when they failed to convert to NIfTI and subsequent process.

The MRI studies requested originated from 35 hospitals across Scotland using 27 unique MRI scanners models were identified (14 models with <100 studies), with 60% of the studies using a Siemens model, 20% General Electric (GE) and 20% Philips, and a handful from 2 other manufacturers. 94% of the scans were done with a 1.5 Tesla, and 6% a 3 Tesla MRI scanner.

The MRI cohort contained 8,145 cases (53.2% female) and 7,236 controls (54.1% female) (Table 4). The mean age at scan was 74 years. The mean time from scan to first mention of dementia was 5 years for cases, and the mean follow-up time for controls was 6 years 9 months. Of the 8,145 dementia cases there was non-exclusive record of AD in 3,774, vascular dementia in 3,386, unspecified dementia in 3,784 and other dementia types in 508. The mean number of hospitalisations in the year prior to scan was 1.1 (standard deviation ([SD] 1.52) for cases and 1.0 (SD 1.50) for controls. During the same period, the mean number of prescriptions was 15.4 for cases and 14.2 for controls.

SCANDAN: Data validation and quality control

For simplicity, we refer to the results of the manual annotations as "annotations", and the results of the automatic tools described in Figure 4 which are compared to the manual annotations as "labels".

During the first round, 707 annotations were obtained from 713 images. Four images could not be read due to acquisition errors. Two images were only partially annotated due to visual perception ~~error~~errors and discarded. In 29 (4.5%) ~~images were~~ cases, image modality was wrongly annotated because ~~several~~the scans ~~containing~~contained only one slice (i.e. a localiser) or did not ~~containing~~contain a brain. ~~As the annotators were less familiar with CT~~Of CTs, 24 (7.52%) ~~of CT images~~ were ~~written~~classed as 'Unknown'. In the labelling process we used the previously validated DICOM tag 'Modality' (0008,0060~~),~~) to identify CT and MRI scans. The identification of the body parts was easier for the annotators than the modality or sequence type. “Unknown” was given for 23 (3.~~25~~3%) series when annotators were questioned whether they contained a brain or not, 40 (5.~~66~~7%) when questioned if the brain was acquired in full, and in 36 (5.~~09~~1%) series the annotators could not assert whether there was another body part. Annotators could not identify the sequence type for 85 (12.~~02~~0%) series and the presence of contrast in 104 (14.~~71~~7%) series.

In this first round of annotations, the main ~~source of~~ disagreements between annotations and labels ~~was~~were the presence of non-brain images or localisers. ~~For example, non-brain scans had a higher rate of mistakes in sequence annotation.~~ For further analyses, the scans labelled as 'localiser' in the first round of annotations, ~~which contained under~~defined as series with less than 15 slices, were ignored. If the annotation and label agreed on the absence of brain, the images were not re-annotated.

In the second round of annotations, ~~the~~ images with an unknown sequence type (16 scans), and those with disagreement between label and annotation ~~in which either assigned~~ ('T1', 'T2' or 'FLAIR' ~~(~~, 48 scans~~)~~), were re-annotated. Additionally, a subset of images was selected ~~out of~~from 66 ~~which had~~with partial agreement between at least one sequence label and the annotation, to validate commonly occurring combination of labels which were not similar ~~but not outright disagreeing~~ (e.g. 'T1' + 'GRE' instead of 'T1' + 'T1'). Scans which contained at least one mention of 'T1', 'T2' or 'FLAIR' in either the annotation or the label were re-annotated for presence of contrast ~~presence~~ when disagreement was found or when they were annotated as 'Unknown'. Series with 'Unknown' annotation for questions regarding brain presence (13 scans), other body part presence (8 scans) and whole brain (11 scans) were also re-annotated. Disagreement between the annotation and the labels were also re-annotated, respectively 7, 143 and 20 scans. In case of a whole brain, the disagreement was ignored if other body parts were present ~~and if~~in both the label and the annotation ~~agreed~~. In total, 143 series were re-annotated for the presence of brain and other body parts, and for full brain coverage. Additionally, there were 84 images re-annotated for sequence type and contrast.

To resolve conflict between the two re-annotators, or between them and the labelling tools, 27 series were then annotated a third time. Some conflicts could not be resolved, such as 7 images having the same 'Series Description' (0008,103E) tag value, and thus the same label. Three of them were identified as T1 and four as T2* by the two annotators in agreement.

Between each round of annotation, the regular expressions used by the labelling tools were updated to reflect previously unknown, and to solve conflicting information and errors.

The results of the labelling tools compared to the final annotation as ground truth were very good. The true positive rate ranged from 87% to 97% and the positive predictive value from 81% to 99% (Table 2). ~~The presence~~For consistency with our metrics, we evaluated the absence of contrast and other body ~~parts and contrast was amended to their absence, the target which we considered "~~part respectively as "positive~~".~~". This value excludes localisers for sequence type, and series without presence of brain for 'whole brains'. The lower precision for detection of other body parts is explained by the lack of mention of any parts in the different DICOM tags, sometimes due to missing data, as well as the detection of some other head parts, such as the jaw, without mention of the brain, which often, but not always, indicate non brain scans. The lower recall for the absence of contrast is caused by the low number of studies that used intravenous (IV) contrast. During a scanning session that used IV contrast, a first image will normally be captured free of contrast, prior to the injection, however, the 'Study Description' (0008,1030) will indicate the presence of IV contrast nonetheless for this first series, as was commonly found.

SCANDAN contribution and BHD data

The SCANDAN project produced data which were added to the BHD. In addition to the 1.2 million brain studies from 830, 000 patients, the 1.8 million radiological report and the 356 million EHR available as raw data, researchers can also access five additional tables: 1. summary of the valid radiological reports generated by the NLP; 2, dementia phenotyping table, with dementia subtype probability and date of diagnosis; 3. patient history, curated and listing all relevant information from all EHR; 4. the manual annotations of the 708 scans; 5. the labelling of the 21K MRI of the SCANDAN cohort for MRI sequence, body part imaged, brain fullness and contrast presence. The latter is planned to be expanded to the totality of MRI and then CT scans.

Permissions and governance

~~Our~~The SCANDAN application to the PBPP, which included an industry partner and aimed to develop an AI algorithm, required 210 days spanning 4 iterations for approval from the initial submission and over 17,000 words across 33 pages.

However, with the development of the BHD, researcher can now apply to PBPP to access these data with a shorter application and streamlined process. The data flow and linkage process for the BHD framework are schematically illustrated in Figure 3. Researchers can ~~either~~ log into a workspace running in the NSH~~, over approved secure channels, where~~ with data ~~will be provided directly alongside the~~ and tools to perform analysis. ~~-~~To run externally developed tools, they can build a container outside the NSH~~,~~ and pull it from a public registry ~~(such as the gihub container registry: ghcr.io). Alternatively, we plan to allow for the ADDI Workbench (https://www.alzheimersdata.org/ad-workbench) to run an analysis workflow externally, and receive the output once approved by eDRIS.~~after approval. It should be noted that no data leaves the ~~EPCC TRE~~NSH during this process. The TRE is divided in several zones (Figure 3). The blue zones, where eDRIS store the data, are not accessible to the researchers. ~~The different areas the researchers~~Researchers have access to ~~carry their work comprise the~~ green zones~~. They will have~~ with access to subsets of the data, as defined by their ~~project group. The zones coloured in yellow are external to the NSH and represents cloud resources, such as the ADDI workbench, or container registry.~~

~~There can be different project groups working simultaneously through the BHD. Each group may only have~~ access ~~to a subset of the data as necessary for their research, whether that be a subset of tables, a subset of rows, or a subset of patients. To gain an additional level of confidentiality each group will only see a CHI replaced by a pseudonymised identifier which is specific to their group. eDRIS will maintain a mapping from the Encrypted Universal Patient Identifier (EUPI) to these group-specific pseudonymised identifiers~~permission.

PHS eDRIS will prepare suitable subsets of the data for a particular research group and copy it to their working space. ~~The eDRIS research coordinator will review any~~Any results ~~of the researcher team to be released~~required outside ~~of~~ the ~~TRE, for example~~NSH, e.g., for publication~~.~~ , are subject to disclosure control performed by eDRIS.

DISCUSSION

To our best knowledge, BHD is the first large-scale, curated brain imaging clinical dataset relevant to dementia research that is available to researchers via moderated public access. The dataset offers several advantages in addition to its large size: clinical relevance, long-term follow-up, co-location with a GPU cluster in a safe haven, greater population representativeness compared to many research cohorts, and accessibility for clinical researchers. The resource continues to grow in data size and computing power.

Working with health systems data presents challenges. One of them is the time ~~elapsed~~taken for governance ~~approvals~~approval. In our case, if governance had been applied for after the funding had been awarded, it would have represented 58% of a 1-year postdoctoral award. This would not only impact negatively on career development of the post holder but also delay the project goals~~. To address this barrier, BHD has co-developed a streamlined application process with PHS, which included simplified forms for pre-competitive research and faster approval timelines.~~, an issue addressed by the streamlined process of the BHD Data provision was initially constrained by the limitations of the virtual machine environment, limited staff availability, and increased procedural complexity resulting in delayed access to imaging data and complicating project planning. The ~~framework established by SCANDAN, now adopted by PHS, and the~~ experience gained ~~throughout~~through the ~~conduction of the~~SCANDAN project, ~~are expected~~which piloted the access to ~~accelerate~~the data ~~delivery for~~, allowed PHS to streamline the governance process and improve the data provisioning to future projects. It is important however, to note that all research outputs generated within the NSH must undergo review by PHS staff prior to release.

Most imaging research is based on uniformly acquired research data. In contrast, clinical scans acquired in a routine free-at-the-point-of-service healthcare are sometimes incomplete, may be obscured by movement or other artefacts, show signs of non-relevant pathologies, may have been obtained with non-standardised protocols, and on different machines. However, such real-world data with inherent variability is essential for the development of software tools suitable for robust ~~application~~applications in clinical practice where such heterogeneity is the norm.

Using electronic health records for dementia diagnosis has limitations. Currently, primary care data are unavailable through PHS~~.~~ and thus cannot be provided by the BHD. Hence, we relied on recorded diagnosis after an inpatient stay or death. Hospital and death records under-ascertain (false negatives) dementia in the short term and have modest reliability for dementia subtypes ~~[15]~~[22]. However, they have also previously shown high positive predictive value for all dementia diagnosis [14]. Referral reasons for scans acquisitions are not currently available, although further NLP work with reports could achieve this.

The use of head scans does raise privacy concerns due to facial recognition risks. We have mitigated these by working only in a safe haven environment, ~~visually~~ examining only brain slices, prohibiting facial reconstruction, limiting access to approved researchers, who accept the restrictions and ~~having~~conditions of working in the NHS specified in the eDRIS User Agreement which includes PHS strictly checking all outputs from the secure environment, to exclude any identifiable data. Future work aims to further mitigate privacy risks by limiting the need for direct human access to data, for example by implementing software via containers. However, this

work needs training of the research community, better labelling of metadata (so the data is truly FAIR), and further development of technology within the NSH environment.

There are many opportunities for further linkage to other datasets (for example community retinal imaging~~[16]~~ [23]). Such work will require further engagement with public contributors, ~~and improvement in the security process~~use of federated analysis and federated learning with ongoing adoption of tools and techniques to ~~extract~~assess disclosure risks of different AI models~~ trained within the NSH~~.

~~Researchers can access the BHD data by applying to eDRIS.~~The SCANDAN project piloted the access to the data now provided through the BHD. While its primary goal was to establish a proof of concept for dementia classification using clinical data, it produced several secondary outputs which are now available to other researchers using the BHD data. As more projects will use the data, additional output will be added, compounding with time to an unvaluable resource for brain imaging research. Researchers can access the BHD data by applying to PBPP via eDRIS. Proposals must demonstrate a clear public benefit, and researcher-generated outputs must be added back to the dataset so every project strengthens the next. We strongly encourage cross-group collaboration. The resources available through the BHD are growing in terms of data availability, storage capacity, and computing power that are provided to researchers. We hope that this, and similar global initiatives, will ultimately contribute to improve the brain health of people worldwide.

FUNDING

This work was supported by NEURii, a collaborative partnership involving the University of Edinburgh, Gates Ventures, Eisai, LifeArc and Health Data Research UK (HDR UK). We acknowledge the eDRIS team (Public Health Scotland) for their support in obtaining approvals, the provisioning and linking of data and facilitating access to the National Safe Haven. The Brain Health Data Pilot is supported by Alzheimer’s Disease Data Initiative (ADDI) and HDR UK with funding to the University of Edinburgh.

CONFLICTS OF INTEREST

MVH and JMW are supported by Row Fogo Charitable Trust (Grant no. BRO-D.FID3668413). JMW was supported by the UK Dementia Research Institute (award no. UKDRI –4002 and 4205, DRIEdi17/18, and MRC MC_PC_17113) which receives its funding from DRI Ltd, funded by the UK Medical Research Council, Alzheimer’s Society and Alzheimer’s Research UK. ST acknowledges support of the UKRI AI programme, and the Engineering and Physical Sciences Research Council (EPSRC), for CHAI - Causality in Healthcare AI Hub [grant number EP/Y028856/1]. WW and HW are supported by HDRUK.

REFERENCES

1. Westwood M, Ramaekers B, Grimm S, Armstrong N, Wijnen B, Ahmadu C, et al.. Software with artificial intelligence-derived algorithms for analysing CT brain scans in people with a suspected acute stroke: a systematic review and cost-effectiveness analysis. *Health Technol Assess (Rockv)*. NIHR Journals Library; 2024; doi: 10.3310/RDPA1487.

2. Ferber D, El Nahhas OSM, Wölflein G, Wiest IC, Clusmann J, Leßmann ME, et al.. Development and validation of an autonomous artificial intelligence agent for clinical decision-making in oncology. *Nat Cancer*. Nature Research; 2025; doi: 10.1038/S43018-025-00991-6;SUBJMETA.

3. D'Adderio L, Bates DW. Transforming diagnosis through artificial intelligence. *NPJ Digit Med*. Nature Research; 2025; doi: 10.1038/S41746-025-01460-1.

4. . Classification: OfficialNHS England. Diagnostic Imaging Dataset Annual Statistical Release 2023/24. 2024 Nov.

5. Larobina M. Thirty Years of the DICOM Standard. *Tomography*. Multidisciplinary Digital Publishing Institute (MDPI); 2023; doi: 10.3390/TOMOGRAPHY9050145,.

6. Baxter R, Nind T, Sutherland J, McAllister G, Hardy D, Hume A, et al.. The Scottish Medical Imaging Archive: 57.3 Million Radiology Studies Linked to Their Medical Records. *Radiol Artif Intell*. Radiological Society of North America Inc.; 2024; doi: 10.1148/RYAI.220266/ASSET/IMAGES/LARGE/RYAI.220266.FIG2.JPEG.

Liang S, Beaton D, Arnott SR, Gee T, Zamyadi M, Bartha R, et al.. Magnetic Resonance Imaging Sequence Identification Using a Metadata Learning Approach. *Front Neuroinform*. Frontiers Media S.A.; 2021; doi: 10.3389/fninf.2021.622951.

7. de Mello JPV, Paixão TM, Berriel R, Reyes M, Badue C, de Souza AF, et al.. Deep learning-based type identification of volumetric MRI sequences. *Proceedings - International Conference on Pattern Recognition*. Institute of Electrical and Electronics Engineers Inc.; 2020; doi: 10.1109/ICPR48806.2021.9413120.

8. : What is eDRIS? - Overview - Electronic Data Research and Innovation Service (eDRIS) - Health intelligence and data management - Resources and tools - Public Health Scotland. https://publichealthscotland.scot/resources-and-tools/health-intelligence-and-data-management/electronic-data-research-and-innovation-service-edris/overview/what-is-edris/ Accessed 2026 Mar 27.

9. : Public Benefit and Privacy Panel for Health and Social Care. https://www.informationgovernance.scot.nhs.uk/pbpphsc/ Accessed 2026 Mar 27.

10. : The Five Safes Framework - GOV.UK. https://www.gov.uk/data-ethics-guidance/the-five-safes-framework Accessed 2025 May 28.

811. : Charter for Safe Havens in Scotland: Handling Unconsented Data from National Health Service Patient Records to Support Research and Statistics. - gov.scot.

https://www.gov.scot/publications/charter-safe-havens-scotland-handling-unconsented-data-national-health-service-patient-records-support-research-statistics/ Accessed 2025 May 28.

~~9~~12. : ISO/IEC 27001:2022~~–~~ Information security management systems. https://www.iso.org/standard/27001 Accessed 2025 May 28.

13. Baxter R, Nind T, Sutherland J, McAllister G, Hardy D, Hume A, et al.. The Scottish Medical Imaging Archive: 57.3 Million Radiology Studies Linked to Their Medical Records. *Radiol Artif Intell*. Radiological Society of North America Inc.; 2024; doi: 10.1148/RYAI.220266/ASSET/IMAGES/LARGE/RYAI.220266.FIG2.JPEG.

~~10~~14. Alex B, Grover C, Tobin R, Sudlow C, Mair G, Whiteley W. Text mining brain imaging reports. *J Biomed Semantics*. England; 2019; doi: 10.1186/s13326-019-0211-7.

~~11~~15. : Software – Language Technology Group. https://www.ltg.ed.ac.uk/software/ Accessed 2025 Aug 14.

~~12~~16. Wheater E, Mair G, Sudlow C, Alex B, Grover C, Whiteley W. A validated natural language processing algorithm for brain imaging phenotypes from radiology reports in UK electronic health records. *BMC Med Inform Decis Mak*. 2019; doi: 10.1186/s12911-019-0908-7.

~~13~~17. Casey A, Davidson E, Grover C, Tobin R, Grivas A, Zhang H, et al.. Understanding the performance and reliability of NLP tools: a comparison of four NLP tools predicting stroke phenotypes in radiology reports. *Front Digit Health*. Switzerland; 2023; doi: 10.3389/fdgth.2023.1184919.

~~14. Whitehill J, Wu T, Bergsma J, Movellan J, Ruvolo P. Whose Vote Should Count More: Optimal Integration of Labels from Labelers of Unknown Expertise. *Adv Neural Inf Process Syst*. 222009;~~

~~15~~18. : Phenotype Library | Phenotype: Dementia Identification with EMR. https://phenotypes.healthdatagateway.org/phenotypes/PH1717/version/3973/detail/ Accessed 2026 Mar 21.

19. Doney ASF, Bonney W, Jefferson E, Walesby KE, Bittern R, Trucco E, et al.. Investigating the Relationship Between Type 2 Diabetes and Dementia Using Electronic Medical Records in the GoDARTS Bioresource. *Diabetes Care*. American Diabetes Association; 2019; doi: 10.2337/dc19-0380.

20. Reel PS, Al-Wasity S, Edwards C, Reel S, Mansouri-Benssassi E, Suveges S, et al.. Machine learning-based prediction of future dementia using routine clinical MRI brain scans and healthcare data. *medRxiv*. Cold Spring Harbor Laboratory Press; 2025; doi: 10.1101/2025.11.12.25340070.

21. : GitHub - SCANDAN-Team/SCANDAN-DICOM-labelling: Rules for DICOM tag based labelling · GitHub. https://github.com/SCANDAN-Team/SCANDAN-DICOM-labelling Accessed 2026 Mar 30.

22. McGuinness LA, Warren-Gash C, Moorhouse LR, Thomas SL. The validity of dementia diagnoses in routinely collected electronic health records in the United Kingdom: A systematic review. *Pharmacoepidemiol Drug Saf*. John Wiley and Sons Ltd; 2019; doi: 10.1002/PDS.4669.

~~16~~23. Tochel C, Bernabeu MO, McTrusty A, Tatham AJ, Pead E, Buckmaster F, et al.. SCONe: a community-acquired retinal image repository enabling ocular, cardiovascular and neurodegenerative disease prediction. *BMJ Health Care Inform*. BMJ Publishing Group; 2025; doi: 10.1136/BMJHCI-2024-101236.

TABLES

Table 1: International classification of diseases 10 (ICD-10) and British national formulary (BNF) codes to define dementia subtypes. The definition of the codes follows the phenotyping employed previously.[18] * indicates a wild-card, meaning that all child-codes in the hierarchy are included.

Subtype	**ICD10**	**BNF**
Alzheimer's disease	F00* G30*	0411000
Vascular dementia	F01*	
Other rare dementias	F02*, G31.0, A81.0	
Unspecified dementia	F03, F05.1	
Possible dementia	F05.0, G31	

~~*Indicates wildcard~~

Table 2: ~~Labels from~~The metrics of the comparison between the automated ~~pipeline versus ground truth~~ labels and the manual annotation as ground truth for image characteristics.

**Algorithm identified image characteristics**	**Recall (%)**	**Precision (%)**
sequence type	94.1%	98.6%
study contains brain	97.1%	91.0%
absence of other body parts	93.1%	81.9%
whole brain in study	96.4%	95.6%
absence of contrast in study	87.3%	95.4%

Table 3: Selection process for the MRI and CT cohorts, showing both the overall and dementia counts, with addition of characteristics

**Criterion**	**MRI**		**CT**	
	Overall	Dementia	Overall	Dementia
**With scan**	294,422	16,819	669,539	119,423
**+ No reported tumour or haemorrhagic stroke**	280,549	15,648	640,400	116,121
**+ Hospitalised electronic health record**	279,004	15,648	638,680	116,121
**+ Diagnosis other than 'possible' dementia**	275,970	15,648	627,911	116,121
**+ Age at scan > 40**	207,876	15,578	523,855	116,021
**+ Dementia diagnosis or follow-up > 1 year**	190,582	10,709	391,356	57,242

MRI: magnetic resonance imaging of brain; CT: computerised tomography of brain

Table 4: Distribution of subjects with mention of each dementia type and, controls grouped by key characteristics

		Alzheimer's	Vascular	Other or rare	Unspecified	Controls
**Sex**	Female	2,057	1,681	207	2,047	3,917
	Male	1,717	1,705	301	1,737	3,319
**Age in years**	Mean (SD)	73 (8.8)	75 (8.7)	67 (9.3)	74 (8.9)	74 (9.0)
	40-50	36	32	19	38	87
	51-60	282	169	90	227	434
	61-70	968	706	179	813	1611
	71-80	1,694	1,503	182	1,664	3,182
	81+	794	976	38	1,042	1,922
**SIMD**	Mean (SD)	2.8 (1.3)	2.7 (1.3)	2.8 (1.3)	2.7 (1.3)	2.8 (1.3)
	1	719	768	102	842	1429
	2	732	650	102	724	1380
	3	753	635	94	703	1381
	4	909	691	113	780	1523
	5	363	242	42	325	804
**Hospitalisation 1 yr before scan, Mean (SD)**		0.9 (1.4)	1.2 (1.5)	1.0 (1.6)	1.1 (1.5)	1.0 (1.5)
**Prescriptions 1 yr before scan, Mean (SD)**		14.5 (9.5)	16.1 (0.2)	14.3 (9.7)	15.6 (10.2)	14.2 (9.4)

SD: standard deviation; SIMD: Scottish index of multiple deprivation

~~**Table 5**: Final population~~

Table 5: Selection process based on the automatic labelling for the MRI cohort. The “Original” column shows the amount of series for each sequence type. The "Selected" columns show the number of series kept after the selection process ready for analysis. Every column in between is associated with an excluding step and shows the number of series it excluded.

Sequence	Original	Brain	Whole Brain	Other Body Parts	Contrast & Angio	One Series per Study	~~Final~~Selected
FLAIR	16,871	-60	-211	-1,742	-628	-714	13,516
T1	27,627	-1,290	-4,670	-2,707	-3,153	-2,156	13,651
T2	28,959	-1,407	-4,828	-3,738	-890	-3,297	14,799

FIGURES

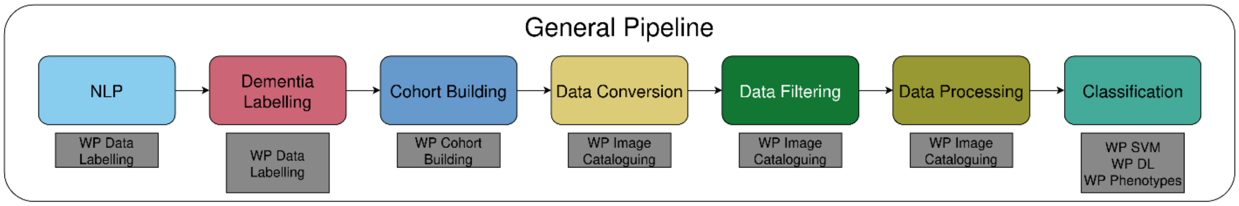

Figure 1: Work packages (WP) in the SCANDAN project: Data labelling, cohort building, image cataloguing, and processing for classification into being indicative of having dementia or not using deep learning (DL), support vector machine (SVM) and from the analysis of extracted imaging phenotypes.

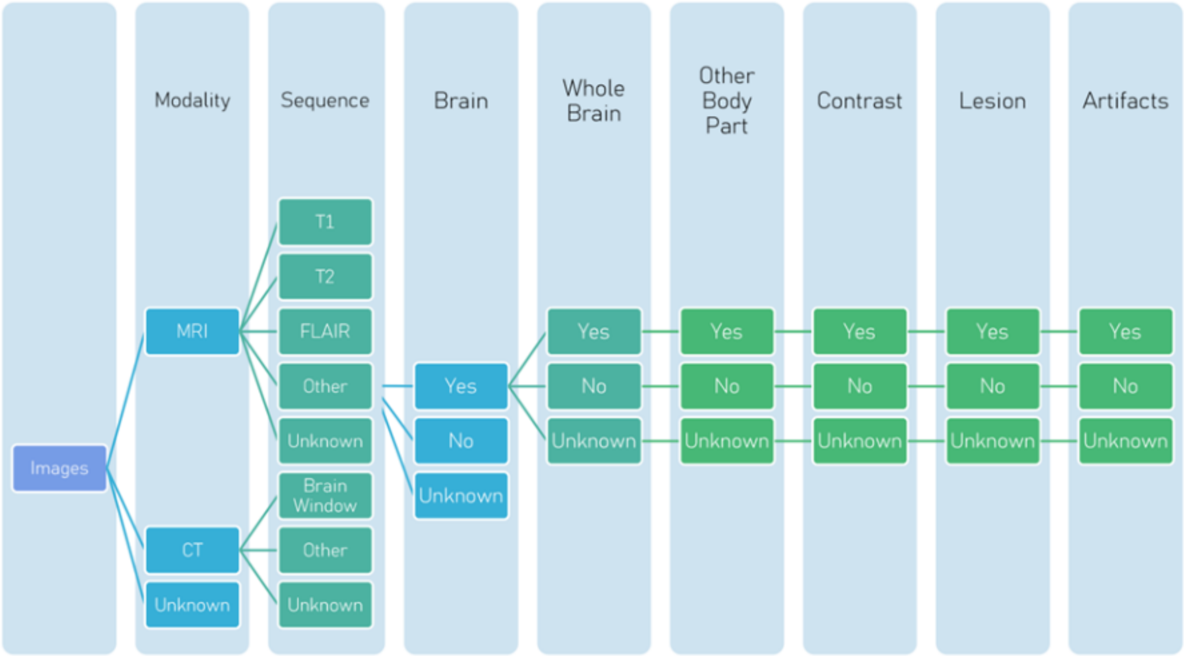

Figure 2: Criteria used by the annotators to label the test imaging set using the GUI developed.

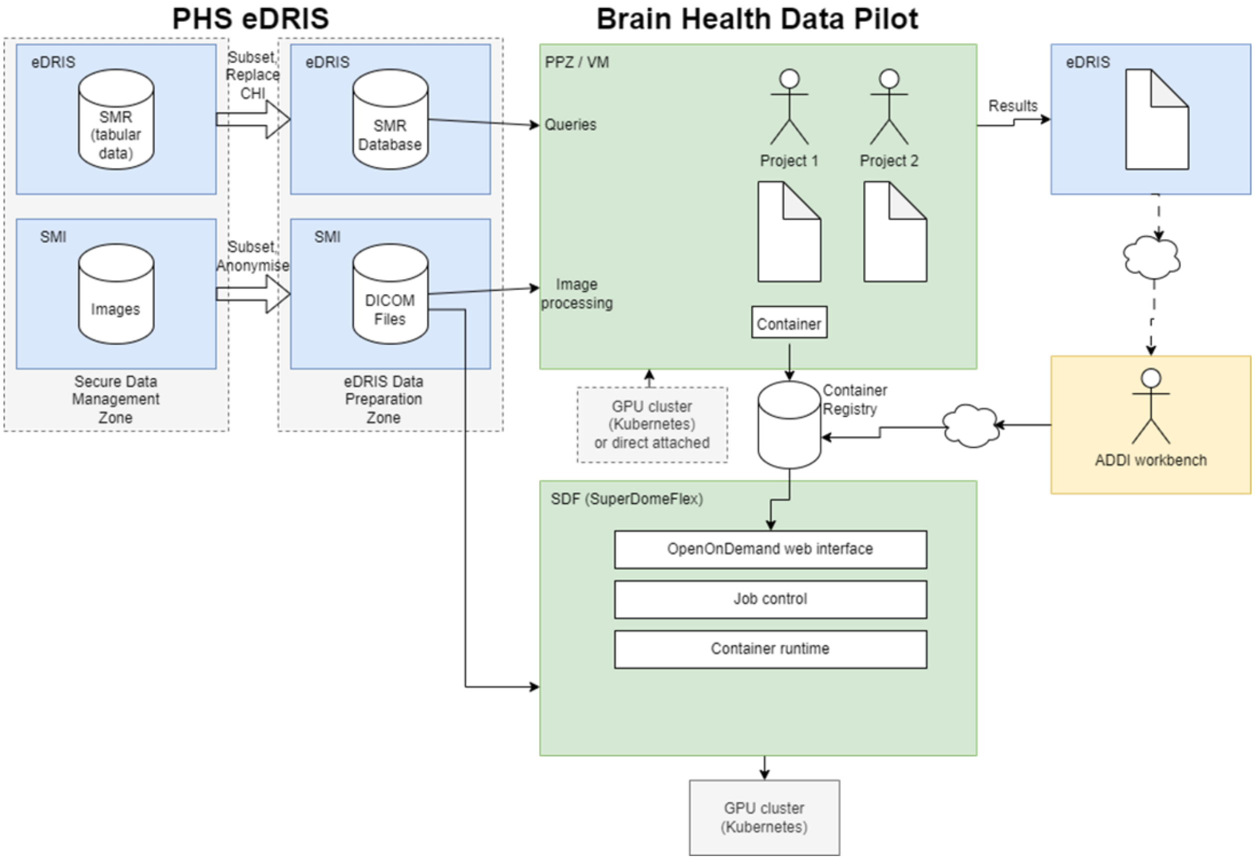

Figure 3: Diagram to illustrate the data flow and data linkage process in the ~~BHD.~~Brain Health Data service (BHD). eDRIS Electronic Data Research and Innovation Service (eDRIS), SMR: Scottish Morbidity Record, CHI Community Health Index number, PPZ: privacy preserving zone, VM: virtual machine, DICOM: Digital Imaging and Communications in Medicine

~~Figure 4. Flowchart summarizing the filtering of the MRI scans using DICOM metadata and structured report~~

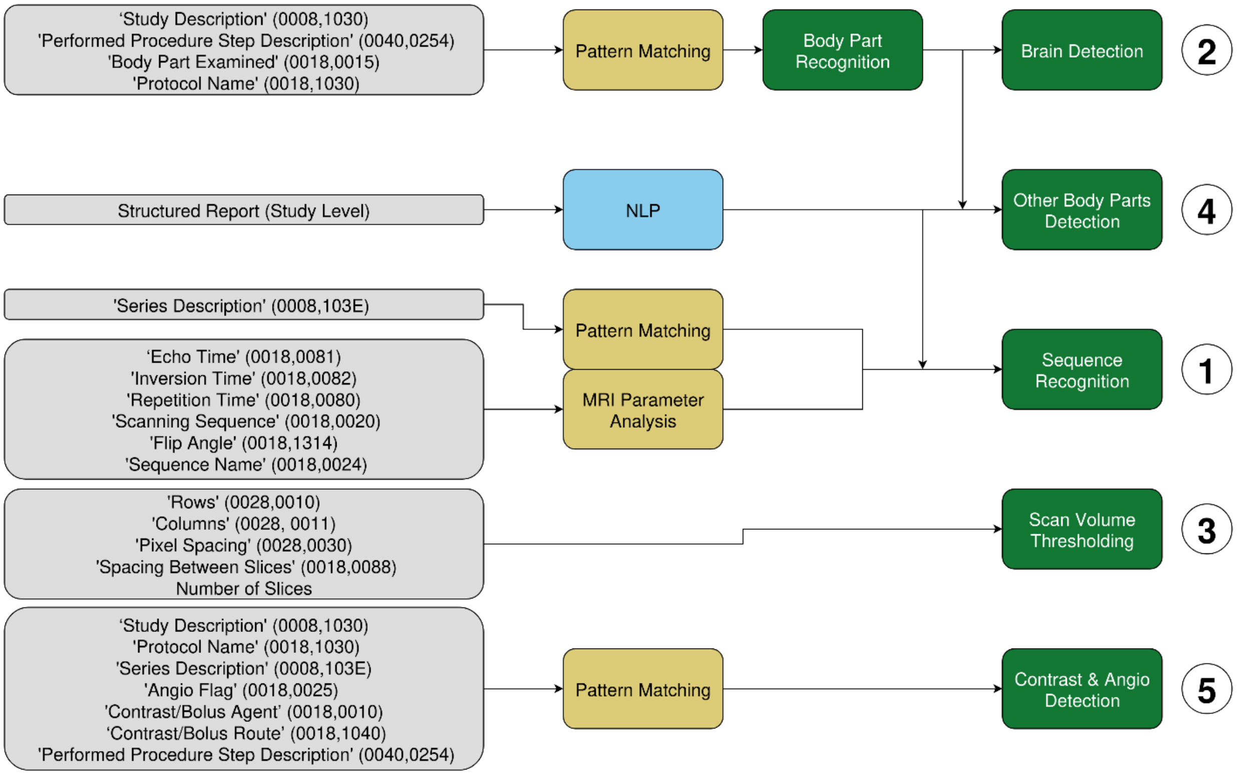

Figure 4. The exclusion process for the SCANDAN cohort. It is based on the automatic labelling (in yellow, including the pattern matching and the MRI parameter analysis) and the NLP of the radiological report (in blue). The steps were performed in the order from 1 to 5, numbered on the right. 1) Non T1/T2/FLAIR scans are excluded. 2) Scans without a brain are excluded. 3) Scans too small to contain a full brain are excluded. 4) Scans with other body parts (such as the spine) are excluded. 5) Contrast and angio scans are excluded.

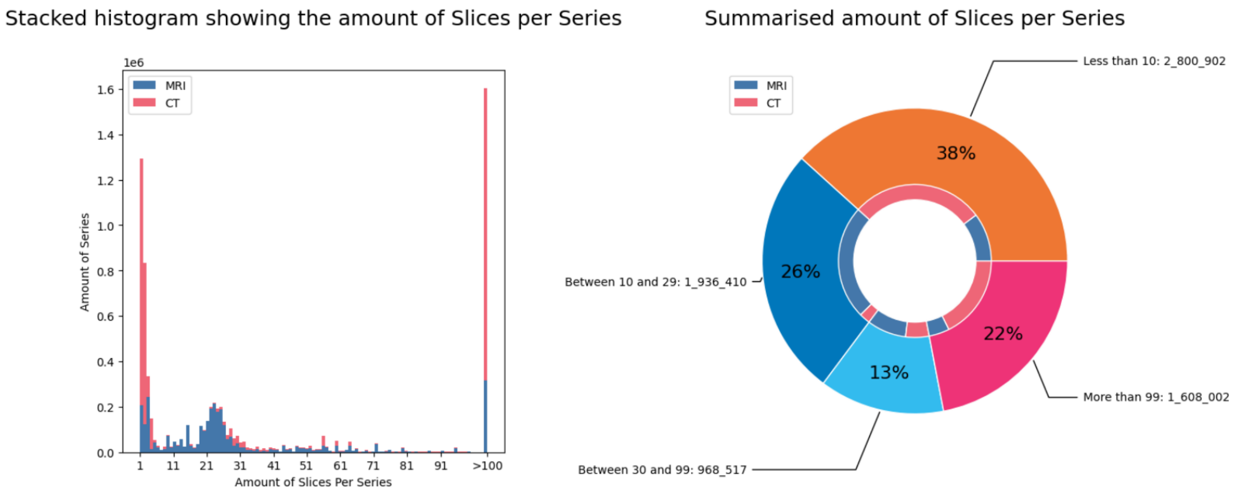

Figure 5: The distribution of slices per series within the data available through the BHD, for both MRI and CT. The stacked histogram shows the 2D MRI have a normal distribution of scans centered around 22, while the 3D MRI have over 100 slices. The CT have mostly more than slices. Both modalities have a large amount of localiser scans with under 10 slices.

Revised clean manuscript
Click here to access/download;Manuscript;Methodology paper v10_final.docx

A large dataset of brain imaging linked to health systems data: curation and access to a whole system national cohort from NHS Scotland

Michael P J Camilleri[1,2]*

Dorian Gouzou[3]*

Salim Al-Wasity[4]

Muthu R K Mookiah[4]

María Valdes Hernandez[3]

Bea Alex[5]

Sotirios A. Tsaftaris[2]

Andrew Brooks[6]

Ruairidh MacLeod[6]

Honghan Wu[7,8]

Brenda Bauer[3]

Claire Grover[5]

Parminder Reel[9]

Susan Krueger[9]

Richard Tobin[5]

J. Douglas Steele[4]

Grant Mair[3]

Joanna Wardlaw[3,10]

Alexander Doney[11]

Emanuele Trucco[1]**

William Whiteley[3,7,12]**

[1] Computing, School of Science and Engineering, University of Dundee, Dundee, UK.

[2] School of Engineering, University of Edinburgh, Edinburgh, UK.

[3] Institute for Neuroscience and Cardiovascular Research, School of Medicine, University of Edinburgh, UK.

[4] School of Medicine, Ninewells NHS and University Hospital, Dundee, UK.

[5] School of Informatics, University of Edinburgh, Edinburgh, UK.

[6] Edinburgh Parallel Computing Centre, University of Edinburgh, Edinburgh, UK.

[7] Usher Institute, School of Medicine, University of Edinburgh, Edinburgh, UK.

[8] School of Health and Wellbeing, University of Glasgow, Glasgow, UK.

[9] Health Informatics Centre, School of Medicine, University of Dundee, Dundee, UK.

[10] UK Dementia Research Institute Centre at the University of Edinburgh

[11] Cardiovascular Research, School of Medicine, University of Dundee, Dundee, UK.

[12] Health Data Research UK, London, UK

* equal contribution

** equal contribution

Correspondence: william.whiteley@ed.ac.uk

ABSTRACT

We present the design and implementation of a data curation framework to generate a large-scale clinical brain imaging dataset suitable for artificial intelligence (AI) enabled image analysis. The dataset is accessible through the Brain Health Data (BHD) initiative, which includes approximately 417,000 magnetic resonance imaging (MRI) and 846,000 computerized tomography (CT) head scans, linked electronic health records (EHRs), and associated free-text imaging reports from clinical practice between 2010 and 2018 in Scotland, exceeding 185 TB in size. The data curation framework was developed during the SCottish AI in Neuroimaging to predict Dementia and Neurodegenerative Disease (SCANDAN) study, which used a subset of 45,000 MRI from the BHD for dementia prediction.

We describe the processing of the BHD metadata and our multilabel classification output. We discuss the strengths of the BHD, including clinical relevance thanks to its unprecedented scale, population-wide representativeness of a national free-at-the-point-of-delivery healthcare, long-term follow-up to neurodegenerative disease, and real-world variability. We describe the challenges and lessons learnt in developing a framework to curate data, including the time needed to obtain permissions, the need for easily accessible, secure, responsive and affordable computational environments, the variability of clinical data, and the challenge of extracting linked clinical data and images at scale.

This resource will be crucial for clinical research, fostering the development of personalized medicine approaches, and fast-tracking the implementation of AI models in clinical workflows. We encourage the use of the BHD data through a streamlined application to the Public Benefit and Privacy Panel for Health and Care via the Data Research and Innovation Service of Public Health Scotland (eDRIS).

INTRODUCTION

Brain imaging plays a crucial role in the diagnosis of neurological disorders. However, clinical imaging services are under great demand, highlighting the need for new tools to improve radiology workflows. These tools should accelerate image assessment, reduce the workload for radiologists, and ultimately improve patient care. Artificial intelligence (AI) methods show promise for faster diagnosis for example in acute ischaemic stroke[1] and similar improvements are possible in head injury, neurodegeneration, dementia and brain cancers [2,3]. To develop and test AI models that are clinically relevant, researchers need access to large datasets of clinically acquired images, with sufficient computing resources, and secure, ethical data provision.

A survey conducted between December 2024 and February 2025 across UK secure data environments revealed the lack of brain imaging resources with nationwide coverage. For instance, the Diagnostic Imaging Dataset curated by NHS England includes patient-level metadata on the 501 million diagnostic imaging procedures performed in NHS England since April 2012, but it lacks imaging data and associated reports. [4]

The preparation of large repositories of routinely collected imaging data is challenging, particularly in privacy-protecting secure data environments. Despite adherence to Digital Imaging and Communications in Medicine (DICOM)[5] standards, real-world medical imaging datasets vary significantly in quality, format, and acquisition protocols, which makes standardisation across different imaging sources necessary. Determining imaging sequences (e.g., T1- or T2-weighted magnetic resonance imaging (MRI)) is essential for analysis but can be difficult in practice. DICOM meta-data tags provide rapid but sometimes unreliable classification, while image-based classification is more accurate but computationally demanding, and not free from uncertainty. [6,7] Natural language processing (NLP) of radiology reports can facilitate sequence identification and filter out scans with artefacts or missing structures. However, automated image quality assessment is paramount. Pre-processing and data retrieval is easier with automation of pipelines and integration of structured clinical records.

Compliance with governance frameworks is important to access large-scale unconsented clinical imaging datasets within safe havens, and needs ethical approval, data governance approval, and compliance with privacy regulations, all with costs. These administrative barriers, although necessary, can significantly delay or completely deter research.

To address these challenges, we developed the Brain Health Data (BHD) framework, which unifies all clinical brain imaging data acquired in Scotland with linked clinical information, to facilitate access to data through the Electronic Data Research and Innovation Service (eDRIS) of Public Health Scotland (PHS).[8] The data within the BHD framework includes approximately 417,000 MRI and 846,000 computerized tomography (CT) head scans, linked electronic health records (EHRs), and free text radiology reports collected between 2010 and 2018. It offers clinical relevance with its unprecedented scale, population-wide representativeness of healthcare, long-term follow-up to neurodegenerative disease, and real-world variability. To curate this large-scale clinical brain imaging dataset in a format suitable for AI analysis we

designed and implemented a data processing pipeline within the SCottish AI in Neuroimaging to predict Dementia and Neurodegenerative Disease (SCANDAN) study, which aimed to develop AI algorithms for reliable dementia risk estimation from routine brain imaging and clinical records. This paper describes SCANDAN's methods and outputs, highlights key lessons learned from working within Scotland's data governance frameworks, and describes how to access the data through the BHD.

METHODS

Permissions and research governance

The SCANDAN study obtained multicentre research ethics permission from the North of Scotland Research Ethics Committee (23/NS/0017). Permission to access the data for SCANDAN and the pilot phase for the BHD was provided by the NHS Scotland Public Benefit and Privacy Panel for Health and Social Care (PBPP), which scrutinises applications for access to NHS Scotland health data for non-direct care (PBPP applications 2223-0200 and 2223-0005 respectively). [9] During the application process and throughout the study, the SCANDAN team engaged with several Scottish public and patient groups.

Computing environment

The Scottish National Safe Heaven (NSH), commissioned by PHS, where all processing is done, is hosted in Edinburgh Parallel Computing Centre's (EPCC) Trusted Research Environment (TRE), a secure infrastructure which currently hosts twelve Safe Havens. Each Safe Haven is operated under the "Five Safes" framework [10] and the Scottish Government Charter for Safe Havens.[11] Researchers access a secure data sharing and analysis environment with a virtual desktop, under the terms and conditions prescribed by the data controllers. Standard software packages such as R and Python are available in the NSH; additional software packages can be installed from repositories such as the comprehensive R archive network (CRAN) and the Python package index (PyPI). Safe Havens have access to large shared-memory, high-performance computer clusters, including one with graphical processing unit (GPU) accelerators for large-scale analysis. For example, SCANDAN was provided a virtual environment with a GPU (NVIDIA A100 40GB), large storage (several TB) and RAM (100GB). All EPCC Safe Haven Services are operated at EPCC's Advanced Computing Facility, located in Edinburgh, Scotland. The EPCC TRE is accredited by ISO27001[12] for information security practices and self-certified under Cyber Essentials and NHS Digital's Data Security and Protection Toolkit (DSPT). In addition, the NSH is accredited under the Digital Economy Act 2017 by the UK Statistics Authority, and all Safe Havens in the TRE are operated to the same standard.

Data sources

PHS' eDRIS provided brain CT and MRI head studies in adults performed in Scotland between 2010 and 2018 from the Scottish Medical Imaging (SMI) service [13]. Study refers here to a complete imaging session, encompassing all images obtained during a single scanning session. Each scan contains three hierarchical levels: study, series, and images. Within each study,

there are one or more series that group together images acquired using the same imaging technique and settings. Each series is, in turn, composed of multiple single two-dimensional images or "slices”. A patient may have had multiple independent studies. Additionally, imaging reports are associated with studies and contain textual information regarding the imaging process and clinical interpretation of images. eDRIS and EPCC linked studies to patients deterministically with pseudonymized identifiers based on the Community Health Index (CHI) number, which is the unique patient identifier used across NHS Scotland. We linked them with outpatient records (SMR00), hospital admission records (SMR01), dementia records from mental health hospitalisations (SMR04), cancer registry (SMR06), community dispensed prescriptions from Prescribing Information System (PIS), death records (National Records of Scotland (NRS) and demographics (birth year, sex, deprivation index) since the year 2000. All the data was processed and stored within the NSH.

Data availability

The data on 830,000 patients was provided to SCANDAN and is available through the BHD. It includes 417,000 MRI studies, 846,000 CT studies and 1.8 million radiological reports. The studies contain 3.37 million MRI series and 3.15 million CT series. Figure 5 shows the distribution of slices per series for both CT and MRI for which the metadata was available at the start of the project. There were 356 million events from EHR, divided between the outpatient emergency and inpatient records (38 million), death records (327,000), prescription records (312 million) and accident and emergency records (4.5 million). For the 409 million DICOM slices available (MRI and CT), DICOM metadata were limited by the governance approval, with each tag being subject to approval. Consequently, the accepted metadata were provided separately in CSV format.

SCANDAN Project

The SCANDAN sequential work packages (WP) are illustrated in Figure 1. The NLP WP identified MRI sequences, CT type and brain pathologies from the radiological reports. The dementia labelling WP phenotyped dementia with EHRs. The cohort building WP selected a subset of MRI studies to carry out dementia classification. The image cataloguing WP labelled DICOM series with body part imaged, sequence, and presence of contrast and then filtered out non-desired scans based on the label. Images were then processed for AI analysis.

SCANDAN: Natural language processing of brain imaging reports

We applied a clinical NLP tool, the Edinburgh Information Extraction for Radiology (EdIE-R), [14,15] which was originally developed and validated for radiology reports of brain imaging in the Edinburgh Stroke Study and NHS Tayside [16]. EdIE-R processes radiology reports through a pipeline that identifies entities, detects negation, extracts relationships and assigns document-level labels to identify phenotypes. The tool was later adapted and validated for use with data from other areas in Scotland provided by Generation Scotland [13]. EdIE-R can extract 24 distinct phenotypes, including different stroke types (ischaemic, haemorrhagic and underspecified, with time and location details), brain tumours (meningiomas, gliomas, metastases or underspecified), small vessel disease, microbleeds, atrophy and other abnormalities. Additionally, it marked up MRI sequence types (T1, T2, and FLAIR).

To improve data selection, EdIE-R was enhanced to identify scans of non-head and non-brain body parts, and flag them for exclusion. We improved the tool's ability to identify where distinct sections begin and end within reports, such as the boundary between the clinical history preamble and the main report body, enabling us to extract phenotype mentions exclusively from the relevant report text.

EdIE-R contains several processing components. After pre-processing and linguistic analysis (e.g. tokenisation, sentence detection, lemmatisation and part-of-speech tagging) of the text in the input radiology report, EdIE-R performs named entity recognition, negation detection and relation extraction before conducting document-level classification of the 24 phenotypes. The output is the radiology report and its accompanying metadata as well as the information identified by EdIE-R represented in XML format which was then converted to CSV for follow-on analysis. It is keyed by the study identifier and does not contain information about a specific series.

The refined EdIE-R pipeline [15] was applied to all radiology reports in the SCANDAN project, producing structured outputs to guide data selection for image analysis. By processing radiology reports within the Scottish NSH, the tool allows exclusion of scans (e.g., those showing tumours or non-brain regions) and served to validate outputs from imaging type classification and phenotype extraction.

Structured report DICOM contains TextValue elements of various kinds. Some are clinical reports, others contain non-clinical information. We aimed to select the best clinical report in each study for NLP processing. Not all study directories contained structured reports, and some contained more than one. For the latter, the process of choosing a report was as follows. From inspection of examples, it appeared that DICOM files containing real reports normally contained exactly one TextValue element. If a study contained one or more such DICOM files, we used the largest of those. In the cases where no report contained exactly one TextValue element, we just used the largest DICOM file, and processed the first TextValue element. If this was not a clinical report it would usually be marked as “empty” or “nocontent” by the NLP pipeline. Some reports were withheld because they were potentially identifiable, which accounts for studies with no report or no real report.

SCANDAN: Phenotyping dementia

We follow the phenotype specification for dementia [18] based on prior studies in Scottish EHRs [19]. Dementia was defined as the presence of relevant ICD-10 codes, and, for the specific case of Alzheimer's disease (AD), the prescription of AD medications. Each patient interaction with the health system, taken from the EHR, was used including a single stay in hospital, multiple consecutive stays, a prescription, or a death record. We defined labels for ‘any dementia’ and five dementia subtypes: AD, vascular dementia (VaD), other rare dementias, unspecified dementia and possible dementia. The subtype was defined as the most frequently occurring dementia phenotype in each person's electronic record. (Table 1). All individuals with a dementia label were categorised as cases, and individuals with no mention of dementia in any record were considered controls.

SCANDAN: Cohort building

We built a matched case-control study cohort with MRI brain images. A matched case-control design was chosen for several reasons. First, most deep learning and other algorithms work best with balanced cases and controls. Second, we had limited computing capacity at the beginning of the project. Third, the rate of image delivery was limited by the need to copy data from a preparation area to a research area which had limited storage capacity. We selected images with no NLP label of tumour or haemorrhagic stroke in the radiology report from patients who were aged over 40 years at the time of scan and had an associated EHR record. We excluded dementia cases without scans taken more than one year before the time of diagnosis. For each individual, the first study chronologically was chosen. Dementia cases were matched to controls based on age at the time of scan (within one year of the matched case) and recorded sex from the linked demographic information. Cases with no matched controls were discarded. Age and sex matching was verified by analysing the resulting distributions over the entire cohort. A table was generated containing the identifier of the selected patient, their match, the selected study, demographic information, and the dementia ground truth.

SCANDAN: Data validation and quality control

To generate ground truth labels for an initial evaluation of the automatic labelling process described in the next section, we developed a custom python-based graphical user interface (GUI) optimized for MRI and CT DICOM files. The GUI allowed users to load DICOM images from a single or nested folder structure. It utilized DICOM header metadata to: stack slices according to the acquisition order using the DICOM tag 'Instance Number' (0020,0013), in ascending, descending, or interleave format, constructing and saving the 3D volumes for subsequent analysis; filter CT scans using the tag 'Modality' (0008,0060) and adjust their intensities, e.g. brain-windowing, using the tag 'Rescale Intercept' (0028,1052); determine the orientation of the imaging planes (i.e., axial, sagittal, or coronal) using the tag 'Image Orientation (Patient)' (0020,0037) to display mid-axial, mid-coronal, and mid-sagittal views for assessment; and calculate the aspect ratio using the 'Slice Thickness' (0018,0050) and 'Pixel Spacing' (0028,0030) tags for accurate scaling and visualising of mid-view slices within the designated display area.

Randomly sampled example scans (1,000) were selected prior to the large data delivery without stratification for image review. Among those, 287 were excluded for potential disclosive information. The remaining 390 MRI and 323 CT were annotated by five experts (3 clinicians and 2 trained imaging scientists) with modality, sequence, presence of contrast, lesion and artefacts, presence of full brain, and presence of body parts (Figure 2). The 713 scans were evenly divided among the five annotators, with a subset of 100 images overlapping for cross-validation. The Generative model of Labels, Abilities, and Difficulties (GLAD) [14] probabilistic framework was used to estimate the true label for each image while accounting for annotator expertise and image difficulty, by using the overlapping 100 annotations as "truth" and correcting the rest of the annotations.

The ground-truth labels were compared with the labels obtained from the automatic labelling. Scans sequence type and contrast presence were further reviewed by a neuroradiologist and an

experienced imaging scientist independently for the disagreement with the automatic labels, or for previous “unknown” or “uncertain” annotations. A third round of annotations resolved disagreements between the neuroradiologist and the imaging scientist. The presence of brain, of other body part and the fullness of the brain was re-annotated by a trained image scientist as an independent re-annotation. After re-annotation, the ground-truth was updated using the most recently agreed version. We report the final comparison of the automatic tools and the manual annotations.

SCANDAN: Identification of MRI scan type and sequence

DICOM tags were used to produce five labels for each image series: imaging sequence, presence of brain, presence of other body part, angiography, and imaging with contrast. We aimed to retain MRI series with sequences T1, T2 and Fluid-Attenuated Inversion Recovery (FLAIR), that contained a brain and no other body part than the neck, without angiography or contrast, and with a 3D image volume of over 5 litres. The volume was empirically determined on another study using Scottish medical data, compared to the manual annotations, and visually asserted to separate two different normal distribution of scans volume.[20] These labels were subsequently combined with the results from the NLP tool, the MRI acquisition parameters, and the computed volume of the image series, to exclude those which did not meet SCANDAN criteria. Figure 4 illustrates the methods and data sources for the labelling and exclusion of images.

To produce these labels, the DICOM tags were parsed with regular expressions.[21] For example, the expressions /(?i)(?<!pa)t2/ (case insensitive and ignoring occurrences starting with “pa”) and /*se2d1/ were associated with the intermediate label "tmp-T2" (T2-weighted), while the expressions /TOF/ and /MRA/ were associated with the intermediate label "tmp-MRA" (MR angiography). Then, the final labels were created by grouping all the intermediate labels of a series. For example, for an image series to be labelled "T1", it had to match the intermediate label "tmp-T1", and could optionally match the intermediate labels "FLAIR", "GRE" (gradient echo), and “FAT SAT” (fat saturation), which are not T1-weighted exclusive, but no any other intermediate label.

For sequence identification, the DICOM tag 'Series Description' (0008,103E) was used. To identify the body part, we used the tags 'Body Part Examined' (0018,0015), 'Protocol Name' (0018,1030), 'Performed Procedure Step Description' (0040,0254) and ‘Study Description' (0008,1030). Angiograms were identified with the tags ‘Angio Flag’ (0018, 00), ‘Study Description' (0008,1030), 'Protocol Name' (0018,1030) and ‘Series Description' (0008,103E). Contrast identification used ‘Study Description' (0008,1030), ‘Contrast/Bolus Agent’ (0018,0010), ‘Contrast/Bolus Route’ (0018,1040), 'Performed Procedure Step Description' (0040,0254) and 'Series Description' (0008,103E).

The results from the NLP tool provided additional information for the sequence identification and the presence of other body parts, validating the presence of a sequence within a study. Finally, to complement the identification based on the series description the MRI sequence was also defined with the tags ‘Echo Time' (0018,0081), 'Inversion Time' (0018,0082), 'Repetition Time' (0018,0080), 'Scanning Sequence' (0018,0020), 'Flip Angle' (0018,1314), 'Sequence Name'

(0018,0024) based on "optimal" value [7-8] adapted to the data through observation on manually annotated data.

Each regular expression rule was based on prior research carried on in-house clinical studies. They were expanded to ignore conflict and formatting due to the greater distribution of value from the 35 hospitals which the data originates from. The most common occurrence for each DICOM tag were compared to their label using the metadata of the entire cohort, to ensure the rules were not including unwanted samples. Finally, they were refined to agree with the manual annotation during each re-annotation.

RESULTS

SCANDAN: Output Dataset

Taking the first chronological study in each sequence of studies (MR or CT) for each person gave 1.1 million studies of which 311,000 were MRI and 789,000 CT. Among these, 16,000 MRI and 119,000 CT were associated with a record of dementia. After applying exclusion criteria, as described in Table 3, 10,709 MRI and 57,242 CT dementia cases were age and sex matched with the same number of healthy controls. We eliminated 1,171 MRI and 3,302 CT dementia cases with a text report containing a mention of tumour or haemorrhagic stroke; 70 MRI and 100 CT dementia cases due to the patient being under 40 at the time of scans; and 4,869 MRI and 58,779 CT cases because the scans occurred within one year prior to the dementia diagnosis.

Of the 1,481,643 study directories, 449,369 had no structured report, 655,450 study directories contained exactly one report and 376,824 study directories contained more than one report. Each selected report was processed using the EdIE-R NLP pipeline. Across the full dataset, the most frequently detected phenotypes were small vessel disease (25.0% of studies) and atrophy (23.2%), reflecting their high prevalence in an ageing clinical population. Ischaemic stroke findings were also common (e.g. old deep ischaemic stroke was detected in 9.4% of studies), while haemorrhagic stroke subtypes were comparatively rare (0.2–2.9%). Tumour-related findings were detected in 0.4–4.1% of studies, depending on subtype. Regarding imaging sequences, T2 was the most frequently recorded (13.4% of studies), followed by T1 (8.3%) and FLAIR (7.8%). These NLP-derived labels formed the basis for the phenotypic exclusion criteria applied to the dementia cohort.

Of the 21,418 MRI scans requested, 21,197 were successfully received, with 221 being excluded for privacy reasons. These studies contained 128,257 series of which 73,457 were identified as T1, T2 or FLAIR, 18,681 series were localisers, 4,372 as unknown, 30,267 were other MRI sequences (DWI, SWI, T2* etc.), and 1,464 series had a series description that did not differentiate T1 and T2*. Table 5 describes the filtering process making use of the DICOM labelling process, which resulted in the exclusion of 2,641 FLAIR, 11,820 T1 and 10,863 T2. After restricting the selection to the first chronological series for each study, 41,966 series were kept from 15,558 studies. Later, 277 studies were excluded when they failed to convert to NIfTI and subsequent process.

The MRI studies requested originated from 35 hospitals across Scotland using 27 unique MRI scanners models were identified (14 models with <100 studies), with 60% of the studies using a

Siemens model, 20% General Electric (GE) and 20% Philips, and a handful from 2 other manufacturers. 94% of the scans were done with a 1.5 Tesla, and 6% a 3 Tesla MRI scanner.

The MRI cohort contained 8,145 cases (53.2% female) and 7,236 controls (54.1% female) (Table 4). The mean age at scan was 74 years. The mean time from scan to first mention of dementia was 5 years for cases, and the mean follow-up time for controls was 6 years 9 months. Of the 8,145 dementia cases there was non-exclusive record of AD in 3,774, vascular dementia in 3,386, unspecified dementia in 3,784 and other dementia types in 508. The mean number of hospitalisations in the year prior to scan was 1.1 (standard deviation ([SD] 1.52) for cases and 1.0 (SD 1.50) for controls. During the same period, the mean number of prescriptions was 15.4 for cases and 14.2 for controls.

SCANDAN: Data validation and quality control

For simplicity, we refer to the results of the manual annotations as "annotations", and the results of the automatic tools described in Figure 4 which are compared to the manual annotations as "labels".

During the first round, 707 annotations were obtained from 713 images. Four images could not be read due to acquisition errors. Two images were only partially annotated due to visual perception errors and discarded. In 29 (4.5%) cases, image modality was wrongly annotated because the scans contained only one slice (i.e. a localiser) or did not contain a brain. Of CTs, 24 (7.52%) were classed as 'Unknown'. In the labelling process we used the previously validated DICOM tag 'Modality' (0008,0060) to identify CT and MRI scans. The identification of the body parts was easier for the annotators than the modality or sequence type. “Unknown” was given for 23 (3.3%) series when annotators were questioned whether they contained a brain or not, 40 (5.7%) when questioned if the brain was acquired in full, and in 36 (5.1%) series the annotators could not assert whether there was another body part. Annotators could not identify the sequence type for 85 (12.0%) series and the presence of contrast in 104 (14.7%) series.

In this first round of annotations, the main disagreements between annotations and labels were the presence of non-brain images or localisers. For further analyses, the scans labelled as 'localiser' in the first round of annotations, defined as series with less than 15 slices, were ignored. If the annotation and label agreed on the absence of brain, the images were not re-annotated.

In the second round of annotations, images with an unknown sequence type (16 scans), and those with disagreement between label and annotation ('T1', 'T2' or 'FLAIR', 48 scans), were re-annotated. Additionally, a subset of images was selected from 66 with partial agreement between at least one sequence label and the annotation, to validate commonly occurring combination of labels which were not similar (e.g. 'T1' + 'GRE' instead of 'T1' + ’T1’). Scans which contained at least one mention of 'T1', 'T2' or 'FLAIR' in either the annotation or the label were re-annotated for presence of contrast when disagreement was found or when they were annotated as 'Unknown'. Series with 'Unknown' annotation for questions regarding brain presence (13 scans), other body part presence (8 scans) and whole brain (11 scans) were also re-annotated. Disagreement between the annotation and the labels were also re-annotated,

respectively 7, 143 and 20 scans. In case of a whole brain, the disagreement was ignored if other body parts were present in both the label and the annotation. In total, 143 series were re-annotated for the presence of brain and other body parts, and for full brain coverage. Additionally, there were 84 images re-annotated for sequence type and contrast.

To resolve conflict between the two re-annotators, or between them and the labelling tools, 27 series were then annotated a third time. Some conflicts could not be resolved, such as 7 images having the same 'Series Description' (0008,103E) tag value, and thus the same label. Three of them were identified as T1 and four as T2* by the two annotators in agreement.

Between each round of annotation, the regular expressions used by the labelling tools were updated to reflect previously unknown, and to solve conflicting information and errors.

The results of the labelling tools compared to the final annotation as ground truth were very good. The true positive rate ranged from 87% to 97% and the positive predictive value from 81% to 99% (Table 2). For consistency with our metrics, we evaluated the absence of contrast and other body part respectively as “positive”. This value excludes localisers for sequence type, and series without presence of brain for 'whole brains'. The lower precision for detection of other body parts is explained by the lack of mention of any parts in the different DICOM tags, sometimes due to missing data, as well as the detection of some other head parts, such as the jaw, without mention of the brain, which often, but not always, indicate non brain scans. The lower recall for the absence of contrast is caused by the low number of studies that used intravenous (IV) contrast. During a scanning session that used IV contrast, a first image will normally be captured free of contrast, prior to the injection, however, the ‘Study Description' (0008,1030) will indicate the presence of IV contrast nonetheless for this first series, as was commonly found.

SCANDAN contribution and BHD data

The SCANDAN project produced data which were added to the BHD. In addition to the 1.2 million brain studies from 830, 000 patients, the 1.8 million radiological report and the 356 million EHR available as raw data, researchers can also access five additional tables: 1. summary of the valid radiological reports generated by the NLP; 2, dementia phenotyping table, with dementia subtype probability and date of diagnosis; 3. patient history, curated and listing all relevant information from all EHR; 4. the manual annotations of the 708 scans; 5. the labelling of the 21K MRI of the SCANDAN cohort for MRI sequence, body part imaged, brain fullness and contrast presence. The latter is planned to be expanded to the totality of MRI and then CT scans.

Permissions and governance

The SCANDAN application to the PBPP, which included an industry partner and aimed to develop an AI algorithm, required 210 days spanning 4 iterations for approval from the initial submission and over 17,000 words across 33 pages.

However, with the development of the BHD, researcher can now apply to PBPP to access these data with a shorter application and streamlined process. The data flow and linkage process for the BHD framework are schematically illustrated in Figure 3. Researchers can log into a

workspace running in the NSH with data and tools to perform analysis. To run externally developed tools, they can build a container outside the NSH and pull it from a public registry after approval. It should be noted that no data leaves the NSH during this process. The TRE is divided in several zones (Figure 3). The blue zones, where eDRIS store the data, are not accessible to the researchers. Researchers have access to green zones with access to subsets of the data, as defined by their access permission.

PHS eDRIS will prepare suitable subsets of the data for a particular research group and copy it to their working space. Any results required outside the NSH, e.g., for publication, are subject to disclosure control performed by eDRIS.

DISCUSSION

To our best knowledge, BHD is the first large-scale, curated brain imaging clinical dataset relevant to dementia research that is available to researchers via moderated public access. The dataset offers several advantages in addition to its large size: clinical relevance, long-term follow-up, co-location with a GPU cluster in a safe haven, greater population representativeness compared to many research cohorts, and accessibility for clinical researchers. The resource continues to grow in data size and computing power.

Working with health systems data presents challenges. One of them is the time taken for governance approval. In our case, if governance had been applied for after the funding had been awarded, it would have represented 58% of a 1-year postdoctoral award. This would not only impact negatively on career development of the post holder but also delay the project goals, an issue addressed by the streamlined process of the BHD Data provision was initially constrained by the limitations of the virtual machine environment, limited staff availability, and increased procedural complexity resulting in delayed access to imaging data and complicating project planning. The experience gained through the SCANDAN project, which piloted the access to the data, allowed PHS to streamline the governance process and improve the data provisioning to future projects. It is important however, to note that all research outputs generated within the NSH must undergo review by PHS staff prior to release.

Most imaging research is based on uniformly acquired research data. In contrast, clinical scans acquired in a routine free-at-the-point-of-service healthcare are sometimes incomplete, may be obscured by movement or other artefacts, show signs of non-relevant pathologies, may have been obtained with non-standardised protocols, and on different machines. However, such real-world data with inherent variability is essential for the development of software tools suitable for robust applications in clinical practice where such heterogeneity is the norm.

Using electronic health records for dementia diagnosis has limitations. Currently, primary care data are unavailable through PHS and thus cannot be provided by the BHD. Hence, we relied on recorded diagnosis after an inpatient stay or death. Hospital and death records under-ascertain (false negatives) dementia in the short term and have modest reliability for dementia subtypes [22]. However, they have also previously shown high positive predictive value for all dementia diagnosis [14]. Referral reasons for scans acquisitions are not currently available, although further NLP work with reports could achieve this.

The use of head scans does raise privacy concerns due to facial recognition risks. We have mitigated these by working only in a safe haven environment, examining only brain slices, prohibiting facial reconstruction, limiting access to approved researchers, who accept the restrictions and conditions of working in the NHS specified in the eDRIS User Agreement which includes PHS strictly checking all outputs from the secure environment, to exclude any identifiable data. Future work aims to further mitigate privacy risks by limiting the need for direct human access to data, for example by implementing software via containers. However, this work needs training of the research community, better labelling of metadata (so the data is truly FAIR), and further development of technology within the NSH environment.

There are many opportunities for further linkage to other datasets (for example community retinal imaging [23]). Such work will require further engagement with public contributors, use of federated analysis and federated learning with ongoing adoption of tools and techniques to assess disclosure risks of different AI models.

The SCANDAN project piloted the access to the data now provided through the BHD. While its primary goal was to establish a proof of concept for dementia classification using clinical data, it produced several secondary outputs which are now available to other researchers using the BHD data. As more projects will use the data, additional output will be added, compounding with time to an unvaluable resource for brain imaging research. Researchers can access the BHD data by applying to PBPP via eDRIS. Proposals must demonstrate a clear public benefit, and researcher- generated outputs must be added back to the dataset so every project strengthens the next. We strongly encourage cross- group collaboration. The resources available through the BHD are growing in terms of data availability, storage capacity, and computing power that are provided to researchers. We hope that this, and similar global initiatives, will ultimately contribute to improve the brain health of people worldwide.

FUNDING

This work was supported by NEURii, a collaborative partnership involving the University of Edinburgh, Gates Ventures, Eisai, LifeArc and Health Data Research UK (HDR UK). We acknowledge the eDRIS team (Public Health Scotland) for their support in obtaining approvals, the provisioning and linking of data and facilitating access to the National Safe Haven. The Brain Health Data Pilot is supported by Alzheimer’s Disease Data Initiative (ADDI) and HDR UK with funding to the University of Edinburgh.

CONFLICTS OF INTEREST

MVH and JMW are supported by Row Fogo Charitable Trust (Grant no. BRO-D.FID3668413). JMW was supported by the UK Dementia Research Institute (award no. UKDRI –4002 and 4205, DRIEdi17/18, and MRC MC_PC_17113) which receives its funding from DRI Ltd, funded by the UK Medical Research Council, Alzheimer’s Society and Alzheimer’s Research UK. ST acknowledges support of the UKRI AI programme, and the Engineering and Physical Sciences Research Council (EPSRC), for CHAI - Causality in Healthcare AI Hub [grant number EP/Y028856/1]. WW and HW are supported by HDRUK.

REFERENCES

1. Westwood M, Ramaekers B, Grimm S, Armstrong N, Wijnen B, Ahmadu C, et al.. Software with artificial intelligence-derived algorithms for analysing CT brain scans in people with a suspected acute stroke: a systematic review and cost-effectiveness analysis. *Health Technol Assess (Rockv)*. NIHR Journals Library; 2024; doi: 10.3310/RDPA1487.

2. Ferber D, El Nahhas OSM, Wölflein G, Wiest IC, Clusmann J, Leßmann ME, et al.. Development and validation of an autonomous artificial intelligence agent for clinical decision-making in oncology. *Nat Cancer*. Nature Research; 2025; doi: 10.1038/S43018-025-00991-6;SUBJMETA.

3. D'Adderio L, Bates DW. Transforming diagnosis through artificial intelligence. *NPJ Digit Med*. Nature Research; 2025; doi: 10.1038/S41746-025-01460-1.

4. NHS England. Diagnostic Imaging Dataset Annual Statistical Release 2023/24. 2024 Nov.

5. Larobina M. Thirty Years of the DICOM Standard. *Tomography*. Multidisciplinary Digital Publishing Institute (MDPI); 2023; doi: 10.3390/TOMOGRAPHY9050145,.

6. Liang S, Beaton D, Arnott SR, Gee T, Zamyadi M, Bartha R, et al.. Magnetic Resonance Imaging Sequence Identification Using a Metadata Learning Approach. *Front Neuroinform*. Frontiers Media S.A.; 2021; doi: 10.3389/fninf.2021.622951.

7. de Mello JPV, Paixão TM, Berriel R, Reyes M, Badue C, de Souza AF, et al.. Deep learning-based type identification of volumetric MRI sequences. *Proceedings - International Conference on Pattern Recognition*. Institute of Electrical and Electronics Engineers Inc.; 2020; doi: 10.1109/ICPR48806.2021.9413120.

8. : What is eDRIS? - Overview - Electronic Data Research and Innovation Service (eDRIS) - Health intelligence and data management - Resources and tools - Public Health Scotland. https://publichealthscotland.scot/resources-and-tools/health-intelligence-and-data-management/electronic-data-research-and-innovation-service-edris/overview/what-is-edris/ Accessed 2026 Mar 27.

9. : Public Benefit and Privacy Panel for Health and Social Care. https://www.informationgovernance.scot.nhs.uk/pbpphsc/ Accessed 2026 Mar 27.

10. : The Five Safes Framework. https://www.gov.uk/data-ethics-guidance/the-five-safes-framework Accessed 2025 May 28.

11. : Charter for Safe Havens in Scotland: Handling Unconsented Data from National Health Service Patient Records to Support Research and Statistics. https://www.gov.scot/publications/charter-safe-havens-scotland-handling-unconsented-data-national-health-service-patient-records-support-research-statistics/ Accessed 2025 May 28.

12. : ISO/IEC 27001:2022 Information security management systems. https://www.iso.org/standard/27001 Accessed 2025 May 28.

13. Baxter R, Nind T, Sutherland J, McAllister G, Hardy D, Hume A, et al.. The Scottish Medical Imaging Archive: 57.3 Million Radiology Studies Linked to Their Medical Records. *Radiol Artif*

Intell. Radiological Society of North America Inc.; 2024; doi: 10.1148/RYAI.220266/ASSET/IMAGES/LARGE/RYAI.220266.FIG2.JPEG.

14. Alex B, Grover C, Tobin R, Sudlow C, Mair G, Whiteley W. Text mining brain imaging reports. *J Biomed Semantics*. England; 2019; doi: 10.1186/s13326-019-0211-7.

15. : Software – Language Technology Group. https://www.ltg.ed.ac.uk/software/ Accessed 2025 Aug 14.

16. Wheater E, Mair G, Sudlow C, Alex B, Grover C, Whiteley W. A validated natural language processing algorithm for brain imaging phenotypes from radiology reports in UK electronic health records. *BMC Med Inform Decis Mak*. 2019; doi: 10.1186/s12911-019-0908-7.

17. Casey A, Davidson E, Grover C, Tobin R, Grivas A, Zhang H, et al.. Understanding the performance and reliability of NLP tools: a comparison of four NLP tools predicting stroke phenotypes in radiology reports. *Front Digit Health*. Switzerland; 2023; doi: 10.3389/fdgth.2023.1184919.

18. : Phenotype Library | Phenotype: Dementia Identification with EMR. https://phenotypes.healthdatagateway.org/phenotypes/PH1717/version/3973/detail/ Accessed 2026 Mar 21.

19. Doney ASF, Bonney W, Jefferson E, Walesby KE, Bittern R, Trucco E, et al.. Investigating the Relationship Between Type 2 Diabetes and Dementia Using Electronic Medical Records in the GoDARTS Bioresource. *Diabetes Care*. American Diabetes Association; 2019; doi: 10.2337/dc19-0380.

20. Reel PS, Al-Wasity S, Edwards C, Reel S, Mansouri-Benssassi E, Suveges S, et al.. Machine learning-based prediction of future dementia using routine clinical MRI brain scans and healthcare data. *medRxiv*. Cold Spring Harbor Laboratory Press; 2025; doi: 10.1101/2025.11.12.25340070.

21. : GitHub - SCANDAN-Team/SCANDAN-DICOM-labelling: Rules for DICOM tag based labelling · GitHub. https://github.com/SCANDAN-Team/SCANDAN-DICOM-labelling Accessed 2026 Mar 30.

22. McGuinness LA, Warren-Gash C, Moorhouse LR, Thomas SL. The validity of dementia diagnoses in routinely collected electronic health records in the United Kingdom: A systematic review. *Pharmacoepidemiol Drug Saf*. John Wiley and Sons Ltd; 2019; doi: 10.1002/PDS.4669.

23. Tochel C, Bernabeu MO, McTrusty A, Tatham AJ, Pead E, Buckmaster F, et al.. SCONe: a community-acquired retinal image repository enabling ocular, cardiovascular and neurodegenerative disease prediction. *BMJ Health Care Inform*. BMJ Publishing Group; 2025; doi: 10.1136/BMJHCI-2024-101236.

TABLES

Table 1: International classification of diseases 10 (ICD-10) and British national formulary (BNF) codes to define dementia subtypes. The definition of the codes follows the phenotyping employed previously.[18] * indicates a wild-card, meaning that all child-codes in the hierarchy are included.

Subtype	ICD10	BNF
Alzheimer's disease	F00* G30*	0411000
Vascular dementia	F01*	
Other rare dementias	F02*, G31.0, A81.0	
Unspecified dementia	F03, F05.1	
Possible dementia	F05.0, G31	

Table 2: The metrics of the comparison between the automated labels and the manual annotation as ground truth for image characteristics.

Algorithm identified image characteristics	Recall (%)	Precision (%)
sequence type	94.1%	98.6%
study contains brain	97.1%	91.0%
absence of other body parts	93.1%	81.9%
whole brain in study	96.4%	95.6%
absence of contrast in study	87.3%	95.4%

Table 3: Selection process for the MRI and CT cohorts, showing both the overall and dementia counts, with addition of characteristics

**Criterion**	**MRI**		**CT**	
	Overall	Dementia	Overall	Dementia
**With scan**	294,422	16,819	669,539	119,423
**+ No reported tumour or haemorrhagic stroke**	280,549	15,648	640,400	116,121
**+ Hospitalised electronic health record**	279,004	15,648	638,680	116,121
**+ Diagnosis other than 'possible' dementia**	275,970	15,648	627,911	116,121
**+ Age at scan > 40**	207,876	15,578	523,855	116,021
**+ Dementia diagnosis or follow-up > 1 year**	190,582	10,709	391,356	57,242

MRI: magnetic resonance imaging of brain; CT: computerised tomography of brain

Table 4: Distribution of subjects with mention of each dementia type and, controls grouped by key characteristics

		Alzheimer's	Vascular	Other or rare	Unspecified	Controls
**Sex**	Female	2,057	1,681	207	2,047	3,917
	Male	1,717	1,705	301	1,737	3,319
**Age in years**	Mean (SD)	73 (8.8)	75 (8.7)	67 (9.3)	74 (8.9)	74 (9.0)
	40-50	36	32	19	38	87
	51-60	282	169	90	227	434
	61-70	968	706	179	813	1611
	71-80	1,694	1,503	182	1,664	3,182
	81+	794	976	38	1,042	1,922
**SIMD**	Mean (SD)	2.8 (1.3)	2.7 (1.3)	2.8 (1.3)	2.7 (1.3)	2.8 (1.3)
	1	719	768	102	842	1429
	2	732	650	102	724	1380
	3	753	635	94	703	1381
	4	909	691	113	780	1523
	5	363	242	42	325	804
**Hospitalisation 1 yr before scan, Mean (SD)**		0.9 (1.4)	1.2 (1.5)	1.0 (1.6)	1.1 (1.5)	1.0 (1.5)
**Prescriptions 1 yr before scan, Mean (SD)**		14.5 (9.5)	16.1 (0.2)	14.3 (9.7)	15.6 (10.2)	14.2 (9.4)

SD: standard deviation; SIMD: Scottish index of multiple deprivation

Table 5: Selection process based on the automatic labelling for the MRI cohort. The “Original” column shows the amount of series for each sequence type. The "Selected" columns show the number of series kept after the selection process ready for analysis. Every column in between is associated with an excluding step and shows the number of series it excluded.

Sequence	Original	Brain	Whole Brain	Other Body Parts	Contrast & Angio	One Series per Study	Selected
FLAIR	16,871	-60	-211	-1,742	-628	-714	13,516
T1	27,627	-1,290	-4,670	-2,707	-3,153	-2,156	13,651
T2	28,959	-1,407	-4,828	-3,738	-890	-3,297	14,799

FIGURES

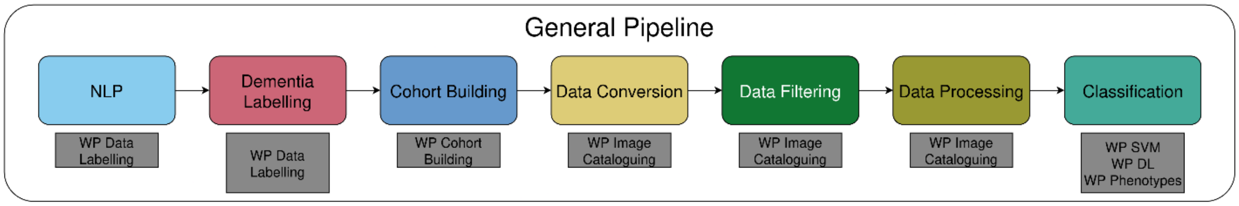

Figure 1: Work packages (WP) in the SCANDAN project: Data labelling, cohort building, image cataloguing, and processing for classification into being indicative of having dementia or not using deep learning (DL), support vector machine (SVM) and from the analysis of extracted imaging phenotypes.

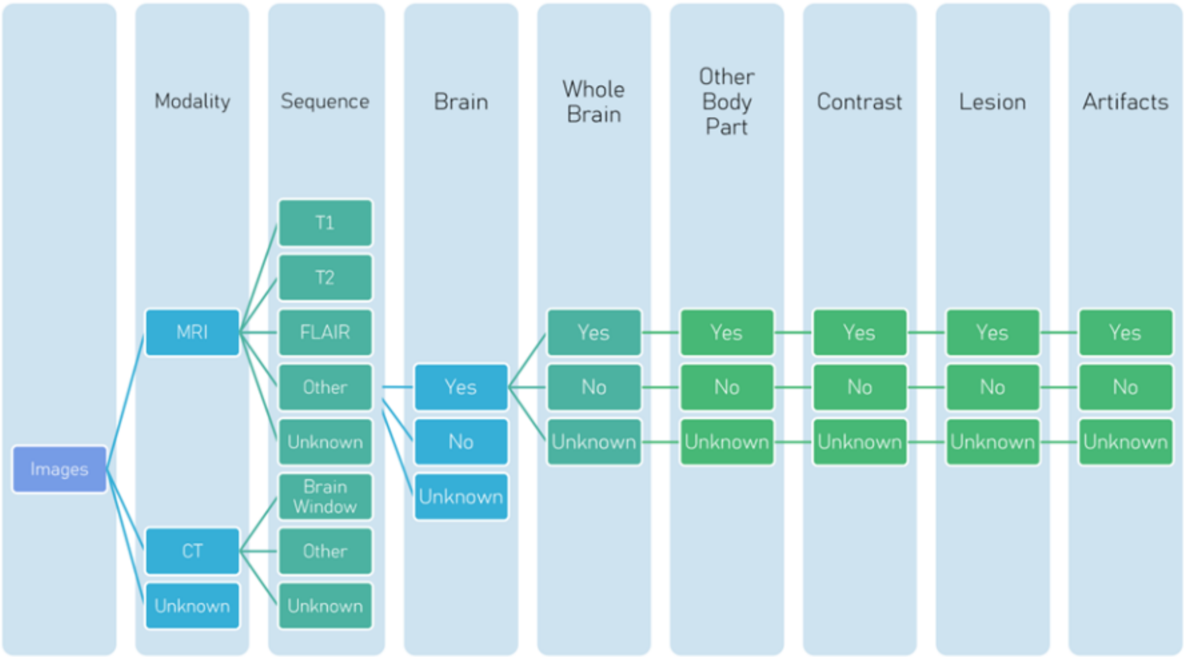

Figure 2: Criteria used by the annotators to label the test imaging set using the GUI developed.

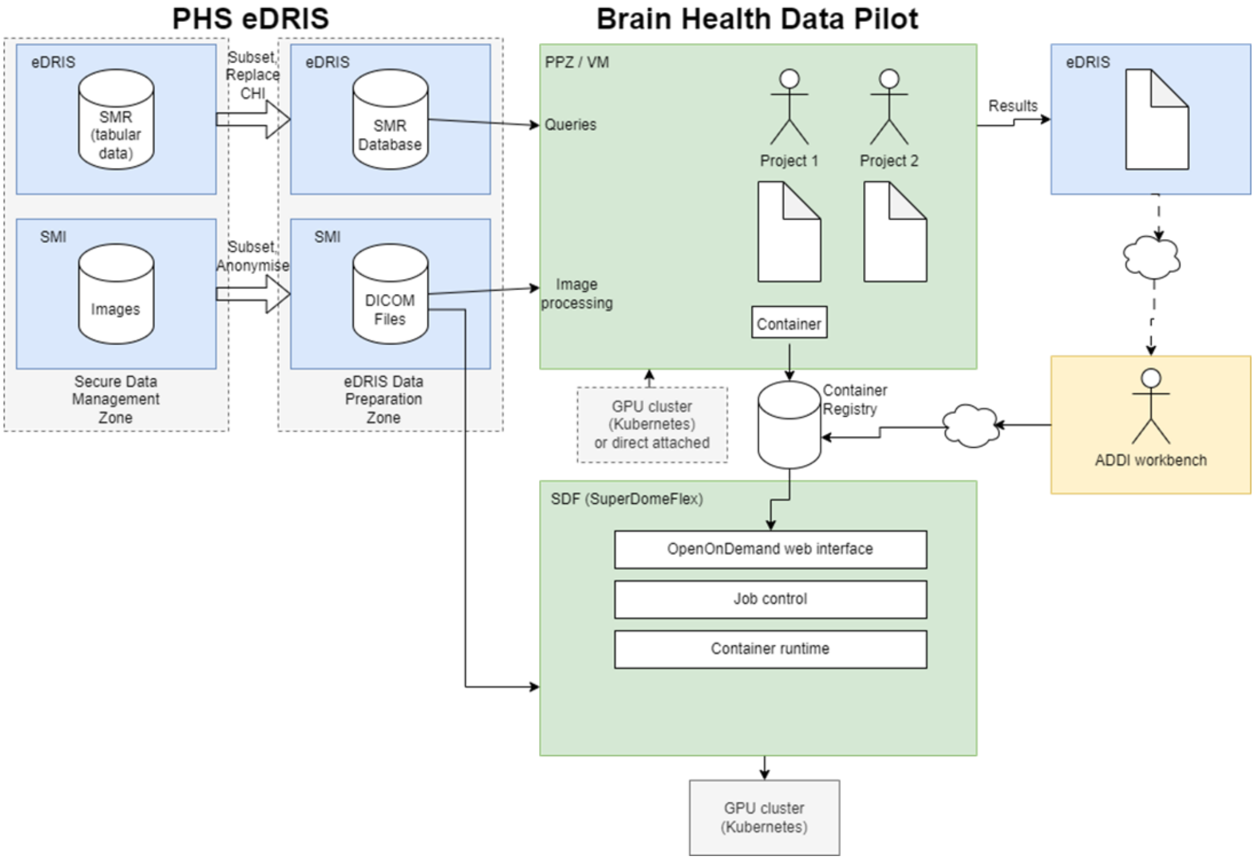

Figure 3: Diagram to illustrate the data flow and data linkage process in the Brain Health Data service (BHD). eDRIS Electronic Data Research and Innovation Service (eDRIS), SMR: Scottish Morbidity Record, CHI Community Health Index number, PPZ: privacy preserving zone, VM: virtual machine, DICOM: Digital Imaging and Communications in Medicine

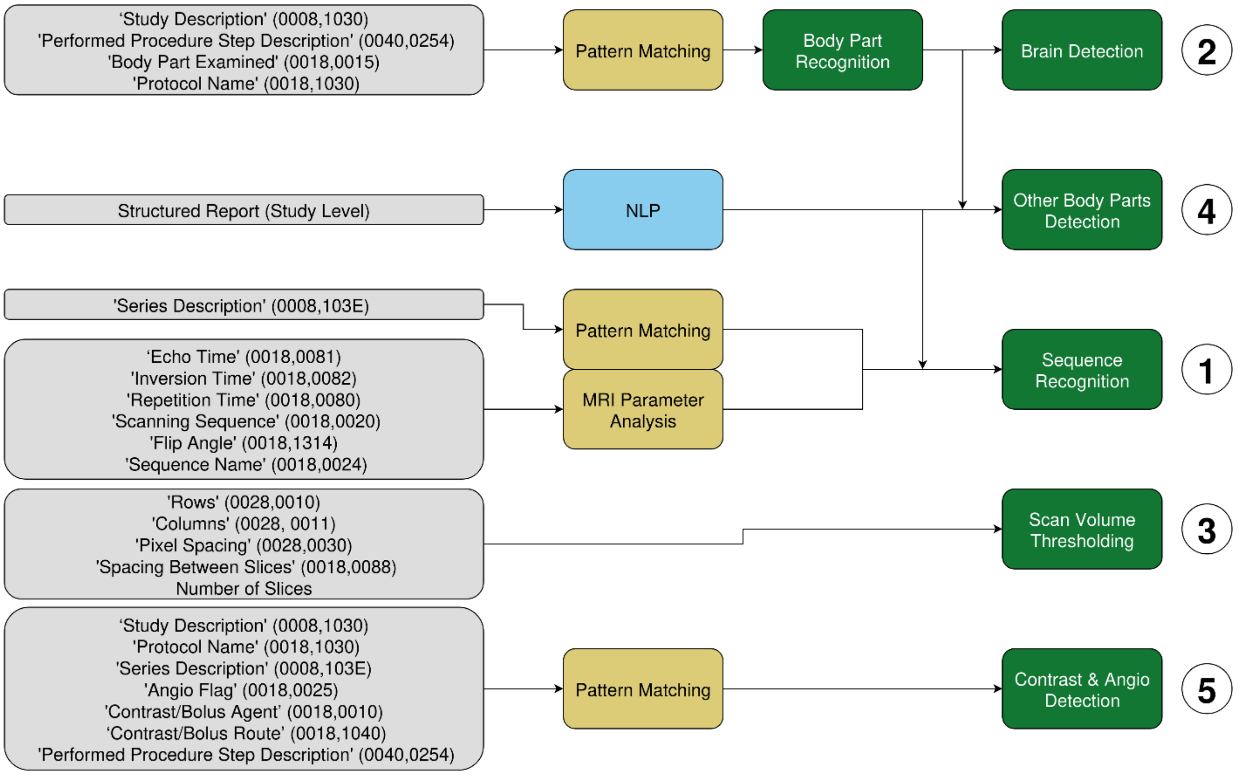

Figure 4: The exclusion process for the SCANDAN cohort. It is based on the automatic labelling (in yellow, including the pattern matching and the MRI parameter analysis) and the NLP of the radiological report (in blue). The steps were performed in the order from 1 to 5, numbered on the right. 1) Non T1/T2/FLAIR scans are excluded. 2) Scans without a brain are excluded. 3) Scans too small to contain a full brain are excluded. 4) Scans with other body parts (such as the spine) are excluded. 5) Contrast and angio scans are excluded.

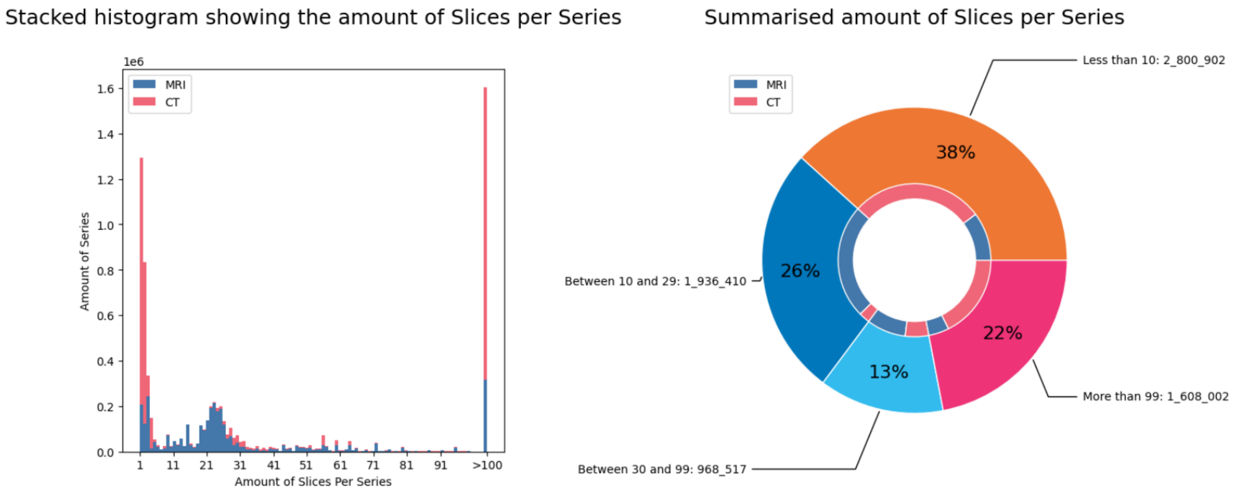

Figure 5: The distribution of slices per series within the data available through the BHD, for both MRI and CT. The stacked histogram shows the 2D MRI have a normal distribution of scans centered around 22, while the 3D MRI have over 100 slices. The CT have mostly more than slices. Both modalities have a large amount of localiser scans with under 10 slices.

A large dataset of brain imaging linked to health systems data: the curation and access to a whole system national cohort from NHS Scotland

Response to Reviewers

We thank the reviewers for the time they spent and their helpful comments. Both of them pointed out inconsistencies in the scope and objectives of the paper, and their insight was very valuable in reframing the narrative of this note to align with our original goals. We also thank the editor for their time they allocated to our paper.

Reviewer 1:

1. The abstract and introduction emphasize the full raw scale of the dataset (hundreds of thousands of MR/CT scans) and the curation framework, whereas the Results section focuses primarily on a derived dementia-matched cohort and a subset of retrieved studies. Please clearly separate these aspects and report, with explicit counts, the total number of raw imaging studies available in SMI (MRI and CT), as well as the number of unique patients.

answer: Agree - revised

- ABSTRACT: We clarified the abstract by adding the size (45K scans) of the SCANDAN cohort and the type of images used (MRI). We have made clear the data is from a subset of the BHD data (Abstract, par. 1).
- INTRODUCTION: We clarified the data available in the BHD (MRI, CT, electronic health record (EHR) and free text radiology report). We also clarified the distinction between the BHD data and the SCANDAN pipeline, introducing clearly the aims of both and their relationship (Introduction, par. 5).
- METHODS: We added a new subsection called "Data Availability" which lists the number of scans, report and EHR available through the BHD, as well as the number of patients. We included the count for different subset of data sources, such as the different EHR.
- FIGURE: We added Figure 5, which shows the distribution of slices per series, emphasising the scale of the dataset. (Methods, Data Availability)
- RESULTS: We changed the previous subsection "Dataset description" to "SCANDAN Output Dataset", to now reflect properly the distinction between the BHD data (the raw dataset) and the results of the curation framework.
- NARRATIVE: The addition of those two subsections, as well as the reorganisation of the introduction was done to resolve the first point of the reviewer here, highlighting

the difference in focus within the paper between the introduction and the results. To this end, we also renamed the Methods subsection relevant to SCANDAN by prefixing them with SCANDAN. Overall, our change aim to make sure the aim of the paper is clear; the introduction of the BHD, the presentation first pilot project carried with its data, and the additional data which it provided to the BHD

2. Please provide more detailed information on the subset of scans that successfully passed the pipeline filters (e.g., T1/T2/FLAIR sequences, whole-brain coverage, absence of contrast or angiography). Although Table 5 partially addresses this, its framing could be clearer—for example, please clarify whether the "Final" category corresponds to the number of research-ready MRI series per sequence.

answer: Agree – revised

- TABLE: We renamed the last column of table 5 from “final” to selected. We also expanded the caption to clarify the meaning of each column.
- RESULTS: We referenced Table 5 in the results, providing additional precision on the number of scans excluded by the label and the number of scans selected as the "first chronologically within the study" and listing the total amount of studies. (Results, SCANDAN Output Dataset, par. 3)

3. Please clarify what data are currently accessible to external researchers through eDRIS/BHD. For instance, does access include raw DICOM files, NIfTI conversions, derived labels, structured report phenotypes, or predefined example cohorts?

answer: Agree – revised

- METHODS: The “Data Availability” subsection lists all the data contained within the BHD
- RESULTS: A new section named "SCANDAN Contribution and BHD data" was added, explaining which data are available within the BHD (referencing the one listed in the "Data Availability" subsection), as well as listing the 5-output produced by SCANDAN. It describes each of these outputs, including their scope (whether it refers to the whole dataset, or the SCANDAN cohort). Each of these outputs are described in more details in the other Results subsection "SCANDAN Output Dataset".

4. The manuscript describes rule-based parsing of multiple DICOM tags using regular expressions, together with MRI parameter checks (e.g., TR/TE/TI), to define sequence labels. Please provide a complete list of the regular expression patterns used for sequence, body-

part, angiography, and contrast identification, along with information on versioning and how updates were handled across iterative annotation rounds.

answer: Partially Agree - revised

- A paragraph was added to clarify the creation of the regular expressions (based on prior in-house study, expanded to include variation from all the hospitals, then using the most common occurrence of each tag to make sure nothing is widely excluded, before finally using the manual annotations to refine them). (Methods, SCANDAN: Identification of MRI scan type and sequence, par. 5)
- We provided a link to a GitHub repository containing the regular expression (Methods, SCANDAN: Identification of MRI scan type and sequence, par. 2)

5. Please clarify how the criterion "3D image volume > 5 litres" is computed and provide a rationale for the choice of this threshold.

answer: Agree – revised

- We clarified how the threshold was chosen based on empirical test, from a previous study using similar data. We compared the volume to the annotations to assert it was correct. We added a citation to it (Singh Reel et al). (Methods, SCANDAN: Identification of MRI scan type and sequence, par. 1).

6. The expert annotation study (n = 713 scans across MRI and CT, with 100 overlapping cases and GLAD adjudication) is a strong component of the work. Please clarify how these 713 scans were sampled (e.g., randomly across sites, scanners, and years; stratified by modality; or enriched for challenging or ambiguous cases).

answer: Agree - revised

- We clarified that the data was randomly sampled by EPCC to be provided as example data (Methods, SCANDAN: Data validation and quality control, par. 2)

7. The manuscript currently lacks a concise summary of key imaging metadata. For neuroimaging researchers, it would be highly valuable to include aggregate summaries of:
(1) scanner vendors/models and magnetic field strengths (for MRI), and their distribution across sites and over time;
(2) typical voxel sizes, slice thickness distributions, and acquisition planes for the retained sequences; and
(3) rates of partial brain coverage or localizer scans.

answer: Partially Agree - revised

- We added the number of localiser (along other labels) (Results, SCANDAN Output Dataset, par 3.)
- We added the amount of hospitals site, MRI devices and vendors contained within the data delivered to SCANDAN (21K studies MRI). (Results, SCANDAN Output Dataset, par 4.)
- As a proxy to slice thickness/voxel sizes, we added the distribution of slices per series within Figure 5. This data is obtained on the whole data from the BHD, including CT
- The information asked could not be provided on the entire scale of the BHD for some case due to the metadata not being available. (Methods, Data Availability)

8. Please indicate whether defacing is performed or feasible within the Trusted Research Environment for MR T1-weighted volumes, and whether defacing is mandatory for any form of data export (recognizing that raw images may not be exported, but derived images could be generated).

answer: Partially Agree – partial revision

- No defacing was performed.
- No unconsented images can be exported from the secure data environment.
- The discussion already provides a paragraph on privacy concern (Discussion, par 5.). It also includes two mentions that any data taken out of the Trusted Research Environment need to be validated by PHS (Discussion, par. 2 & par. 5).
- We added to the discussion the mention that PHS review the data to "excludes any identifiable data." (Discussion, par 5).

9. The manuscript alternates between the terms "scan," "study," "series," and "image." Although these are defined earlier, some later statements could be clarified to avoid ambiguity. For example, the phrase "each study (i.e., one per person)" could be interpreted as implying that each individual has only one study, whereas later sections refer to selecting the "earliest studies associated with these subjects." Please clarify whether "one per person" reflects a deliberate selection criterion or an inherent property of the source data.

answer: Agree - revised

- We clarified how the linkage was done by eDRIS and EPCC (not us) and how it linked the study (scans) to "one person" (i.e. a patient) from the EHR (Methods, Data Sources, par. 1)

Reviewer 2:

1. Several figures aim to provide an overview of the data curation and processing pipeline, but the relationship between the overall SCANDAN project, the specific scope of this Data Note, and the individual curation steps described in the Methods is not always clear. In particular, Figure 1 illustrates the work packages of the overall SCANDAN project; however, it is not immediately clear how these relate to the specific focus of this manuscript. While SCANDAN aims to develop and evaluate dementia classifiers, this paper appears to focus primarily on the data curation and preparation framework. I encourage the authors to clarify in the text and figure legend which components of Figure 1 are directly covered in this manuscript, and which are shown for broader project context.

answer: Agree – revised

- ABSTRACT: we clarified the data curation pipeline was linked to the SCANDAN project (Abstract, par. 1), and was processing a subset of the BHD MRI data, adding the details on the relationship between the two projects (Abstract par. 1).
- INTRODUCTION: We reformulated the definition of the BHD, by focusing on the data it provides. We then explained SCANDAN, a project aiming to perform dementia classification, was the first to use the BHD data. We further explained that SCANDAN produced several outputs when processing the data which were added to the BHD, such as parsing of the radiological text report with NLP, or the dementia phenotyping. Finally, we summarised the relevant part of SCANDAN which are discussed in this paper, aiming to create a research ready dataset, demonstrating what is available within the BHD (Introduction par. 5).
- METHODS: We added a new subsection "SCANDAN Project", referencing Figure 1, which replace the previous explanation from the introduction, and explains in detail how each part interact together, clarifying the SCANDAN project (Methods, SCANDAN project)
- RESULTS: We added a new subsection "SCANDAN Contribution and BHD data" showing the relationship between SCANDAN output and the BHD. The SCANDAN output will be available through the BHD for other researcher, significantly speeding up their research by saving them the time to carry the same, complex, data curation and processing (Results, SCANDAN Contribution and BHD data).
- NARRATIVE: The addition of these 2 subsections, as well as the reorganisation of the introduction was done to address the reviewer point on the confusing relationship between SCANDAN and the BHD, highlighting the goal and scope of the paper were not clear. The BHD provides access to data and easier governance, and the SCANDAN project uses this data, demonstrating its usefulness, providing important demographic information on the dataset, and provided several key outputs which were added to the BHD. We hope those changes clarify the interlinked relationship the BHD and SCANDAN have.

2. Multiple data selection and refinement steps are described across the manuscript, including DICOM-based filtering, NLP-driven exclusions, manual annotation, and iterative re-annotation, but their sequencing and respective roles can be difficult to follow. Figure 4 appears to attempt to bring these elements together, yet it is not explicitly referenced in the text, and its legend lacks sufficient explanation to map the visual elements (including the numbered markers) to the corresponding methodological steps. More explicit cross-referencing between text and figures, and a concise summary (e.g. a short paragraph, table, or expanded figure legend) outlining the full sequence of automated and manual steps, would help clarify how each contributes to the final curated dataset.

answer: Agree – revised

- FIGURE: Figure 4 was referenced within the text (Methods, SCANDAN: Identification of MRI scan type and sequence, par. 1). Its caption was extensively rewritten to explain what each colour means, what each block does, and in which order they were performed. (Figure 4, Methods, SCANDAN: Identification of MRI scan type and sequence).
- METHODS: the new subsection "SCANDAN Project" also clarified the order in which the SCANDAN work package were used and how they interacted together, making it clearer what was done and when (Methods, SCANDAN Project).
- RESULTS: The subsection "SCANDAN Output Dataset" replace the previous "Dataset description" and provides a detailed description of the exclusion steps, with input and output numbers, referencing the summary Tables (3, 4, 5) which list the information more concisely (Results, SCANDAN Output Dataset)
- NARRATIVE: We reorganised the Methods, moving "SCANDAN: Identification of MRI scan type and sequence" after "SCANDAN: Data validation and quality control". We also clarified which parts were linked to SCANDAN by prefixing the subsection title with "SCANDAN".

3. Data sources section: the description of data linkage and processing (e.g. "We linked each study…", "All data was processed and stored…") would benefit from clarification on which steps are automated and which require manual intervention.

answer: Disagree – minor revision for clarification

- We clarified how the linkage was done by eDRIS and EPCC (not us) and how it linked the study (scans) to "one person" (i.e. a patient) from the EHR (Methods, Data Sources, par. 1)
- The storage, just like the linkage, is performed by ePCC and is inherent to the National Safe Haven. If the reviewer is referring to the rest of the processing

described in the Methods, we believe each relevant section highlight the steps were done automatically, save for the annotation.

4. NLP tool: it would be useful to clarify whether the NLP tool is openly available (e.g. as code or a container), as this could be of significant value to the community. In addition, the sentence on page 6 describing the "enhancement of the detection of section boundaries" is highly technical and difficult to follow in this context; a brief rephrasing or explanation of what is meant by section boundaries would help. More generally, a short high-level description of the NLP inputs, outputs, and how these outputs are used within the broader framework would improve readability.

answer: Agree – revised

- We added a citation with a link to EdIE-R (Methods, SCANDAN: Natural language processing of brain imaging reports, par. 4).
- We re-phrased the sentence about section boundaries to simplify it, explaining we refer to the boundary between the report and the clinical history (Methods, SCANDAN: Natural language processing of brain imaging reports, par. 3).
- We provided an additional paragraph summarising the NLP methodology to select the radiological report used as input and their filtering (Methods, SCANDAN: Natural language processing of brain imaging reports, par. 5).
- We also provided a summary of the NLP report output in the Results, listing some of the finding, as well as the numbers of report processed (Results, SCANDAN Output Dataset, par. 2)

5. Phenotyping dementia: this section appears to be missing a reference to Table 1. In addition, Table 1 would benefit from a more informative legend explaining how it should be interpreted. For example, it is unclear why a BNF code is reported only for Alzheimer's disease. While the use of wildcards is understandable, the rationale and intended use of these wildcards should be explicitly stated in the table legend.

answer: Agree – revised

- We added two references to support out method for phenotyping dementia (Doney et al (2019), Doney et al (2025)). We also clarified why the BNF code is only used for Alzheimer's as it's the only that have specific medication. (Methods, SCANDAN: Phenotyping Dementia)
- We added the reference to Doney et al (2025) to the caption of Table 1, and clarified the meaning of the wildcards as well as the reason for their usages, i.e., to that all child-codes in the hierarchy are included (Table 1, Methods, SCANDAN: Phenotyping Dementia)

- The reference to Table 1 was fixed.

6. Cohort building: the description of cohort selection (e.g. "We first selected… gone through the pipeline… and had no NLP label") would benefit from clearer specification of the order of steps, and of which steps are automated versus manual. Explicit input/output descriptions and a reference to a visual flowchart would help clarify this process.

answer: Partially agree – revised

- The order of the steps for the cohort building was clarified, by removing some steps happening later, which were introducing confusion by being mentioned this early. Specifically, we removed the mention to the image filtering based on the DICOM tag. We kept the exclusion using NLP mention of tumour or strokes. (Methods, SCANDAN: Cohort Building, par. 2)
- The cohort building input, output and the number of excluded samples, referenced in Table 3, were added to the results (Results, SCANDAN: Data).
- We did not add another flowchart. However, we expect the rest of the modification on the paper, about signposting, cross-referencing, and clarification of the order of the SCANDAN project, will clarify the order in which each step was done. None of these steps were "manual".

7. DICOM tags: similarly, the description of how DICOM-derived labels are combined with NLP outputs would benefit from clearer sequencing and explicit reference to a flowchart illustrating the full pipeline.

answer: Agree - revised

- A reference to Figure 4 has been added (Methods, SCANDAN: Identification of MRI scan type and sequence, par. 1). The caption of Figure 4 has been improved to explain the sources of the data, illustrating exactly which tags are used for which task, through which methods. It also shows the combination of the NLP and the pattern matching methods using DICOM (Figure 4, Methods, SCANDAN: Identification of MRI scan type and sequence).
- We added a sentence about how the NLP is used to validate the presence of a sequence within a study (Methods, SCANDAN: Identification of MRI scan type and sequence, par. 4).

8. Data validation and quality control: Table 2 appears to relate to this section but is only explicitly referenced in the Results section. It is also unclear what components are included in the "automated labelling process described above" (e.g. DICOM-based rules only, NLP-

derived information, or both). Clarification would also be helpful on what constitutes the "ground truth" used for this comparison, in particular whether the results in Table 2 reflect the final labels after all rounds of annotation, GLAD aggregation, and expert adjudication.

answer: Disagree – partially revised anyway

- Table 2 is only explicitly referenced in the results section as it shows the results of the Data validation and quality control. We clarified in the methods that we are reporting in the results the final comparison of the labelling and the annotation (Methods, SCANDAN: Data validation and quality control, par. 3).
- The automatic label refers the output of the tools described in the section "SCANDAN: Identification of MRI scan type and sequence" (now "next section" previously "above"). There are one group of labels described in this section, described in paragraph 1, with the process to obtain them described in paragraph 1 to 3. Those labels described match the one which were annotated. We hope the clarification concerning NLP usage and the expanded caption for Figure 4, now properly referenced in the relevant section, will explain that the label compared are the output of the agglomeration of those.
- We clarified the caption of Table 2 to reflect that the manual annotation is used as ground truth to assess the labelled obtained automatically (Table 2, Results, SCANDAN Output Dataset)
- We also added clarification regarding the comparison between the automatic tools and the manual annotations using the final versions (Methods, SCANDAN: Data validation and quality control, par 3.; Results, SCANDAN: Data validation and quality control, par 7.)

9. Use of GLAD: the role of GLAD in the workflow would benefit from further clarification. Specifically, it would be helpful to briefly state what inputs are provided to GLAD, what outputs are produced, and how these outputs are used in the evaluation of the automated labelling. It is currently unclear whether the results in Table 2 are based on GLAD-inferred labels or on subsequent manually adjudicated labels.

answer: Agree – revised

- We updated Table 2 caption to reflect that the ground truth is the manual annotation (Table 2, Results, SCANDAN: Data validation and quality control)
- We updated the explanation of GLAD usage as it had been previously erroneous. GLAD was not used to infer automatic label but used on the automatic label. It used the overlapping 100 annotations as "ground truth" and corrected the rest of the annotation (Methods, SCANDAN: Data validation and quality control, par. 2 & 3).

- We hope the clarification of how GLAD was used will answer the reviewer comment and may clarify some of their other comments. We thank the reviewer for pointing out the confusion.

10. Results of data validation and quality control: again, clarification on what is included under "automated tools" would help ensure consistency with the workflow described in the Methods. The authors might also consider illustrating the iterative annotation and adjudication process in a dedicated flowchart.

answer: Disagree – partially revised anyway

- We added a mention of Figure 4 in the results section to ensure the automatic tools and labels are referenced (Results, SCANDAN: Data validation and quality control).
- We added a sentence mentioning how we report the final comparison between the automatic tools and the label (Methods, SCANDAN: Data validation and quality control, par. 3)

11. Discussion: the authors note that the dataset will continue to expand in size; a brief summary of future plans for cohort growth and linkage would be of interest.

answer: Agree - revised

- We expanded on the plan to add NIfti and other processed images available to other project, in a similar fashion to what SCANDAN provided with the metadata and table output (Discussion, par. 7)
- We also clarified that one of the output of SCANDAN, the sequence/body/contrast/... labelling, was carried only on the 21K study from the SCANDAN sub cohort, but are planned to be expanded to the rest of the data in the BHD (Results, SCANDAN Contribution and BHD data)

12. Phenotyping dementia - a reference appears to be missing.

answer: Agree - revised

- Reference to table 1 fixed

13. Figure 3 legend: consider expanding it to include the explanation of colours and acronyms currently provided only in the text.

answer: Agree - revised

- Figure 3 legend now updated to: Figure 3. Diagram to illustrate the data flow and data linkage process in the Brain Health Data service (BHD). eDRIS Electronic Data Research and Innovation Service (eDRIS), SMR: Scottish Morbidity Record, CHI Community Health Index number, PPZ: privacy preserving zone, VM: virtual machine, DICOM: Digital Imaging and Communications in Medicine

14. Page 11: "sizer" appears to be a typo and should read "size".

answer: Agree - revised
