# Supplementary material for: A large dataset of brain imaging linked to health systems data: curation and access to a whole system national cohort from NHS Scotland
Source: Gigascience. 2026 Jun 9;15:giag072. doi: 10.1093/gigascience/giag072 (PMC13347094; doi:10.1093/gigascience/giag072)
Supplement: giag072_GIGA-D-25-00442_revision_2 [file giag072_giga-d-25-00442_revision_2.pdf]

GigaScience

A large dataset of brain imaging linked to health systems data: curation and access to a whole system national cohort from NHS Scotland

--Manuscript Draft--

**Manuscript Number:**	GIGA-D-25-00442R2
**Full Title:**	A large dataset of brain imaging linked to health systems data: curation and access to a whole system national cohort from NHS Scotland
**Article Type:**	Data Note
**Funding Information:**	Eisai — Dr Michael P J Camilleri<br>LifeArc — Dr Michael P J Camilleri<br>Gates Ventures — Dr Michael P J Camilleri<br>HDRUK — Dr Michael P J Camilleri<br>Alzheimer's Disease Data Initiative — Dr Michael P J Camilleri
**Abstract:**	We present the design and implementation of a data curation framework to generate a large-scale clinical brain imaging dataset suitable for artificial intelligence (AI) enabled image analysis. The dataset is accessible through the Brain Health Data (BHD) initiative, which includes approximately 417,341 magnetic resonance imaging (MRI) and 846,077 computerized tomography (CT) head studies, linked electronic health records (EHRs), and associated free-text imaging reports from clinical practice between 2010 and 2018 in Scotland, exceeding 185 TB in size. The data curation framework was developed during the SCottish AI in Neuroimaging to predict Dementia and Neurodegenerative Disease (SCANDAN) study, which used a subset of 41,966 MRI series from the BHD for dementia prediction.<br>We describe the processing of the BHD metadata and our multilabel classification output. We discuss the strengths of the BHD, including clinical relevance thanks to its unprecedented scale, population-wide representativeness of a national free-at-the-point-of-delivery healthcare, long-term follow-up to neurodegenerative disease, and real-world variability. We describe the challenges and lessons learnt in developing a framework to curate data, including the time needed to obtain permissions, the need for easily accessible, secure, responsive and affordable computational environments, the variability of clinical data, and the challenge of extracting linked clinical data and images at scale.<br>This resource will be crucial for clinical research, fostering the development of personalized medicine approaches, and fast-tracking the implementation of AI models in clinical workflows. We encourage the use of the BHD data through a streamlined application to the Public Benefit and Privacy Panel for Health and Care via the Data Research and Innovation Service of Public Health Scotland (eDRIS).
**Corresponding Author:**	William Whiteley<br>The University of Edinburgh Edinburgh Neuroscience<br>University of Edinburgh, UNITED KINGDOM
**Corresponding Author Secondary Information:**	
**Corresponding Author's Institution:**	The University of Edinburgh Edinburgh Neuroscience
**Corresponding Author's Secondary Institution:**	
**First Author:**	Michael P J Camilleri
**First Author Secondary Information:**	
**Order of Authors:**	Michael P J Camilleri<br>Dorian Gouzou<br>Salim Al-Wasity

Powered by Editorial Manager® and ProduXion Manager® from Aries Systems Corporation

	Muthu R K Mookiah
	María Valdes Hernandez
	Bea Alex
	Sotirios A. Tsaftaris
	Andrew Brooks
	Ruairidh MacLeod
	Honghan Wu
	Brenda Bauer
	Claire Grover
	Parminder Reel
	Susan Krueger
	Richard Tobin
	J. Douglas Steele
	Grant Mair
	Joanna Wardlaw
	Alexander Doney
	Emanuele Trucco
	William Whiteley
**Order of Authors Secondary Information:**	
**Response to Reviewers:**	Response to Reviewers<br>We are grateful to the editor and the reviewers for carefully reviewing out manuscript and making suggestions for improvement.<br><br>Reviewer 1:<br><br>1. The description of dataset scale still remains largely inconsistent and potentially confusing. The manuscript reports quantities at different hierarchical levels (e.g., "417,000 MRI", "3.37 million series", "409 million slices"), but does not consistently clarify whether counts refer to studies, series, slices, or unique individuals. For example, it is not always clear whether "417,000 MRI" refers to studies or patients, which represents fundamentally different dataset scales. A unified and explicit reporting of counts at each level (patients, studies, series, slices) is still needed to make the dataset interpretable and comparable.<br><br>answer: Minor revision<br><br><br>We have endeavoured to clarify these uncertainties with the following edits:<br><br>-We replaced every mention of “scans” when used with a number to avoid any ambiguity.<br>-We also added the detail of the MRI and CT slices split (130.3 million MRI and 279.7 million CT) (Methods, Data availability)<br>-However, we have been very clear in other sections. The Data Availability subsection mentions: 830K patients, 417K MRI studies, 846 CT studies, 3.37 million MRI series, 3.15 million CT series, and 410 million slices (now with 130.3 million MRI and 279.7 million CT). Each hierarchical level was mentioned and detailed. The unified and explicit reporting of counts at each level the reviewer is asking was already provided.<br><br><br>2. The characterization of imaging acquisition remains insufficient for downstream use. While the revision adds some information on scanner vendors and field strengths, the

manuscript still lacks structured summaries of acquisition variability (e.g., per-site/per-scanner distributions, sequence-specific counts, voxel size, slice thickness, acquisition planes). Without these details, it is difficult to assess heterogeneity or design harmonization strategies, which limits the practical value of the dataset for neuroimaging research.

answer: Revision

-We have already provided a sequence count for the MRI datareceived by SCANDAN.
-Voxel size/row & columns counts/acquisition plane were not provided as metadata for the whole dataset, and only obtained through the DICOM slices, which for SCANDAN were limited to the 21K MRI studies received. This also limits the sequence count to the SCANDAN data, as it as not yet been carried on the rest of the data.
-Regardless of this, the dataset contains thousands of different acquisition combinations, and such table would be so large that, even if it was authorised to be extracted from the national safe haven, would be difficult to provide in an interpretable format. We understand the request but do not believe it is adapted to a dataset of this size, or from clinical origin.
-We added Table 1 with the amount of unique value for some key metadata: Protocol Name, Series Description, Performed Procedure Step Description, Body Part Examined and Institution Name. We also provided the number of series which had an empty field for these values, which should provide a measure of the heterogeneity of the dataset, as the reviewer explained it was difficult to assess.
-We aim to continue improving the labelling of the scans in future work to provide better characterisation for new users of the data.

3. The quality control strategy is not described at a level expected for large-scale neuroimaging resources. The current approach focuses on rule-based filtering (DICOM tags, NLP exclusions) and manual annotation, but does not include quantitative image quality metrics (e.g., SNR, motion, artifacts) or standardized QC frameworks (e.g., MRIQC-like measures). It is also unclear what proportion of images were excluded due to poor quality versus metadata-based criteria. Without quantitative QC reporting, the usability of the retained dataset remains difficult to evaluate.

answer: No revision When working with images at very large scale, computation is a limiting factor for running standardised QC metrics. We therefore used more computationally efficient methods based on meta-data. We will continue to develop this dataset, and it is an openly available resource for researchers to test these methods in smaller subsets of the data. As we are unable currently to provide the quantitative QC metrics we have not provided these.

4. The manuscript would benefit from a clearer demonstration of the dataset's analytical value. Although this is presented as a Data Note, the current outputs are primarily structural (sequence labeling, report parsing, cohort construction), and there is limited illustration of how the curated data enable downstream scientific or clinical analyses. A more concrete example (even exploratory) of how the dataset can be used for a neuroimaging or clinical research question would strengthen the justification of its utility.

answer: No revisionWe have completed analyses in the data that we willo publish soon, and others are ongoing. The primary purpose of the paper is to present the dataset, and we believe that including analyses would risk lead to a very long paper that would lack focus.

Reviewer 2:

1. The tables are not presented and mentioned in order of appearance (table 3 is mentioned before table 2, table 5 before table 4). Please change to enable a coherent flow of information.

answer: Revision

	We edited the numeration of table as well as their order (3>2, 5>3, 2>5) (incremented by one as we added a new table as Table 1.). We also update the Figure numeration (5>1, 1>2, 2>3, 3>5).<br><br>2. Table 5 is still unclear to me. From the new legend, the headings of the columns should represent an excluding step, but the fact that it is a brain image or a whole brain image shouldn't be an inclusion step? In other words the 60 series excluded from the "Brain" column, were because they were ore were not containing brain images? I suggest adding a legend or some explanation about what each excluding step means.<br><br>answer: Revision<br><br>We clarified the legends to emphasise it was indeed exclusion steps. We renamed column which hinted as inclusions step to make clear they were exclusion steps by highlighting what was removed. (Original > Baseline, Brain > Brain Absent, Whole Brain > Partial Brain, Other Body Parts > Other Body Parts Present, Contrast & Angio > Contrast or Angio Present).
**Additional Information:**	
**Question**	**Response**
Are you submitting this manuscript to a special series or article collection?	No
**Experimental design and statistics**<br><br>Full details of the experimental design and statistical methods used should be given in the Methods section, as detailed in our Minimum Standards Reporting Checklist. Information essential to interpreting the data presented should be made available in the figure legends.<br><br>Have you included all the information requested in your manuscript?	Yes
**Resources**<br><br>A description of all resources used, including antibodies, cell lines, animals and software tools, with enough information to allow them to be uniquely identified, should be included in the Methods section. Authors are strongly encouraged to cite Research Resource Identifiers (RRIDs) for antibodies, model organisms and tools, where possible.<br><br>Have you included the information requested as detailed in our Minimum	Yes

Powered by Editorial Manager® and ProduXion Manager® from Aries Systems Corporation

Standards Reporting Checklist?	
**Availability of data and materials**<br><br>All datasets and code on which the conclusions of the paper rely must be either included in your submission or deposited in publicly available repositories (where available and ethically appropriate), referencing such data using a unique identifier in the references and in the "Availability of Data and Materials" section of your manuscript.<br><br>Have you have met the above requirement as detailed in our Minimum Standards Reporting Checklist?	Yes
GigaScience has policies and guidelines in place for the use of generative AI-writing tools such as ChatGPT. If you have used such writing tools to assist with writing the manuscript this must be declared and cited in the text. Authors should not list AI-writing tools and other AI-assisted technologies as an author or co-author and should acknowledge that they are fully responsible for text generated or refined by AI-writing tools.<p><br><br>A summary of use (particularly in the introduction or among methods) needs to be included at the end of the paper, and the outputs should also be included as a supplementary file hosted in GigaDB or other open repositories. Please read our guidelines for more information. <p><br><br>By submitting to GigaScience, you are aware of the journal's AI-writing tools policy, and if you have declared use of such tools below, you have acknowledged this where appropriate in your manuscript and have made a summary of use and outputs available. <p><br><b>AI-assisted writing tools have been used in the preparation of this manuscript?	No

Powered by Editorial Manager® and ProduXion Manager® from Aries Systems Corporation

Manuscript
Click here to access/download;Manuscript;Methodology paper v11_clean.docx

A large dataset of brain imaging linked to health systems data: curation and access to a whole system national cohort from NHS Scotland

Michael P J Camilleri[1,2]*

Dorian Gouzou[3]*

Salim Al-Wasity[4]

Muthu R K Mookiah[4]

María Valdes Hernandez[3]

Bea Alex[5]

Sotirios A. Tsaftaris[2]

Andrew Brooks[6]

Ruairidh MacLeod[6]

Honghan Wu[7,8]

Brenda Bauer[3]

Claire Grover[5]

Parminder Reel[9]

Susan Krueger[9]

Richard Tobin[5]

J. Douglas Steele[4]

Grant Mair[3]

Joanna Wardlaw[3,10]

Alexander Doney[11]

Emanuele Trucco[1]**

William Whiteley[3,7,12]**

ORCIDs:

- Emanuele Trucco– 0000-0002-5055-0794
- Andrew Brooks – 0000-0002-8837-3201
- Dorian Gouzou – 0000-0003-4865-7784
- Richard Tobin – 0000-0003-4050-7888
- Brenda S Bauer – 0000-0002-1417-1260

- Maria Valdes Hernandez – 0000-0003-2771-6546
- Douglas Steele– 0000-0002-9822-8753
- Ruairidh MacLeod – 0000-0002-2641-2543
- Muthu Rama Krishnan Mookiah – 0000-0001-6437-1482
- Honghan Wu – 0000-0002-0213-5668
- Joanna Wardlaw - 0000-0002-9812-6642
- Michael Camilleri - 0000-0002-8619-3231
- Salim Al-Wasity - 0000-0002-6762-8324
- Claire Grover - 0000-0001-7019-1250
- Susan Krueger - 0000-0002-5219-1959
- Grant Mair - 0000-0003-2189-443X
- Alexander Doney - 0000-0002-6210-5620
- Sotirios A. Tsaftaris - 0000-0002-8795-9294
- Beatrice Alex - 0000-0002-7279-1476

[1] Computing, School of Science and Engineering, University of Dundee, Dundee, UK.

[2] School of Engineering, University of Edinburgh, Edinburgh, UK.

[3] Institute for Neuroscience and Cardiovascular Research, School of Medicine, University of Edinburgh, UK.

[4] School of Medicine, Ninewells NHS and University Hospital, Dundee, UK.

[5] School of Informatics, University of Edinburgh, Edinburgh, UK.

[6] Edinburgh Parallel Computing Centre, University of Edinburgh, Edinburgh, UK.

[7] Usher Institute, School of Medicine, University of Edinburgh, Edinburgh, UK.

[8] School of Health and Wellbeing, University of Glasgow, Glasgow, UK.

[9] Health Informatics Centre, School of Medicine, University of Dundee, Dundee, UK.

[10] UK Dementia Research Institute Centre at the University of Edinburgh

[11] Cardiovascular Research, School of Medicine, University of Dundee, Dundee, UK.

[12] Health Data Research UK, London, UK

* equal contribution

** equal contribution

Correspondence: william.whiteley@ed.ac.uk

ABSTRACT

We present the design and implementation of a data curation framework to generate a large-scale clinical brain imaging dataset suitable for artificial intelligence (AI) enabled image analysis. The dataset is accessible through the Brain Health Data (BHD) initiative, which includes approximately 417,341 magnetic resonance imaging (MRI) and 846,077 computerized tomography (CT) head studies, linked electronic health records (EHRs), and associated free-text imaging reports from clinical practice between 2010 and 2018 in Scotland, exceeding 185 TB in size. The data curation framework was developed during the SCottish AI in Neuroimaging to predict Dementia and Neurodegenerative Disease (SCANDAN) study, which used a subset of 41,966 MRI series from the BHD for dementia prediction.

We describe the processing of the BHD metadata and our multilabel classification output. We discuss the strengths of the BHD, including clinical relevance thanks to its unprecedented scale, population-wide representativeness of a national free-at-the-point-of-delivery healthcare, long-term follow-up to neurodegenerative disease, and real-world variability. We describe the challenges and lessons learnt in developing a framework to curate data, including the time needed to obtain permissions, the need for easily accessible, secure, responsive and affordable computational environments, the variability of clinical data, and the challenge of extracting linked clinical data and images at scale.

This resource will be crucial for clinical research, fostering the development of personalized medicine approaches, and fast-tracking the implementation of AI models in clinical workflows. We encourage the use of the BHD data through a streamlined application to the Public Benefit and Privacy Panel for Health and Care via the Data Research and Innovation Service of Public Health Scotland (eDRIS).

INTRODUCTION

Brain imaging plays a crucial role in the diagnosis of neurological disorders. However, clinical imaging services are under great demand, highlighting the need for new tools to improve radiology workflows. These tools should accelerate image assessment, reduce the workload for radiologists, and ultimately improve patient care. Artificial intelligence (AI) methods show promise for faster diagnosis for example in acute ischaemic stroke [1] and similar improvements are possible in head injury, neurodegeneration, dementia and brain cancers[2,3]. To develop and test AI models that are clinically relevant, researchers need access to large datasets of clinically acquired images, and secure, ethical data provision. Such large datasets, however, are complex to process, due to the computational bottlenecks of several open-source software suites.[4] Hence, secure environments should also provide sufficient computing resources.

A survey conducted between December 2024 and February 2025 across UK secure data environments revealed the lack of brain imaging resources with nationwide coverage. For instance, the Diagnostic Imaging Dataset curated by NHS England includes patient-level metadata on the 501 million diagnostic imaging procedures performed in NHS England since April 2012, but it lacks imaging data and associated reports.[5]

The preparation of large repositories of routinely collected imaging data is challenging, particularly in privacy-protecting secure data environments. Despite adherence to Digital Imaging and Communications in Medicine (DICOM)[6] standards, real-world medical imaging datasets vary significantly in quality, format, and acquisition protocols, which makes standardisation across different imaging sources necessary. Determining imaging sequences (e.g., T1- or T2-weighted magnetic resonance imaging (MRI)) is essential for analysis but can be difficult in practice. DICOM meta-data tags provide rapid but sometimes unreliable classification, while image-based classification is more accurate but computationally demanding, and not free from uncertainty. [7,8] Natural language processing (NLP) of radiology reports can facilitate sequence identification and filter out scans with artefacts or missing structures. However, automated image quality assessment is paramount. Pre-processing and data retrieval is easier with automation of pipelines and integration of structured clinical records.

Compliance with governance frameworks is important to access large-scale unconsented clinical imaging datasets within safe havens, and needs ethical approval, data governance approval, and compliance with privacy regulations, all with costs. These administrative barriers, although necessary, can significantly delay or completely deter research.

To address these challenges, we developed the Brain Health Data (BHD) framework, which unifies all clinical brain imaging data acquired in Scotland with linked clinical information, to facilitate access to data through the Electronic Data Research and Innovation Service (eDRIS) of Public Health Scotland (PHS).[9] The data within the BHD framework includes approximately 417,341 MRI and 846,077 computerized tomography (CT) head studies, linked electronic health records (EHRs), and free text radiology reports collected between 2010 and 2018. It offers clinical relevance with its unprecedented scale, population-wide representativeness of healthcare, long-term follow-up to neurodegenerative disease, and real-world variability. To

curate this large-scale clinical brain imaging dataset in a format suitable for AI analysis we designed and implemented a data processing pipeline within the SCottish AI in Neuroimaging to predict Dementia and Neurodegenerative Disease (SCANDAN) study, which aimed to develop AI algorithms for reliable dementia risk estimation from routine brain imaging and clinical records. This paper describes SCANDAN's methods and outputs, highlights key lessons learned from working within Scotland's data governance frameworks, and describes how to access the data through the BHD.

METHODS

Permissions and research governance

The SCANDAN study obtained multicentre research ethics permission from the North of Scotland Research Ethics Committee (23/NS/0017). Permission to access the data for SCANDAN and the pilot phase for the BHD was provided by the NHS Scotland Public Benefit and Privacy Panel for Health and Social Care (PBPP), which scrutinises applications for access to NHS Scotland health data for non-direct care (PBPP applications 2223-0200 and 2223-0005 respectively). [10] During the application process and throughout the study, the SCANDAN team engaged with several Scottish public and patient groups.

Computing environment

The Scottish National Safe Heaven (NSH), commissioned by PHS, where all processing is done, is hosted in Edinburgh Parallel Computing Centre's (EPCC) Trusted Research Environment (TRE), a secure infrastructure which currently hosts twelve Safe Havens. Each Safe Haven is operated under the "Five Safes" framework [11] and the Scottish Government Charter for Safe Havens.[12] Researchers access a secure data sharing and analysis environment with a virtual desktop, under the terms and conditions prescribed by the data controllers. Standard software packages such as R and Python are available in the NSH; additional software packages can be installed from repositories such as the comprehensive R archive network (CRAN) and the Python package index (PyPI). Safe Havens have access to large shared-memory, high-performance computer clusters, including one with graphical processing unit (GPU) accelerators for large-scale analysis. For example, SCANDAN was provided a virtual environment with a GPU (NVIDIA A100 40GB), large storage (several TB) and RAM (100GB). All EPCC Safe Haven Services are operated at EPCC's Advanced Computing Facility, located in Edinburgh, Scotland. The EPCC TRE is accredited by ISO27001[13] for information security practices and self-certified under Cyber Essentials and NHS Digital's Data Security and Protection Toolkit (DSPT). In addition, the NSH is accredited under the Digital Economy Act 2017 by the UK Statistics Authority, and all Safe Havens in the TRE are operated to the same standard.

Data sources

PHS' eDRIS provided brain CT and MRI head studies in adults performed in Scotland between 2010 and 2018 from the Scottish Medical Imaging (SMI) service [14]. Study refers here to a complete imaging session, encompassing all images obtained during a single scanning session.

Each scan contains three hierarchical levels: study, series, and images. Within each study, there are one or more series that group together images acquired using the same imaging technique and settings. Each series is, in turn, composed of multiple single two-dimensional images or "slices”. A patient may have had multiple independent studies. Additionally, imaging reports are associated with studies and contain text about the imaging process and clinical interpretation of images. eDRIS and EPCC linked studies to patients deterministically with pseudonymized identifiers based on the Community Health Index (CHI) number, which is the unique patient identifier used across NHS Scotland. We linked them with outpatient records (SMR00), hospital admission records (SMR01), dementia records from mental health hospitalisations (SMR04), cancer registry (SMR06), community dispensed prescriptions from Prescribing Information System (PIS), death records (National Records of Scotland (NRS) and demographics (birth year, sex, deprivation index) since the year 2000. All the data was processed and stored within the NSH.

Data availability

The data on 830,884 patients was provided to SCANDAN and is available through the BHD. It includes 417,341 MRI studies, 846,077 CT studies and 1.8 million radiological reports. The studies contain 3.37 million MRI series and 3.15 million CT series. Figure 1 shows the distribution of slices per series for both CT and MRI for which the metadata was available at the start of the project. There were 356 million events from EHR, divided between the outpatient emergency and inpatient records (38 million), death records (327,000), prescription records (312 million) and accident and emergency records (4.5 million). For the 410 million DICOM slices available (130.3 million MRI and 279.7 million CT), DICOM metadata were limited by the governance approval, with each tag being subject to approval. Consequently, the accepted metadata were provided separately in CSV format. Table 1 describes key metadata on dataset heterogeneity.

SCANDAN Project

The SCANDAN sequential work packages (WP) are illustrated in Figure 2. The NLP WP identified MRI sequences, CT type and brain pathologies from the radiological reports. The dementia labelling WP phenotyped dementia with EHRs. The cohort building WP selected a subset of MRI studies to carry out dementia classification. The image cataloguing WP labelled DICOM series with body part imaged, sequence, and presence of contrast and then filtered out non-desired scans based on the label. Images were then processed for AI analysis.

SCANDAN: Natural language processing of brain imaging reports

We applied a clinical NLP tool, the Edinburgh Information Extraction for Radiology (EdIE-R), [15,16] which was originally developed and validated for radiology reports of brain imaging in the Edinburgh Stroke Study and NHS Tayside [17]. EdIE-R processes radiology reports through a pipeline that identifies entities, detects negation, extracts relationships and assigns document-level labels to identify phenotypes. The tool was later adapted and validated for use with data from other areas in Scotland provided by Generation Scotland [13]. EdIE-R can extract 24 distinct phenotypes, including different stroke types (ischaemic, haemorrhagic and underspecified, with time and location details), brain tumours (meningiomas, gliomas,

metastases or underspecified), small vessel disease, microbleeds, atrophy and other abnormalities. Additionally, it marked up MRI sequence types (T1, T2, and FLAIR).

To improve data selection, EdIE-R was enhanced to identify scans of non-head and non-brain body parts, and flag them for exclusion. We improved the tool's ability to identify where distinct sections begin and end within reports, such as the boundary between the clinical history preamble and the main report body, enabling us to extract phenotype mentions exclusively from the relevant report text.

EdIE-R contains several processing components. After pre-processing and linguistic analysis (e.g. tokenisation, sentence detection, lemmatisation and part-of-speech tagging) of the text in the input radiology report, EdIE-R performs named entity recognition, negation detection and relation extraction before conducting document-level classification of the 24 phenotypes. The output is the radiology report and its accompanying metadata as well as the information identified by EdIE-R represented in XML format which was then converted to CSV for follow-on analysis. It is keyed by the study identifier and does not contain information about a specific series.

The refined EdIE-R pipeline [16] was applied to all radiology reports in the SCANDAN project, producing structured outputs to guide data selection for image analysis. By processing radiology reports within the Scottish NSH, the tool allows exclusion of scans (e.g., those showing tumours or non-brain regions) and served to validate outputs from imaging type classification and phenotype extraction.

Structured report DICOM contains TextValue elements of various kinds. Some are clinical reports, others contain non-clinical information. We aimed to select the best clinical report in each study for NLP processing. Not all study directories contained structured reports, and some contained more than one. For the latter, the process of choosing a report was as follows. From inspection of examples, it appeared that DICOM files containing real reports normally contained exactly one TextValue element. If a study contained one or more such DICOM files, we used the largest of those. In the cases where no report contained exactly one TextValue element, we just used the largest DICOM file, and processed the first TextValue element. If this was not a clinical report it would usually be marked as “empty” or “nocontent” by the NLP pipeline. Some reports were withheld because they were potentially identifiable, which accounts for studies with no report or no real report.

SCANDAN: Phenotyping dementia

We follow the phenotype specification for dementia [19] based on prior studies in Scottish EHRs [20]. Dementia was defined as the presence of relevant ICD-10 codes, and, for the specific case of Alzheimer's disease (AD), the prescription of AD medications. Each patient interaction with the health system, taken from the EHR, was used including a single stay in hospital, multiple consecutive stays, a prescription, or a death record. We defined labels for ‘any dementia’ and five dementia subtypes: AD, vascular dementia (VaD), other rare dementias, unspecified dementia and possible dementia. The subtype was defined as the most frequently occurring dementia phenotype in each person's electronic record. (Table 2). All individuals with a

dementia label were categorised as cases, and individuals with no mention of dementia in any record were considered controls.

SCANDAN: Cohort building

We built a matched case-control study cohort with MRI brain images. A matched case-control design was chosen for several reasons. First, most deep learning and other algorithms work best with balanced cases and controls. Second, we had limited computing capacity at the beginning of the project. Third, the rate of image delivery was limited by the need to copy data from a preparation area to a research area which had limited storage capacity. We selected images with no NLP label of tumour or haemorrhagic stroke in the radiology report from patients who were aged over 40 years at the time of scan and had an associated EHR record. We excluded dementia cases without scans taken more than one year before the time of diagnosis. For each individual, the first study chronologically was chosen. Dementia cases were matched to controls based on age at the time of scan (within one year of the matched case) and recorded sex from the linked demographic information. Cases with no matched controls were discarded. Age and sex matching was verified by analysing the resulting distributions over the entire cohort. A table was generated containing the identifier of the selected patient, their match, the selected study, demographic information, and the dementia ground truth.

SCANDAN: Data validation and quality control

To generate ground truth labels for an initial evaluation of the automatic labelling process described in the next section, we developed a custom python-based graphical user interface (GUI) optimized for MRI and CT DICOM files. The GUI allowed users to load DICOM images from a single or nested folder structure. It utilized DICOM header metadata to: stack slices according to the acquisition order using the DICOM tag ‘Instance Number‘ (0020,0013), in ascending, descending, or interleave format, constructing and saving the 3D volumes for subsequent analysis; filter CT scans with the tag ‘Modality’ (0008,0060) and adjust their intensities, e.g. brain-windowing, using the tag ‘Rescale Intercept’ (0028,1052); determine the orientation of the imaging planes (i.e., axial, sagittal, or coronal) using the tag ‘Image Orientation (Patient)’ (0020,0037) to display mid-axial, mid-coronal, and mid-sagittal views for assessment; and calculate the aspect ratio using the ‘Slice Thickness’ (0018,0050) and ‘Pixel Spacing’ (0028,0030) tags for accurate scaling and visualising of mid-view slices within the designated display area.

Randomly sampled example series (1,000) were selected prior to the large data delivery without stratification for image review. Among these, 287 were excluded for potential disclosive information. The remaining 390 MRI and 323 CT were annotated by five experts (3 clinicians and 2 trained imaging scientists) with modality, sequence, presence of contrast, lesion and artefacts, presence of full brain, and presence of body parts (Figure 3). The 713 series were evenly divided among the five annotators, with a subset of 100 images overlapping for cross-validation. The Generative model of Labels, Abilities, and Difficulties (GLAD) [14] probabilistic framework was used to estimate the true label for each image while accounting for annotator expertise and image difficulty, by using the overlapping 100 annotations as "truth” and correcting the rest of the annotations.

The ground-truth labels were compared with the labels obtained from the automatic labelling. Scans sequence type and contrast presence were further reviewed by a neuroradiologist and an experienced imaging scientist independently for the disagreement with the automatic labels, or for previous “unknown” or “uncertain” annotations. A third round of annotations resolved disagreements between the neuroradiologist and the imaging scientist. The presence of brain, of other body part and the fullness of the brain was re-annotated by a trained image scientist as an independent re-annotation. After re-annotation, the ground-truth was updated using the most recently agreed version. We report the final comparison of the automatic tools and the manual annotations.

SCANDAN: Identification of MRI scan type and sequence

DICOM tags were used to produce five labels for each image series: imaging sequence, presence of brain, presence of other body part, angiography, and imaging with contrast. We aimed to retain MRI series with sequences T1, T2 and Fluid-Attenuated Inversion Recovery (FLAIR), that contained a brain and no other body part than the neck, without angiography or contrast, and with a 3D image volume of over 5 litres. The volume was empirically determined on another study using Scottish medical data, compared to the manual annotations, and visually asserted to separate two different normal distribution of scans volume.[21] These labels were subsequently combined with the results from the NLP tool, the MRI acquisition parameters, and the computed volume of the image series, to exclude those which did not meet SCANDAN criteria. Figure 4 illustrates the methods and data sources for the labelling and exclusion of images.

To produce these labels, the DICOM tags were parsed with regular expressions (RRID:SCR_028365, biotools:scandan-dicom-labelling).[22] For example, the expressions /(?i)(?<!pa)t2/ (case insensitive and ignoring occurrences starting with “pa”) and /*se2d1/ were associated with the intermediate label "tmp-T2" (T2-weighted), while the expressions /TOF/ and /MRA/ were associated with the intermediate label "tmp-MRA" (MR angiography). Then, the final labels were created by grouping all the intermediate labels of a series. For example, for an image series to be labelled "T1", it had to match the intermediate label "tmp-T1", and could optionally match the intermediate labels "FLAIR", "GRE" (gradient echo), and “FAT SAT” (fat saturation), which are not T1-weighted exclusive, but no any other intermediate label.

For sequence identification, the DICOM tag 'Series Description' (0008,103E) was used. To identify the body part, we used the tags 'Body Part Examined' (0018,0015), 'Protocol Name' (0018,1030), 'Performed Procedure Step Description' (0040,0254) and ‘Study Description' (0008,1030). Angiograms were identified with the tags ‘Angio Flag’ (0018, 00), ‘Study Description' (0008,1030), 'Protocol Name' (0018,1030) and ‘Series Description' (0008,103E). Contrast identification used ‘Study Description' (0008,1030), ‘Contrast/Bolus Agent’ (0018,0010), ‘Contrast/Bolus Route’ (0018,1040), 'Performed Procedure Step Description' (0040,0254) and 'Series Description' (0008,103E).

The results from the NLP tool provided additional information for the sequence identification and the presence of other body parts, validating the presence of a sequence within a study. Finally, to complement the identification based on the series description the MRI sequence was also

defined with the tags ‘Echo Time' (0018,0081), 'Inversion Time' (0018,0082), 'Repetition Time' (0018,0080), 'Scanning Sequence' (0018,0020), 'Flip Angle' (0018,1314), 'Sequence Name' (0018,0024) based on "optimal" value [7-8] adapted to the data through observation on manually annotated data.

Each regular expression rule was based on prior research carried on in-house clinical studies. They were expanded to ignore conflict and formatting due to the greater distribution of value from the 35 hospitals which the data originates from. The most common occurrence for each DICOM tag were compared to their label using the metadata of the entire cohort, to ensure the rules were not including unwanted samples. Finally, they were refined to agree with the manual annotation during each re-annotation.

RESULTS

SCANDAN: Output Dataset

Taking the first chronological study in each sequence of studies (MR or CT) for each person gave 1.1 million studies of which 311,000 were MRI and 789,000 CT. Among these, 16,000 MRI and 119,000 CT were associated with a record of dementia. After applying exclusion criteria, as described in Table 3, 10,709 MRI and 57,242 CT dementia cases were age and sex matched with the same number of healthy controls. We eliminated 1,171 MRI and 3,302 CT dementia cases with a text report containing a mention of tumour or haemorrhagic stroke; 70 MRI and 100 CT dementia cases due to the patient being under 40 at the time of scans; and 4,869 MRI and 58,779 CT cases because the scans occurred within one year prior to the dementia diagnosis.

Of the 1,481,643 study directories, 449,369 had no structured report, 655,450 study directories contained exactly one report and 376,824 study directories contained more than one report. Each selected report was processed using the EdIE-R NLP pipeline. Across the full dataset, the most frequently detected phenotypes were small vessel disease (25.0% of studies) and atrophy (23.2%), reflecting their high prevalence in an ageing clinical population. Ischaemic stroke findings were also common (e.g. old deep ischaemic stroke was detected in 9.4% of studies), while haemorrhagic stroke subtypes were comparatively rare (0.2–2.9%). Tumour-related findings were detected in 0.4–4.1% of studies, depending on subtype. Regarding imaging sequences, T2 was the most frequently recorded (13.4% of studies), followed by T1 (8.3%) and FLAIR (7.8%). These NLP-derived labels formed the basis for the phenotypic exclusion criteria applied to the dementia cohort.

Of the 21,418 MRI studies requested, 21,197 were successfully received, with 221 being excluded for privacy reasons. These studies contained 128,257 series of which 73,457 were identified as T1, T2 or FLAIR, 18,681 series were localisers, 4,372 as unknown, 30,267 were other MRI sequences (DWI, SWI, T2* etc.), and 1,464 series had a series description that did not differentiate T1 and T2*. Table 4 describes the filtering process making use of the DICOM labelling process, which resulted in the exclusion of 2,641 FLAIR, 11,820 T1 and 10,863 T2. After restricting the selection to the first chronological series for each study, 41,966 series were kept from 15,558 studies. Later, 277 studies were excluded when they failed to convert to NIfTI and subsequent process.

The MRI studies requested originated from 35 hospitals across Scotland using 27 unique MRI scanners models were identified (14 models with <100 studies), with 60% of the studies using a Siemens model, 20% General Electric (GE) and 20% Philips, and a handful from 2 other manufacturers. 94% of the scans were done with a 1.5 Tesla, and 6% a 3 Tesla MRI scanner.

The MRI cohort contained 8,145 cases (53.2% female) and 7,236 controls (54.1% female) (Table 5). The mean age at scan was 74 years. The mean time from scan to first mention of dementia was 5 years for cases, and the mean follow-up time for controls was 6 years 9 months. Of the 8,145 dementia cases there was non-exclusive record of AD in 3,774, vascular dementia in 3,386, unspecified dementia in 3,784 and other dementia types in 508. The mean number of hospitalisations in the year prior to scan was 1.1 (standard deviation ([SD] 1.52) for cases and 1.0 (SD 1.50) for controls. During the same period, the mean number of prescriptions was 15.4 for cases and 14.2 for controls.

SCANDAN: Data validation and quality control

For simplicity, we refer to the results of the manual annotations as "annotations", and the results of the automatic tools described in Figure 4 as "labels".

During the first round, 707 annotations were obtained from 713 images. Four images could not be read due to acquisition errors. Two images were only partially annotated due to visual perception errors and discarded. In 29 (4.5%) cases, image modality was wrongly annotated because the series contained only one slice (i.e. a localiser) or did not contain a brain. Of CTs, 24 (7.52%) were classed as 'Unknown'. In the labelling process we used the previously validated DICOM tag 'Modality' (0008,0060) to identify CT and MRI series. The identification of the body parts was easier for the annotators than the modality or sequence type. “Unknown” was given for 23 (3.3%) series when annotators were questioned whether they contained a brain or not, 40 (5.7%) when questioned if the brain was acquired in full, and in 36 (5.1%) series the annotators could not assert whether there was another body part. Annotators could not identify the sequence type for 85 (12.0%) series and the presence of contrast in 104 (14.7%) series.

In this first round of annotations, the main disagreements between annotations and labels were the presence of non-brain images or localisers. For further analyses, the series labelled as 'localiser' in the first round of annotations, defined as series with less than 15 slices, were ignored. If the annotation and label agreed on the absence of brain, the images were not re-annotated.

In the second round of annotations, images with an unknown sequence type (16 series), and those with disagreement between label and annotation ('T1', 'T2' or 'FLAIR', 48 series), were re-annotated. Additionally, a subset of images was selected from 66 with partial agreement between at least one sequence label and the annotation, to validate commonly occurring combination of labels which were not similar (e.g. 'T1' + 'GRE' instead of 'T1' + ’T1’). Series which contained at least one mention of 'T1', 'T2' or 'FLAIR' in either the annotation or the label were re-annotated for presence of contrast when disagreement was found or when they were annotated as 'Unknown'. Series with 'Unknown' annotation for questions regarding brain presence (13 series), other body part presence (8 series) and whole brain (11 series) were

also re-annotated. Disagreement between the annotation and the labels were also re-annotated, respectively 7, 143 and 20 series. In case of a whole brain, the disagreement was ignored if other body parts were present in both the label and the annotation. In total, 143 series were re-annotated for the presence of brain and other body parts, and for full brain coverage. Additionally, there were 84 images re-annotated for sequence type and contrast.

To resolve conflict between the two re-annotators, or between them and the labelling tools, 27 series were then annotated a third time. Some conflicts could not be resolved, such as 7 images having the same 'Series Description' (0008,103E) tag value, and thus the same label. Three of them were identified as T1 and four as T2* by the two annotators in agreement.

Between each round of annotation, the regular expressions used by the labelling tools were updated to reflect previously unknown, and to solve conflicting information and errors.

The results of the labelling tools compared to the final annotation as ground truth were very good. The true positive rate ranged from 87% to 97% and the positive predictive value from 81% to 99% (Table 6). For consistency with our metrics, we evaluated the absence of contrast and other body part respectively as "positive". This value excludes localisers for sequence type, and series without presence of brain for 'whole brains'. The lower precision for detection of other body parts is explained by the lack of mention of any parts in the different DICOM tags, sometimes due to missing data, as well as the detection of some other head parts, such as the jaw, without mention of the brain, which often, but not always, indicate non brain scans. The lower recall for the absence of contrast is caused by the low number of studies that used intravenous (IV) contrast. During a scanning session that used IV contrast, a first image will normally be captured free of contrast, prior to the injection, however, the 'Study Description' (0008,1030) will indicate the presence of IV contrast nonetheless for this first series, as was commonly found.

SCANDAN contribution and BHD data

The SCANDAN project produced data which were added to the BHD. In addition to the 1.2 million brain studies from 830, 000 patients, the 1.8 million radiological report and the 356 million EHR available as raw data, researchers can also access five additional tables: 1. summary of the valid radiological reports generated by the NLP; 2, dementia phenotyping table, with dementia subtype probability and date of diagnosis; 3. patient history, curated and listing all relevant information from all EHR; 4. the manual annotations of the 708 series; 5. the labelling of the 21K MRI of the SCANDAN cohort for MRI sequence, body part imaged, brain fullness and contrast presence. The latter is planned to be expanded to the totality of MRI and then CT scans.

Permissions and governance

The SCANDAN application to the PBPP, which included an industry partner and aimed to develop an AI algorithm, required 210 days spanning 4 iterations for approval from the initial submission and over 17,000 words across 33 pages.

However, with the development of the BHD, researcher can now apply to PBPP to access these data with a shorter application and streamlined process. The data flow and linkage process for

the BHD framework are schematically illustrated in Figure 5. Researchers can log into a workspace running in the NSH with data and tools to perform analysis. To run externally developed tools, they can build a container outside the NSH and pull it from a public registry after approval. It should be noted that no data leaves the NSH during this process. The TRE is divided in several zones (Figure 5). The blue zones, where eDRIS store the data, are not accessible to the researchers. Researchers have access to green zones with access to subsets of the data, as defined by their access permission.

PHS eDRIS will prepare suitable subsets of the data for a particular research group and copy it to their working space. Any results required outside the NSH, e.g., for publication, are subject to disclosure control performed by eDRIS.

DISCUSSION

The BHD is a large-scale, curated brain imaging clinical dataset relevant to dementia research that is available to researchers via moderated public access. The dataset offers several advantages in addition to its large size: clinical relevance, long-term follow-up, co-location with a GPU cluster in a safe haven, greater population representativeness compared to many research cohorts, and accessibility for clinical researchers. The resource continues to grow in data size and computing power.

Working with health systems data presents challenges. One of them is the time taken for governance approval. In our case, if governance had been applied for once funding had been awarded, it would have represented 58% of a 1-year postdoctoral award. This would not only impact negatively on career development of the post holder but also delay the project goals, an issue addressed by the streamlined process of the BHD. Data provision was initially constrained by the limitations of the virtual machine environment, limited staff availability, and increased procedural complexity resulting in delayed access to imaging data and complicating project planning. The experience gained through the SCANDAN project, which piloted the access to the data, allowed PHS to streamline the governance process and improve the data provisioning to future projects. It is important however, to note that all research outputs generated within the NSH must undergo review by PHS staff prior to release.

Most imaging research is based on uniformly acquired research data. In contrast, clinical scans acquired in a routine free-at-the-point-of-service healthcare are sometimes incomplete, may be obscured by movement or other artefacts, show signs of non-relevant pathologies, may have been obtained with non-standardised protocols, and on different machines. However, such real-world data with inherent variability is essential for the development of software tools suitable for robust applications in clinical practice where such heterogeneity is the norm.

Using electronic health records for dementia diagnosis has limitations. Currently, primary care data are unavailable through PHS and thus cannot be provided by the BHD. Hence, we relied on recorded diagnosis after an inpatient stay or death. Hospital and death records under-ascertain (false negatives) dementia in the short term and have modest reliability for dementia subtypes [23]. However, they have also previously shown high positive predictive value for all dementia diagnosis [14]. Referral reasons for scans acquisitions are not currently available, although further NLP work with reports could achieve this.

The use of head scans does raise privacy concerns due to facial recognition risks. We have mitigated these by working only in a safe haven environment, examining only brain slices, prohibiting facial reconstruction, limiting access to approved researchers, who accept the restrictions and conditions of working in the NHS specified in the eDRIS User Agreement which includes PHS strictly checking all outputs from the secure environment, to exclude any identifiable data. Future work aims to further mitigate privacy risks by limiting the need for direct human access to data, for example by implementing software via containers. However, this work needs training of the research community, better labelling of metadata (so the data is truly FAIR), and further development of technology within the NSH environment.

There are many opportunities for further linkage to other datasets (for example community retinal imaging [24]). Such work will require further engagement with public contributors, use of federated analysis and federated learning with ongoing adoption of tools and techniques to assess disclosure risks of different AI models.

The SCANDAN project piloted the access to the data now provided through the BHD. While its primary goal was to establish a proof of concept for dementia classification using clinical data, it produced several secondary outputs which are now available to other researchers using the BHD data. As more projects will use the data, additional output will be added, compounding with time to an unvaluable resource for brain imaging research. Researchers can access the BHD data by applying to PBPP via eDRIS. Proposals must demonstrate a clear public benefit, and researcher- generated outputs must be added back to the dataset so every project strengthens the next. We strongly encourage cross- group collaboration. The resources available through the BHD are growing in terms of data availability, storage capacity, and computing power that are provided to researchers. We hope that this, and similar global initiatives, will ultimately contribute to improve the brain health of people worldwide.

FUNDING

This work was supported by NEURii, a collaborative partnership involving the University of Edinburgh, Gates Ventures, Eisai, LifeArc and Health Data Research UK (HDR UK). We acknowledge the eDRIS team (Public Health Scotland) for their support in obtaining approvals, the provisioning and linking of data and facilitating access to the National Safe Haven. The Brain Health Data Pilot is supported by Alzheimer's Disease Data Initiative (ADDI) and HDR UK with funding to the University of Edinburgh.

CONFLICTS OF INTEREST

MVH and JMW are supported by Row Fogo Charitable Trust (Grant no. BRO-D.FID3668413). JMW was supported by the UK Dementia Research Institute (award no. UKDRI –4002 and 4205, DRIEdi17/18, and MRC MC_PC_17113) which receives its funding from DRI Ltd, funded by the UK Medical Research Council, Alzheimer's Society and Alzheimer's Research UK. ST acknowledges support of the UKRI AI programme, and the Engineering and Physical Sciences Research Council (EPSRC), for CHAI - Causality in Healthcare AI Hub [grant number EP/Y028856/1]. WW and HW are supported by HDRUK.

References

1. Westwood M, Ramaekers B, Grimm S, Armstrong N, Wijnen B, Ahmadu C, et al.. Software with artificial intelligence-derived algorithms for analysing CT brain scans in people with a suspected acute stroke: a systematic review and cost-effectiveness analysis. *Health Technol Assess (Rockv)*. NIHR Journals Library; 2024; doi: 10.3310/RDPA1487.

2. Ferber D, El Nahhas OSM, Wölflein G, Wiest IC, Clusmann J, Leßmann ME, et al.. Development and validation of an autonomous artificial intelligence agent for clinical decision-making in oncology. *Nat Cancer*. Nature Research; 2025; doi: 10.1038/S43018-025-00991-6;SUBJMETA.

3. D'Adderio L, Bates DW. Transforming diagnosis through artificial intelligence. *NPJ Digit Med*. Nature Research; 2025; doi: 10.1038/S41746-025-01460-1.

4. Dugŕ M, Chatelain Y, Glatard T. An analysis of performance bottlenecks in MRI preprocessing. *Gigascience*. Oxford Academic; 2025; doi: 10.1093/GIGASCIENCE/GIAE098.

5. NHS England. Diagnostic Imaging Dataset Annual Statistical Release 2023/24. 2024 Nov.

6. Larobina M. Thirty Years of the DICOM Standard. *Tomography*. Multidisciplinary Digital Publishing Institute (MDPI); 2023; doi: 10.3390/TOMOGRAPHY9050145,.

7. Liang S, Beaton D, Arnott SR, Gee T, Zamyadi M, Bartha R, et al.. Magnetic Resonance Imaging Sequence Identification Using a Metadata Learning Approach. *Front Neuroinform*. Frontiers Media S.A.; 2021; doi: 10.3389/fninf.2021.622951.

8. de Mello JPV, Paixão TM, Berriel R, Reyes M, Badue C, de Souza AF, et al.. Deep learning-based type identification of volumetric MRI sequences. *Proceedings - International Conference on Pattern Recognition*. Institute of Electrical and Electronics Engineers Inc.; 2020; doi: 10.1109/ICPR48806.2021.9413120.

9. : What is eDRIS? - Overview - Electronic Data Research and Innovation Service (eDRIS) - Health intelligence and data management - Resources and tools - Public Health Scotland. https://publichealthscotland.scot/resources-and-tools/health-intelligence-and-data-management/electronic-data-research-and-innovation-service-edris/overview/what-is-edris/ Accessed 2026 Mar 27.

10. : Public Benefit and Privacy Panel for Health and Social Care. https://www.informationgovernance.scot.nhs.uk/pbpphsc/ Accessed 2026 Mar 27.

11. : The Five Safes Framework. https://www.gov.uk/data-ethics-guidance/the-five-safes-framework Accessed 2025 May 28.

12. : Charter for Safe Havens in Scotland: Handling Unconsented Data from National Health Service Patient Records to Support Research and Statistics. https://www.gov.scot/publications/charter-safe-havens-scotland-handling-unconsented-data-national-health-service-patient-records-support-research-statistics/ Accessed 2025 May 28.

13. : ISO/IEC 27001:2022 Information security management systems. https://www.iso.org/standard/27001 Accessed 2025 May 28.

14. Baxter R, Nind T, Sutherland J, McAllister G, Hardy D, Hume A, et al.. The Scottish Medical Imaging Archive: 57.3 Million Radiology Studies Linked to Their Medical Records. *Radiol Artif Intell*. Radiological Society of North America Inc.; 2024; doi: 10.1148/RYAI.220266/ASSET/IMAGES/LARGE/RYAI.220266.FIG2.JPEG.

15. Alex B, Grover C, Tobin R, Sudlow C, Mair G, Whiteley W. Text mining brain imaging reports. *J Biomed Semantics*. England; 2019; doi: 10.1186/s13326-019-0211-7.

16. : Software – Language Technology Group. https://www.ltg.ed.ac.uk/software/ Accessed 2025 Aug 14.

17. Wheater E, Mair G, Sudlow C, Alex B, Grover C, Whiteley W. A validated natural language processing algorithm for brain imaging phenotypes from radiology reports in UK electronic health records. *BMC Med Inform Decis Mak*. 2019; doi: 10.1186/s12911-019-0908-7.

18. Casey A, Davidson E, Grover C, Tobin R, Grivas A, Zhang H, et al.. Understanding the performance and reliability of NLP tools: a comparison of four NLP tools predicting stroke phenotypes in radiology reports. *Front Digit Health*. Switzerland; 2023; doi: 10.3389/fdgth.2023.1184919.

19. : Phenotype Library | Phenotype: Dementia Identification with EMR. https://phenotypes.healthdatagateway.org/phenotypes/PH1717/version/3973/detail/ Accessed 2026 Mar 21.

20. Doney ASF, Bonney W, Jefferson E, Walesby KE, Bittern R, Trucco E, et al.. Investigating the Relationship Between Type 2 Diabetes and Dementia Using Electronic Medical Records in the GoDARTS Bioresource. *Diabetes Care*. American Diabetes Association; 2019; doi: 10.2337/dc19-0380.

21. Reel PS, Al-Wasity S, Edwards C, Reel S, Mansouri-Benssassi E, Suveges S, et al.. Machine learning-based prediction of future dementia using routine clinical MRI brain scans and healthcare data. *medRxiv*. Cold Spring Harbor Laboratory Press; 2025; doi: 10.1101/2025.11.12.25340070.

22. : GitHub - SCANDAN-Team/SCANDAN-DICOM-labelling: Rules for DICOM tag based labelling · GitHub. https://github.com/SCANDAN-Team/SCANDAN-DICOM-labelling Accessed 2026 Mar 30.

23. McGuinness LA, Warren-Gash C, Moorhouse LR, Thomas SL. The validity of dementia diagnoses in routinely collected electronic health records in the United Kingdom: A systematic review. *Pharmacoepidemiol Drug Saf*. John Wiley and Sons Ltd; 2019; doi: 10.1002/PDS.4669.

24. Tochel C, Bernabeu MO, McTrusty A, Tatham AJ, Pead E, Buckmaster F, et al.. SCONe: a community-acquired retinal image repository enabling ocular, cardiovascular and neurodegenerative disease prediction. *BMJ Health Care Inform*. BMJ Publishing Group; 2025; doi: 10.1136/BMJHCI-2024-101236.

TABLES

Table 1: Number of unique values and percentage of empty fields for key brain scans DICOM tags. Tags like 'Institution Name' or 'Body Part Examined' contains different values with the same meaning (e.g. "Head&Neck", "head and neck", "HEAD_NECK"). These were counted as different occurrence.

	Number		% series with empty field	
DICOM tag	MRI	CT	MRI	CT
'Protocol Name' (0018,1030)	185,591	4,029	1.59%	12.27%
'Series Description' (0008,103E)	56,624	17,826	1.73%	< 1%
'Performed Procedure Step Description' (0040,0254)	3,962	3,125	3.69%	79.13%
'Body Part Examined' (0018,0015)	1,821	243	53.88%	41.13%
'Institution Name' (0008,0080)	722	611	1.32%	< 1%

Table 2: International classification of diseases 10 (ICD-10) and British national formulary (BNF) codes to define dementia subtypes. The definition of the codes follows the phenotyping employed previously.[19] * indicates a wild-card, meaning that all child-codes in the hierarchy are included.

Subtype	ICD10	BNF
Alzheimer's disease	F00* G30*	0411000
Vascular dementia	F01*	
Other rare dementias	F02*, G31.0, A81.0	
Unspecified dementia	F03, F05.1	
Possible dementia	F05.0, G31	

Table 3: Selection process for the MRI and CT cohorts, showing both the overall and dementia counts, with addition of characteristics

Criterion	MRI		CT	
	Overall	Dementia	Overall	Dementia
**With scan**	294,422	16,819	669,539	119,423
**+ No reported tumour or haemorrhagic stroke**	280,549	15,648	640,400	116,121
**+ Hospitalised electronic health record**	279,004	15,648	638,680	116,121
**+ Diagnosis other than ‘possible’ dementia**	275,970	15,648	627,911	116,121
**+ Age at scan > 40**	207,876	15,578	523,855	116,021
**+ Dementia diagnosis or follow-up > 1 year**	190,582	10,709	391,356	57,242

MRI: magnetic resonance imaging of brain; CT: computerised tomography of brain

Table 4: Selection process based on the automatic labelling for the MRI cohort. The “Baseline” column shows the number of series for each sequence type prior to exclusion. The "Selected" column show the number of series kept after the exclusion process ready for analysis. Every column in between is associated with an excluding step and shows the number of series iexcluded. Non brain scans, scans with only partial brain, brain scans including other body parts, scans with contrast or angio scans were excluded iteratively. Finally, only one series of each sequence was kept per study.

	Reason for exclusion						
Sequence	Baseline	Brain Absent	PartialBrain	Other Body Parts Present	Contrast or Angio Present	More Than One Series per Study	Selected
FLAIR	16,871	-60	-211	-1,742	-628	-714	13,516
T1	27,627	-1,290	-4,670	-2,707	-3,153	-2,156	13,651
T2	28,959	-1,407	-4,828	-3,738	-890	-3,297	14,799

Table 5: Distribution of subjects with mention of each dementia type and, controls grouped by key characteristics

		Alzheimer's	Vascular	Other or rare	Unspecified	Controls
**Sex**	Female	2,057	1,681	207	2,047	3,917
	Male	1,717	1,705	301	1,737	3,319
**Age in years**	Mean (SD)	73 (8.8)	75 (8.7)	67 (9.3)	74 (8.9)	74 (9.0)
	40-50	36	32	19	38	87
	51-60	282	169	90	227	434
	61-70	968	706	179	813	1611
	71-80	1,694	1,503	182	1,664	3,182
	81+	794	976	38	1,042	1,922
**SIMD**	Mean (SD)	2.8 (1.3)	2.7 (1.3)	2.8 (1.3)	2.7 (1.3)	2.8 (1.3)
	1	719	768	102	842	1429
	2	732	650	102	724	1380
	3	753	635	94	703	1381
	4	909	691	113	780	1523
	5	363	242	42	325	804
**Hospitalisation 1 yr before scan, Mean (SD)**		0.9 (1.4)	1.2 (1.5)	1.0 (1.6)	1.1 (1.5)	1.0 (1.5)
**Prescriptions 1 yr before scan, Mean (SD)**		14.5 (9.5)	16.1 (0.2)	14.3 (9.7)	15.6 (10.2)	14.2 (9.4)

SD: standard deviation; SIMD: Scottish index of multiple deprivation

Table 6: The metrics of the comparison between the automated labels and the manual annotation as ground truth for image characteristics.

Algorithm identified image characteristics	Recall (%)	Precision (%)
sequence type	94.1%	98.6%
study contains brain	97.1%	91.0%
absence of other body parts	93.1%	81.9%
whole brain in study	96.4%	95.6%
absence of contrast in study	87.3%	95.4%

FIGURES

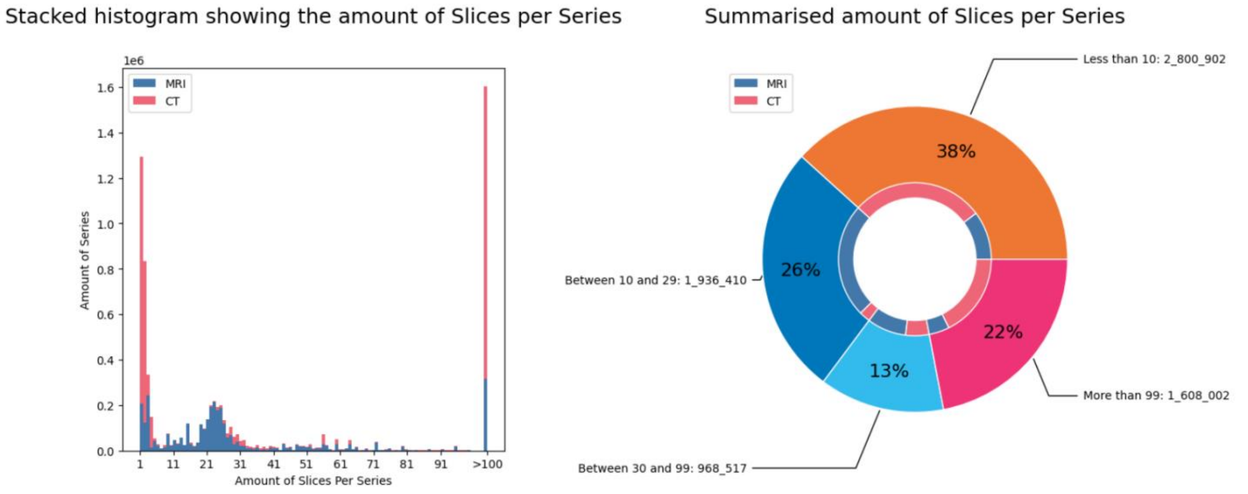

Figure 1: The distribution of slices per series within the data available through the BHD, for both MRI and CT. The stacked histogram shows the 2D MRI have a normal distribution of scans centered around 22, while the 3D MRI have over 100 slices. The CT have mostly more than slices. Both modalities have a large amount of localiser scans with under 10 slices.

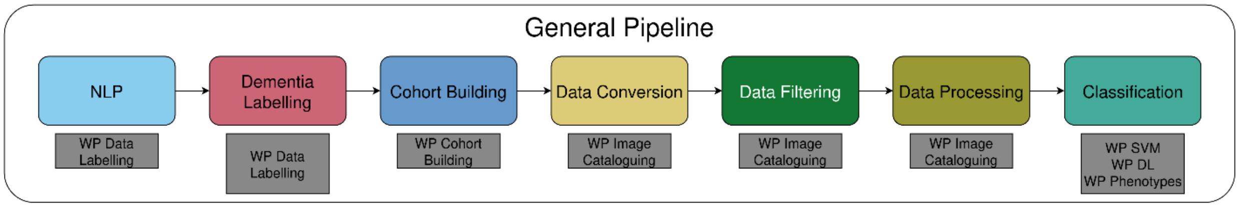

Figure 2: Work packages (WP) in the SCANDAN project: Data labelling, cohort building, image cataloguing, and processing for classification into being indicative of having dementia or not using deep learning (DL), support vector machine (SVM) and from the analysis of extracted imaging phenotypes.

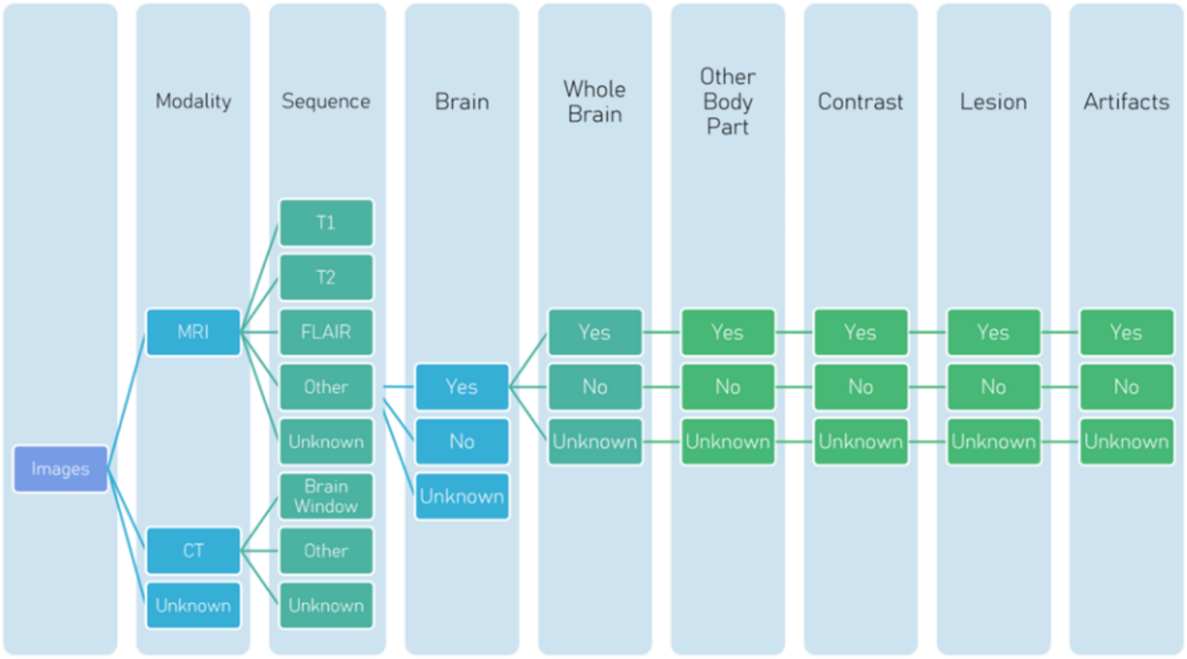

Figure 3: Criteria used by the annotators to label the test imaging set using the GUI developed.

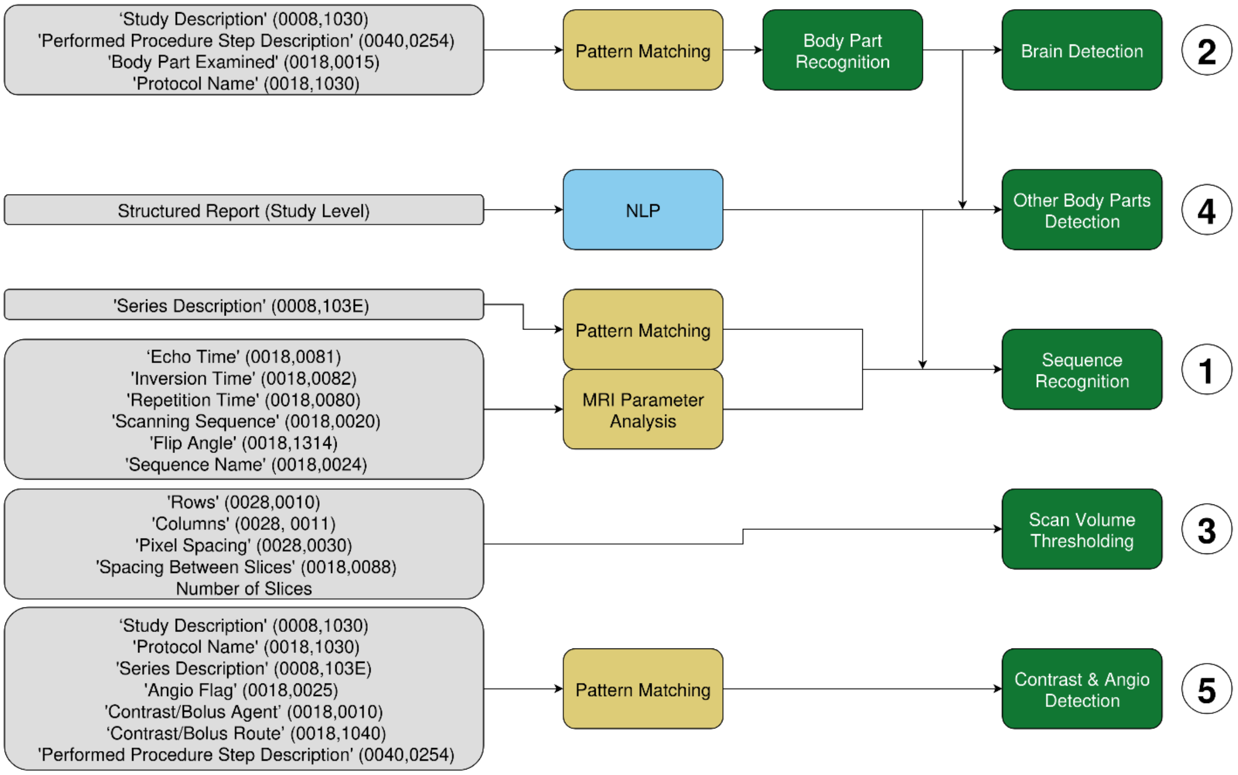

Figure 4: The exclusion process for the SCANDAN cohort. It is based on the automatic labelling (in yellow, including the pattern matching and the MRI parameter analysis) and the NLP of the radiological report (in blue). The steps were performed in the order from 1 to 5, numbered on the right. 1) Non T1/T2/FLAIR scans are excluded. 2) Scans without a brain are excluded. 3) Scans too small to contain a full brain are excluded. 4) Scans with other body parts (such as the spine) are excluded. 5) Contrast and angio scans are excluded.

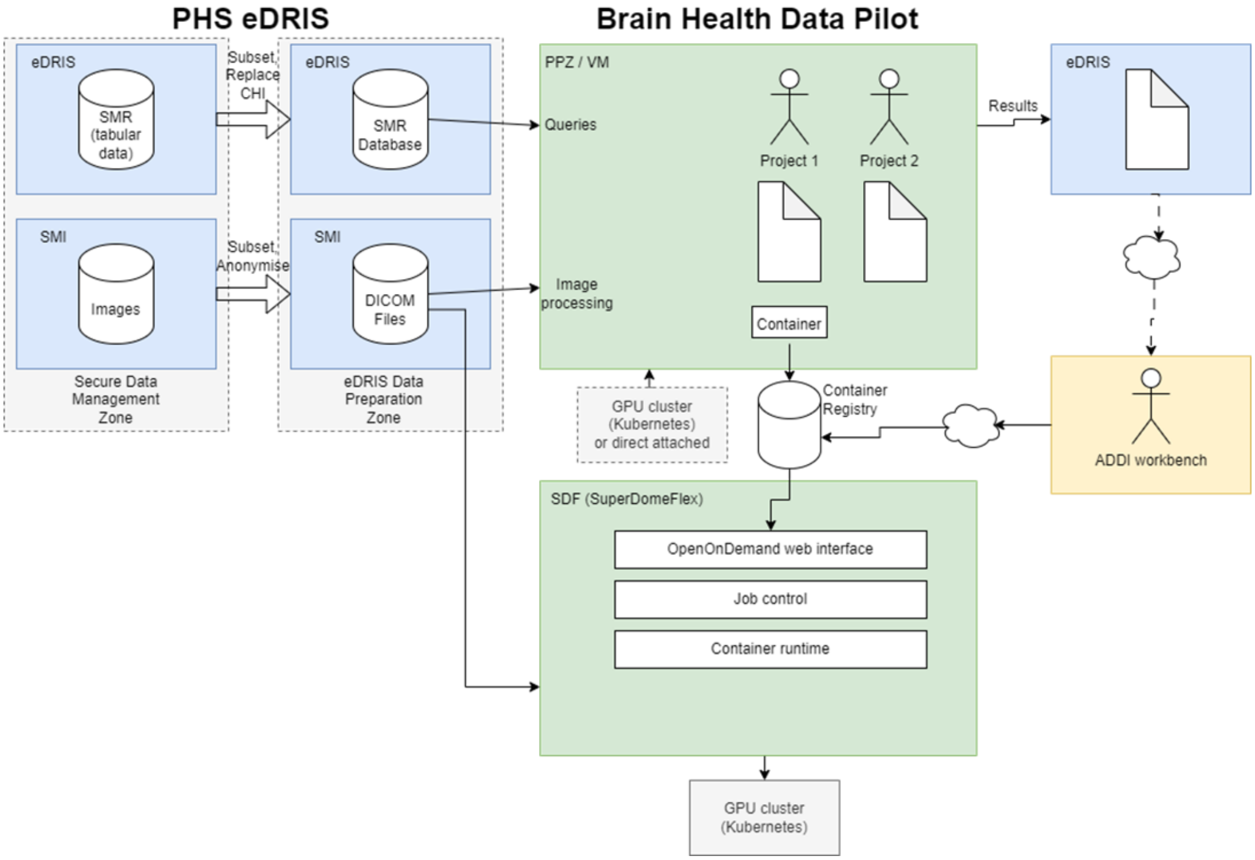

Figure 5: Diagram to illustrate the data flow and data linkage process in the Brain Health Data service (BHD). eDRIS Electronic Data Research and Innovation Service (eDRIS), SMR: Scottish Morbidity Record, CHI Community Health Index number, PPZ: privacy preserving zone, VM: virtual machine, DICOM: Digital Imaging and Communications in Medicine

Manuscript with markup

Click here to access/download
Supplementary Material
Methodology paper v11_with markup.docx

Response to reviewers

Click here to access/download
Supplementary Material
Rebuttal_v2_2.docx
