# Supplementary material for: A large dataset of brain imaging linked to health systems data: curation and access to a whole system national cohort from NHS Scotland
Source: Gigascience. 2026 Jun 9;15:giag072. doi: 10.1093/gigascience/giag072 (PMC13347094; doi:10.1093/gigascience/giag072)
Supplement: giag072_Authors_Response_To_Reviewer_Comments_original_submission [file giag072_authors_response_to_reviewer_comments_original_submission.pdf]

Author's Response To Reviewer Comments

A large dataset of brain imaging linked to health systems data: the curation and access to a whole system national cohort from NHS Scotland

Response to Reviewers

We thank the reviewers for the time they spent and their helpful comments. Both of them pointed out inconsistencies in the scope and objectives of the paper, and their insight was very valuable in reframing the narrative of this note to align with our original goals. We also thank the editor for their time they allocated to our paper.

Reviewer 1:
1. The abstract and introduction emphasize the full raw scale of the dataset (hundreds of thousands of MR/CT scans) and the curation framework, whereas the Results section focuses primarily on a derived dementia-matched cohort and a subset of retrieved studies. Please clearly separate these aspects and report, with explicit counts, the total number of raw imaging studies available in SMI (MRI and CT), as well as the number of unique patients.
answer: Agree - revised
- ABSTRACT: We clarified the abstract by adding the size (45K scans) of the SCANDAN cohort and the type of images used (MRI). We have made clear the data is from a subset of the BHD data (Abstract, par. 1).
- INTRODUCTION: We clarified the data available in the BHD (MRI, CT, electronic health record (EHR) and free text radiology report). We also clarified the distinction between the BHD data and the SCANDAN pipeline, introducing clearly the aims of both and their relationship (Introduction, par. 5).
- METHODS: We added a new subsection called "Data Availability" which lists the number of scans, report and EHR available through the BHD, as well as the number of patients. We included the count for different subset of data sources, such as the different EHR.
- FIGURE: We added Figure 5, which shows the distribution of slices per series, emphasising the scale of the dataset. (Methods, Data Availability)
- RESULTS: We changed the previous subsection "Dataset description" to "SCANDAN Output Dataset", to now reflect properly the distinction between the BHD data (the raw dataset) and the results of the curation framework.
- NARRATIVE: The addition of those two subsections, as well as the reorganisation of the introduction was done to resolve the first point of the reviewer here, highlighting the difference in focus within the paper between the introduction and the results. To this end, we also renamed the Methods subsection relevant to SCANDAN by prefixing them with SCANDAN. Overall, our change aim to make sure the aim of the paper is clear; the introduction of the BHD, the presentation first pilot project carried with its data, and the additional data which it provided to the BHD

2. Please provide more detailed information on the subset of scans that successfully passed the pipeline filters (e.g., T1/T2/FLAIR sequences, whole-brain coverage, absence of contrast or angiography). Although Table 5 partially addresses this, its framing could be clearer—for example, please clarify whether the "Final" category corresponds to the number of research-ready MRI series per sequence.
answer: Agree – revised
- TABLE: We renamed the last column of table 5 from "final" to selected. We also expanded the caption to clarify the meaning of each column.
- RESULTS: We referenced Table 5 in the results, providing additional precision on the number of scans excluded by the label and the number of scans selected as the "first chronologically within the study" and listing the total amount of studies. (Results, SCANDAN Output Dataset, par. 3)

3. Please clarify what data are currently accessible to external researchers through eDRIS/BHD. For instance, does access include raw DICOM files, NIfTI conversions, derived labels, structured report phenotypes, or predefined example cohorts?
answer: Agree – revised
- METHODS: The "Data Availability" subsection lists all the data contained within the BHD

- RESULTS: A new section named "SCANDAN Contribution and BHD data" was added, explaining which data are available within the BHD (referencing the one listed in the "Data Availability" subsection), as well as listing the 5-output produced by SCANDAN. It describes each of these outputs, including their scope (whether it refers to the whole dataset, or the SCANDAN cohort). Each of these outputs are described in more details in the other Results subsection "SCANDAN Output Dataset".

4. The manuscript describes rule-based parsing of multiple DICOM tags using regular expressions, together with MRI parameter checks (e.g., TR/TE/TI), to define sequence labels. Please provide a complete list of the regular expression patterns used for sequence, body-part, angiography, and contrast identification, along with information on versioning and how updates were handled across iterative annotation rounds.
answer: Partially Agree - revised
- A paragraph was added to clarify the creation of the regular expressions (based on prior in-house study, expanded to include variation from all the hospitals, then using the most common occurrence of each tag to make sure nothing is widely excluded, before finally using the manual annotations to refine them). (Methods, SCANDAN: Identification of MRI scan type and sequence, par. 5)
- We provided a link to a GitHub repository containing the regular expression (Methods, SCANDAN: Identification of MRI scan type and sequence, par. 2)

5. Please clarify how the criterion "3D image volume > 5 litres" is computed and provide a rationale for the choice of this threshold.
answer: Agree – revised
- We clarified how the threshold was chosen based on empirical test, from a previous study using similar data. We compared the volume to the annotations to assert it was correct. We added a citation to it (Singh Reel et al). (Methods, SCANDAN: Identification of MRI scan type and sequence, par. 1).

6. The expert annotation study (n = 713 scans across MRI and CT, with 100 overlapping cases and GLAD adjudication) is a strong component of the work. Please clarify how these 713 scans were sampled (e.g., randomly across sites, scanners, and years; stratified by modality; or enriched for challenging or ambiguous cases).
answer: Agree - revised
- We clarified that the data was randomly sampled by EPCC to be provided as example data (Methods, SCANDAN: Data validation and quality control, par. 2)

7. The manuscript currently lacks a concise summary of key imaging metadata. For neuroimaging researchers, it would be highly valuable to include aggregate summaries of:
(1) scanner vendors/models and magnetic field strengths (for MRI), and their distribution across sites and over time;
(2) typical voxel sizes, slice thickness distributions, and acquisition planes for the retained sequences; and
(3) rates of partial brain coverage or localizer scans.
answer: Partially Agree - revised
- We added the number of localiser (along other labels) (Results, SCANDAN Output Dataset, par 3.)
- We added the amount of hospitals site, MRI devices and vendors contained within the data delivered to SCANDAN (21K studies MRI). (Results, SCANDAN Output Dataset, par 4.)
- As a proxy to slice thickness/voxel sizes, we added the distribution of slices per series within Figure 5. This data is obtained on the whole data from the BHD, including CT
- The information asked could not be provided on the entire scale of the BHD for some case due to the metadata not being available. (Methods, Data Availability)

8. Please indicate whether defacing is performed or feasible within the Trusted Research Environment for MR T1-weighted volumes, and whether defacing is mandatory for any form of data export (recognizing that raw images may not be exported, but derived images could be generated).
answer: Partially Agree – partial revision
- No defacing was performed.
- No unconsented images can be exported from the secure data environment.
- The discussion already provides a paragraph on privacy concern (Discussion, par 5.). It also includes two mentions that any data taken out of the Trusted Research Environment need to be validated by PHS

(Discussion, par. 2 & par. 5).
- We added to the discussion the mention that PHS review the data to "excludes any identifiable data." (Discussion, par 5).

9. The manuscript alternates between the terms "scan," "study," "series," and "image." Although these are defined earlier, some later statements could be clarified to avoid ambiguity. For example, the phrase "each study (i.e., one per person)" could be interpreted as implying that each individual has only one study, whereas later sections refer to selecting the "earliest studies associated with these subjects." Please clarify whether "one per person" reflects a deliberate selection criterion or an inherent property of the source data.
answer: Agree - revised
- We clarified how the linkage was done by eDRIS and EPCC (not us) and how it linked the study (scans) to "one person" (i.e. a patient) from the EHR (Methods, Data Sources, par. 1)

Reviewer 2:
1. Several figures aim to provide an overview of the data curation and processing pipeline, but the relationship between the overall SCANDAN project, the specific scope of this Data Note, and the individual curation steps described in the Methods is not always clear. In particular, Figure 1 illustrates the work packages of the overall SCANDAN project; however, it is not immediately clear how these relate to the specific focus of this manuscript. While SCANDAN aims to develop and evaluate dementia classifiers, this paper appears to focus primarily on the data curation and preparation framework. I encourage the authors to clarify in the text and figure legend which components of Figure 1 are directly covered in this manuscript, and which are shown for broader project context.
answer: Agree – revised
- ABSTRACT: we clarified the data curation pipeline was linked to the SCANDAN project (Abstract, par. 1), and was processing a subset of the BHD MRI data, adding the details on the relationship between the two projects (Abstract par. 1).
- INTRODUCTION: We reformulated the definition of the BHD, by focusing on the data it provides. We then explained SCANDAN, a project aiming to perform dementia classification, was the first to use the BHD data. We further explained that SCANDAN produced several outputs when processing the data which were added to the BHD, such as parsing of the radiological text report with NLP, or the dementia phenotyping. Finally, we summarised the relevant part of SCANDAN which are discussed in this paper, aiming to create a research ready dataset, demonstrating what is available within the BHD (Introduction par. 5).
- METHODS: We added a new subsection "SCANDAN Project", referencing Figure 1, which replace the previous explanation from the introduction, and explains in detail how each part interact together, clarifying the SCANDAN project (Methods, SCANDAN project)
- RESULTS: We added a new subsection "SCANDAN Contribution and BHD data" showing the relationship between SCANDAN output and the BHD. The SCANDAN output will be available through the BHD for other researcher, significantly speeding up their research by saving them the time to carry the same, complex, data curation and processing (Results, SCANDAN Contribution and BHD data).
- NARRATIVE: The addition of these 2 subsections, as well as the reorganisation of the introduction was done to address the reviewer point on the confusing relationship between SCANDAN and the BHD, highlighting the goal and scope of the paper were not clear. The BHD provides access to data and easier governance, and the SCANDAN project uses this data, demonstrating its usefulness, providing important demographic information on the dataset, and provided several key outputs which were added to the BHD. We hope those changes clarify the interlinked relationship the BHD and SCANDAN have.

2. Multiple data selection and refinement steps are described across the manuscript, including DICOM-based filtering, NLP-driven exclusions, manual annotation, and iterative re-annotation, but their sequencing and respective roles can be difficult to follow. Figure 4 appears to attempt to bring these elements together, yet it is not explicitly referenced in the text, and its legend lacks sufficient explanation to map the visual elements (including the numbered markers) to the corresponding methodological steps. More explicit cross-referencing between text and figures, and a concise summary (e.g. a short paragraph, table, or expanded figure legend) outlining the full sequence of automated and manual steps, would help clarify how each contributes to the final curated dataset.
answer: Agree – revised
- FIGURE: Figure 4 was referenced within the text (Methods, SCANDAN: Identification of MRI scan type and

sequence, par. 1). Its caption was extensively rewritten to explain what each colour means, what each block does, and in which order they were performed. (Figure 4, Methods, SCANDAN: Identification of MRI scan type and sequence).
- METHODS: the new subsection "SCANDAN Project" also clarified the order in which the SCANDAN work package were used and how they interacted together, making it clearer what was done and when (Methods, SCANDAN Project).
- RESULTS: The subsection "SCANDAN Output Dataset" replace the previous "Dataset description" and provides a detailed description of the exclusion steps, with input and output numbers, referencing the summary Tables (3, 4, 5) which list the information more concisely (Results, SCANDAN Output Dataset)
- NARRATIVE: We reorganised the Methods, moving "SCANDAN: Identification of MRI scan type and sequence" after "SCANDAN: Data validation and quality control". We also clarified which parts were linked to SCANDAN by prefixing the subsection title with "SCANDAN".

3. Data sources section: the description of data linkage and processing (e.g. "We linked each study…", "All data was processed and stored…") would benefit from clarification on which steps are automated and which require manual intervention.
answer: Disagree – minor revision for clarification
- We clarified how the linkage was done by eDRIS and EPCC (not us) and how it linked the study (scans) to "one person" (i.e. a patient) from the EHR (Methods, Data Sources, par. 1)
- The storage, just like the linkage, is performed by ePCC and is inherent to the National Safe Haven. If the reviewer is referring to the rest of the processing described in the Methods, we believe each relevant section highlight the steps were done automatically, save for the annotation.

4. NLP tool: it would be useful to clarify whether the NLP tool is openly available (e.g. as code or a container), as this could be of significant value to the community. In addition, the sentence on page 6 describing the "enhancement of the detection of section boundaries" is highly technical and difficult to follow in this context; a brief rephrasing or explanation of what is meant by section boundaries would help. More generally, a short high-level description of the NLP inputs, outputs, and how these outputs are used within the broader framework would improve readability.
answer: Agree – revised
- We added a citation with a link to EdIE-R (Methods, SCANDAN: Natural language processing of brain imaging reports, par. 4).
- We re-phrased the sentence about section boundaries to simplify it, explaining we refer to the boundary between the report and the clinical history (Methods, SCANDAN: Natural language processing of brain imaging reports, par. 3).
- We provided an additional paragraph summarising the NLP methodology to select the radiological report used as input and their filtering (Methods, SCANDAN: Natural language processing of brain imaging reports, par. 5).
- We also provided a summary of the NLP report output in the Results, listing some of the finding, as well as the numbers of report processed (Results, SCANDAN Output Dataset, par. 2)

5. Phenotyping dementia: this section appears to be missing a reference to Table 1. In addition, Table 1 would benefit from a more informative legend explaining how it should be interpreted. For example, it is unclear why a BNF code is reported only for Alzheimer's disease. While the use of wildcards is understandable, the rationale and intended use of these wildcards should be explicitly stated in the table legend.
answer: Agree – revised
- We added two references to support out method for phenotyping dementia (Doney et al (2019), Doney et al (2025)). We also clarified why the BNF code is only used for Alzheimer's as it's the only that have specific medication. (Methods, SCANDAN: Phenotyping Dementia)
- We added the reference to Doney et al (2025) to the caption of Table 1, and clarified the meaning of the wildcards as well as the reason for their usages, i.e., to that all child-codes in the hierarchy are included (Table 1, Methods, SCANDAN: Phenotyping Dementia)
- The reference to Table 1 was fixed.

6. Cohort building: the description of cohort selection (e.g. "We first selected… gone through the pipeline… and had no NLP label") would benefit from clearer specification of the order of steps, and of which steps

are automated versus manual. Explicit input/output descriptions and a reference to a visual flowchart would help clarify this process.
answer: Partially agree – revised
- The order of the steps for the cohort building was clarified, by removing some steps happening later, which were introducing confusion by being mentioned this early. Specifically, we removed the mention to the image filtering based on the DICOM tag. We kept the exclusion using NLP mention of tumour or strokes. (Methods, SCANDAN: Cohort Building, par. 2)
- The cohort building input, output and the number of excluded samples, referenced in Table 3, were added to the results (Results, SCANDAN: Data).
- We did not add another flowchart. However, we expect the rest of the modification on the paper, about signposting, cross-referencing, and clarification of the order of the SCANDAN project, will clarify the order in which each step was done. None of these steps were "manual".

7. DICOM tags: similarly, the description of how DICOM-derived labels are combined with NLP outputs would benefit from clearer sequencing and explicit reference to a flowchart illustrating the full pipeline.
answer: Agree - revised
- A reference to Figure 4 has been added (Methods, SCANDAN: Identification of MRI scan type and sequence, par. 1). The caption of Figure 4 has been improved to explain the sources of the data, illustrating exactly which tags are used for which task, through which methods. It also shows the combination of the NLP and the pattern matching methods using DICOM (Figure 4, Methods, SCANDAN: Identification of MRI scan type and sequence).
- We added a sentence about how the NLP is used to validate the presence of a sequence within a study (Methods, SCANDAN: Identification of MRI scan type and sequence, par. 4).

8. Data validation and quality control: Table 2 appears to relate to this section but is only explicitly referenced in the Results section. It is also unclear what components are included in the "automated labelling process described above" (e.g. DICOM-based rules only, NLP-derived information, or both). Clarification would also be helpful on what constitutes the "ground truth" used for this comparison, in particular whether the results in Table 2 reflect the final labels after all rounds of annotation, GLAD aggregation, and expert adjudication.
answer: Disagree – partially revised anyway
- Table 2 is only explicitly referenced in the results section as it shows the results of the Data validation and quality control. We clarified in the methods that we are reporting in the results the final comparison of the labelling and the annotation (Methods, SCANDAN: Data validation and quality control, par. 3).
- The automatic label refers the output of the tools described in the section "SCANDAN: Identification of MRI scan type and sequence" (now "next section" previously "above"). There are one group of labels described in this section, described in paragraph 1, with the process to obtain them described in paragraph 1 to 3. Those labels described match the one which were annotated. We hope the clarification concerning NLP usage and the expanded caption for Figure 4, now properly referenced in the relevant section, will explain that the label compared are the output of the agglomeration of those.
- We clarified the caption of Table 2 to reflect that the manual annotation is used as ground truth to assess the labelled obtained automatically (Table 2, Results, SCANDAN Output Dataset)
- We also added clarification regarding the comparison between the automatic tools and the manual annotations using the final versions (Methods, SCANDAN: Data validation and quality control, par 3.; Results, SCANDAN: Data validation and quality control, par 7.)

9. Use of GLAD: the role of GLAD in the workflow would benefit from further clarification. Specifically, it would be helpful to briefly state what inputs are provided to GLAD, what outputs are produced, and how these outputs are used in the evaluation of the automated labelling. It is currently unclear whether the results in Table 2 are based on GLAD-inferred labels or on subsequent manually adjudicated labels.
answer: Agree – revised
- We updated Table 2 caption to reflect that the ground truth is the manual annotation (Table 2, Results, SCANDAN: Data validation and quality control)
- We updated the explanation of GLAD usage as it had been previously erroneous. GLAD was not used to infer automatic label but used on the automatic label. It used the overlapping 100 annotations as "ground truth" and corrected the rest of the annotation (Methods, SCANDAN: Data validation and quality control, par. 2 & 3).

- We hope the clarification of how GLAD was used will answer the reviewer comment and may clarify some of their other comments. We thank the reviewer for pointing out the confusion.

10. Results of data validation and quality control: again, clarification on what is included under "automated tools" would help ensure consistency with the workflow described in the Methods. The authors might also consider illustrating the iterative annotation and adjudication process in a dedicated flowchart.
answer: Disagree – partially revised anyway
- We added a mention of Figure 4 in the results section to ensure the automatic tools and labels are referenced (Results, SCANDAN: Data validation and quality control).
- We added a sentence mentioning how we report the final comparison between the automatic tools and the label (Methods, SCANDAN: Data validation and quality control, par. 3)

11. Discussion: the authors note that the dataset will continue to expand in size; a brief summary of future plans for cohort growth and linkage would be of interest.
answer: Agree - revised
- We expanded on the plan to add NIfti and other processed images available to other project, in a similar fashion to what SCANDAN provided with the metadata and table output (Discussion, par. 7)
- We also clarified that one of the output of SCANDAN, the sequence/body/contrast/... labelling, was carried only on the 21K study from the SCANDAN sub cohort, but are planned to be expanded to the rest of the data in the BHD (Results, SCANDAN Contribution and BHD data)

12. Phenotyping dementia - a reference appears to be missing.
answer: Agree - revised
- Reference to table 1 fixed

13. Figure 3 legend: consider expanding it to include the explanation of colours and acronyms currently provided only in the text.
answer: Agree - revised
- Figure 3 legend now updated to: Figure 3. Diagram to illustrate the data flow and data linkage process in the Brain Health Data service (BHD). eDRIS Electronic Data Research and Innovation Service (eDRIS), SMR: Scottish Morbidity Record, CHI Community Health Index number, PPZ: privacy preserving zone, VM: virtual machine, DICOM: Digital Imaging and Communications in Medicine

14. Page 11: "sizer" appears to be a typo and should read "size".
answer: Agree - revised

em Editorial Manager®

Cookie Preference Center

We use cookies which are necessary to make our site work. We may also use additional cookies to analyze, improve and personalize our content and your digital experience. For more information, see our Cookie Policy.

You may choose not to allow some types of cookies. However, blocking some types may impact your experience of our site and the services we are able to offer. See the different category headings below to find out more or change your settings.

Allow all

Manage Consent Preferences

Strictly Necessary Cookies

Always active

These cookies are necessary for the website to function and cannot be switched off in our systems. They are usually only set in response to actions made by you which amount to a request for services, such as setting your privacy preferences, logging in or filling in forms. You can set your browser to block or alert you about these cookies, but some parts of the site will not then work.

Functional Cookies

These cookies enable the website to provide enhanced functionality and personalisation. They may be set by us or by third party providers whose services we have added to our pages. If you do not allow these cookies then some or all of these services may not function properly.

Performance Cookies

These cookies allow us to count visits and traffic sources so we can measure and improve the performance of our site. They help us to know which pages are the most and least popular and see how visitors move around the site.

Targeting Cookies

These cookies may be set through our site by our advertising partners. They may be used by those companies to build a profile of your interests and show you relevant adverts on other sites. If you do not allow these cookies, you will experience less targeted advertising.

Cookie List

Clear

Apply Cancel

Consent Leg.Interest

Confirm my choices

Power
