# Supplementary material for: A large dataset of brain imaging linked to health systems data: curation and access to a whole system national cohort from NHS Scotland
Source: Gigascience. 2026 Jun 9;15:giag072. doi: 10.1093/gigascience/giag072 (PMC13347094; doi:10.1093/gigascience/giag072)
Supplement: giag072_Authors_Response_To_Reviewer_Comments_revision_1 [file giag072_authors_response_to_reviewer_comments_revision_1.pdf]

Author's Response To Reviewer Comments

Response to Reviewers
We are grateful to the editor and the reviewers for carefully reviewing out manuscript and making suggestions for improvement.

Reviewer 1:

1. The description of dataset scale still remains largely inconsistent and potentially confusing. The manuscript reports quantities at different hierarchical levels (e.g., "417,000 MRI", "3.37 million series", "409 million slices"), but does not consistently clarify whether counts refer to studies, series, slices, or unique individuals. For example, it is not always clear whether "417,000 MRI" refers to studies or patients, which represents fundamentally different dataset scales. A unified and explicit reporting of counts at each level (patients, studies, series, slices) is still needed to make the dataset interpretable and comparable.

answer: Minor revision

We have endeavoured to clarify these uncertainties with the following edits:

- We replaced every mention of “scans” when used with a number to avoid any ambiguity.
- We also added the detail of the MRI and CT slices split (130.3 million MRI and 279.7 million CT) (Methods, Data availability)
- However, we have been very clear in other sections. The Data Availability subsection mentions: 830K patients, 417K MRI studies, 846 CT studies, 3.37 million MRI series, 3.15 million CT series, and 410 million slices (now with 130.3 million MRI and 279.7 million CT). Each hierarchical level was mentioned and detailed. The unified and explicit reporting of counts at each level the reviewer is asking was already provided.

2. The characterization of imaging acquisition remains insufficient for downstream use. While the revision adds some information on scanner vendors and field strengths, the manuscript still lacks structured summaries of acquisition variability (e.g., per-site/per-scanner distributions, sequence-specific counts, voxel size, slice thickness, acquisition planes). Without these details, it is difficult to assess heterogeneity or design harmonization strategies, which limits the practical value of the dataset for neuroimaging research.

answer: Revision

- We have already provided a sequence count for the MRI datareceived by SCANDAN.
- Voxel size/row & columns counts/acquisition plane were not provided as metadata for the whole dataset, and only obtained through the DICOM slices, which for SCANDAN were limited to the 21K MRI studies received. This also limits the sequence count to the SCANDAN data, as it as not yet been carried on the rest of the data.
- Regardless of this, the dataset contains thousands of different acquisition combinations, and such table would be so large that, even if it was authorised to be extracted from the national safe haven, would be difficult to provide in an interpretable format. We understand the request but do not believe it is adapted to a dataset of this size, or from clinical origin.
- We added Table 1 with the amount of unique value for some key metadata: Protocol Name, Series Description, Performed Procedure Step Description, Body Part Examined and Institution Name. We also provided the number of series which had an empty field for these values, which should provide a measure of the heterogeneity of the dataset, as the reviewer explained it was difficult to assess.
- We aim to continue improving the labelling of the scans in future work to provide better characterisation for new users of the data.

3. The quality control strategy is not described at a level expected for large-scale neuroimaging resources. The current approach focuses on rule-based filtering (DICOM tags, NLP exclusions) and manual annotation, but does not include quantitative image quality metrics (e.g., SNR, motion, artifacts) or standardized QC frameworks (e.g., MRIQC-like measures). It is also unclear what proportion of images were excluded due to poor quality versus metadata-based criteria. Without quantitative QC reporting, the usability of the retained dataset remains difficult to evaluate.

answer: No revision When working with images at very large scale, computation is a limiting factor for running standardised QC metrics. We therefore used more computationally efficient methods based on meta-data. We will continue to develop this dataset, and it is an openly available resource for researchers to test these methods in smaller subsets of the data. As we are unable currently to provide the quantitative QC metrics we have not provided these.

4. The manuscript would benefit from a clearer demonstration of the dataset's analytical value. Although this is presented as a Data Note, the current outputs are primarily structural (sequence labeling, report parsing, cohort construction), and there is limited illustration of how the curated data enable downstream scientific or clinical analyses. A more concrete example (even exploratory) of how the dataset can be used for a neuroimaging or clinical research question would strengthen the justification of its utility.

answer: No revisionWe have completed analyses in the data that we willo publish soon, and others are ongoing. The primary purpose of the paper is to present the dataset, and we believe that including analyses would risk lead to a very long paper that would lack focus.

Reviewer 2:

1. The tables are not presented and mentioned in order of appearance (table 3 is mentioned before table 2, table 5 before table 4). Please change to enable a coherent flow of information.

answer: Revision

We edited the numeration of table as well as their order (3>2, 5>3, 2>5) (incremented by one as we added a new table as Table 1.). We also update the Figure numeration (5>1, 1>2, 2>3, 3>5).

2. Table 5 is still unclear to me. From the new legend, the headings of the columns should represent an excluding step, but the fact that it is a brain image or a whole brain image shouldn't be an inclusion step? In other words the 60 series excluded from the "Brain" column, were because they were ore were not containing brain images? I suggest adding a legend or some explanation about what each excluding step means.

answer: Revision

We clarified the legends to emphasise it was indeed exclusion steps. We renamed column which hinted as inclusions step to make clear they were exclusion steps by highlighting what was removed. (Original > Baseline, Brain > Brain Absent, Whole Brain > Partial Brain, Other Body Parts > Other Body Parts Present, Contrast & Angio > Contrast or Angio Present).

Cookie Preference Center

We use cookies which are necessary to make our site work. We may also use additional cookies to analyze, improve and personalize our content and your digital experience. For more information, see our Cookie Policy.

You may choose not to allow some types of cookies. However, blocking some types may impact your experience of our site and the services we are able to offer. See the different category headings below to find out more or change your settings.

Allow all

Manage Consent Preferences

Strictly Necessary Cookies

Always active

These cookies are necessary for the website to function and cannot be switched off in our systems. They are usually only set in response to actions made by you which amount to a request for services, such as setting your privacy preferences, logging in or filling in forms. You can set your browser to block or alert you about these cookies, but some parts of the site will not then work.

Functional Cookies

These cookies enable the website to provide enhanced functionality and personalisation. They may be set by us or by third party providers whose services we have added to our pages. If you do not allow these cookies then some or all of these services may not function properly.

Performance Cookies

These cookies allow us to count visits and traffic sources so we can measure and improve the performance of our site. They help us to know which pages are the most and least popular and see how visitors move around the site.

Targeting Cookies

These cookies may be set through our site by our advertising partners. They may be used by those companies to build a profile of your interests and show you relevant adverts on other sites. If you do not allow these cookies, you will experience less targeted advertising.

Cookie List

Clear

Apply Cancel

Consent Leg.Interest

Confirm my choices

Power
