# Supplementary material for: A large dataset of brain imaging linked to health systems data: curation and access to a whole system national cohort from NHS Scotland
Source: Gigascience. 2026 Jun 9;15:giag072. doi: 10.1093/gigascience/giag072 (PMC13347094; doi:10.1093/gigascience/giag072)
Supplement: giag072_Reviewer_1_Report_original_submission — Reviewer 1 -- 12/14/2025 [file giag072_reviewer_1_report_original_submission.pdf]

Reviewer Report

Title: A large dataset of brain imaging linked to health systems data: curation and access to a whole system national cohort from NHS Scotland

Version: Original Submission **Date:** 12/14/2025

Reviewer name: Reviewer 1

Reviewer Comments to Author:

This Data Note describes the Brain Health Data (BHD) initiative and the SCANDAN-developed curation framework, which assembles a very large, population-scale clinical neuroimaging resource from NHS Scotland (2010-2018). The dataset includes approximately 417,000 MRI scans and 846,000 CT head scans linked to electronic health records (EHRs) and radiology reports, hosted within a Trusted Research Environment (the National Safe Haven) and accessed via PHS eDRIS. Overall, this is a highly valuable national-scale resource of clinically acquired imaging data linked to health records with governed access. The manuscript is generally well written and appropriate for a Data Note; however, it would benefit from clearer quantitative characterization of the dataset and additional technical detail to enhance transparency and reuse.1. The abstract and introduction emphasize the full raw scale of the dataset (hundreds of thousands of MR/CT scans) and the curation framework, whereas the Results section focuses primarily on a derived dementia-matched cohort and a subset of retrieved studies. Please clearly separate these aspects and report, with explicit counts, the total number of raw imaging studies available in SMI (MRI and CT), as well as the number of unique patients.2. Please provide more detailed information on the subset of scans that successfully passed the pipeline filters (e.g., T1/T2/FLAIR sequences, whole-brain coverage, absence of contrast or angiography). Although Table 5 partially addresses this, its framing could be clearerâ€"for example, please clarify whether the "Final" category corresponds to the number of research-ready MRI series per sequence.3. Please clarify what data are currently accessible to external researchers through eDRIS/BHD. For instance, does access include raw DICOM files, NIfTI conversions, derived labels, structured report phenotypes, or predefined example cohorts?4. The manuscript describes rule-based parsing of multiple DICOM tags using regular expressions, together with MRI parameter checks (e.g., TR/TE/TI), to define sequence labels. Please provide a complete list of the regular expression patterns used for sequence, body-part, angiography, and contrast identification, along with information on versioning and how updates were handled across iterative annotation rounds.5. Please clarify how the criterion "3D image volume > 5 litres" is computed and provide a rationale for the choice of this threshold.6. The expert annotation study (n = 713 scans across MRI and CT, with 100 overlapping cases and GLAD adjudication) is a strong component of the work. Please clarify how these 713 scans were sampled (e.g., randomly across sites, scanners, and years; stratified by modality; or enriched for challenging or ambiguous cases).7. The manuscript currently lacks a concise summary of key imaging metadata. For neuroimaging researchers, it would be highly valuable to include aggregate summaries of:(1) scanner vendors/models and magnetic field strengths (for MRI), and their distribution across sites and over time;(2) typical voxel sizes, slice thickness distributions, and acquisition planes for the retained sequences; and(3) rates of partial brain coverage or localizer scans.8.

Please indicate whether defacing is performed or feasible within the Trusted Research Environment for MR T1-weighted volumes, and whether defacing is mandatory for any form of data export (recognizing that raw images may not be exported, but derived images could be generated).9. The manuscript alternates between the terms "scan," "study," "series," and "image." Although these are defined earlier, some later statements could be clarified to avoid ambiguity. For example, the phrase "each study (i.e., one per person)" could be interpreted as implying that each individual has only one study, whereas later sections refer to selecting the "earliest studies associated with these subjects." Please clarify whether "one per person" reflects a deliberate selection criterion or an inherent property of the source data.

Level of Interest

Please indicate how interesting you found the manuscript: Choose an item.

Quality of Written English

Please indicate the quality of language in the manuscript: Choose an item.

Declaration of Competing Interests

Please complete a declaration of competing interests, considering the following questions:

- Have you in the past five years received reimbursements, fees, funding, or salary from an organisation that may in any way gain or lose financially from the publication of this manuscript, either now or in the future?
- Do you hold any stocks or shares in an organisation that may in any way gain or lose financially from the publication of this manuscript, either now or in the future?
- Do you hold or are you currently applying for any patents relating to the content of the manuscript?
- Have you received reimbursements, fees, funding, or salary from an organization that holds or has applied for patents relating to the content of the manuscript?
- Do you have any other financial competing interests?
- Do you have any non-financial competing interests in relation to this paper?

If you can answer no to all of the above, write 'I declare that I have no competing interests' below. If your reply is yes to any, please give details below.

I declare that I have no competing interests

I agree to the open peer review policy of the journal. I understand that my name will be included on my report to the authors and, if the manuscript is accepted for publication, my named report including any attachments I upload will be posted on the website along with the authors' responses. I agree for my report to be made available under an Open Access Creative Commons CC-BY license

(http://creativecommons.org/licenses/by/4.0/). I understand that any comments which I do not wish to be included in my named report can be included as confidential comments to the editors, which will not be published.

Choose an item.

To further support our reviewers, we have joined with Publons, where you can gain additional credit to further highlight your hard work (see: https://publons.com/journal/530/gigascience). On publication of this paper, your review will be automatically added to Publons, you can then choose whether or not to claim your Publons credit. I understand this statement.

Yes Choose an item.
