# Supplementary material for: A large dataset of brain imaging linked to health systems data: curation and access to a whole system national cohort from NHS Scotland
Source: Gigascience. 2026 Jun 9;15:giag072. doi: 10.1093/gigascience/giag072 (PMC13347094; doi:10.1093/gigascience/giag072)
Supplement: giag072_Reviewer_1_Report_revision_1 — Reviewer 1 -- 4/13/2026 [file giag072_reviewer_1_report_revision_1.pdf]

Reviewer Report

Title: A large dataset of brain imaging linked to health systems data: curation and access to a whole system national cohort from NHS Scotland

Version: Revision 1 **Date:** 4/13/2026

Reviewer name: Reviewer 1

Reviewer Comments to Author:

The revised manuscript has partially addressed some of the previous concerns; however, several important major issues still remain unclear:1. The description of dataset scale still remains largely inconsistent and potentially confusing. The manuscript reports quantities at different hierarchical levels (e.g., "417,000 MRI", "3.37 million series", "409 million slices"), but does not consistently clarify whether counts refer to studies, series, slices, or unique individuals. For example, it is not always clear whether "417,000 MRI" refers to studies or patients, which represents fundamentally different dataset scales. A unified and explicit reporting of counts at each level (patients, studies, series, slices) is still needed to make the dataset interpretable and comparable.2. The characterization of imaging acquisition remains insufficient for downstream use. While the revision adds some information on scanner vendors and field strengths, the manuscript still lacks structured summaries of acquisition variability (e.g., per-site/per-scanner distributions, sequence-specific counts, voxel size, slice thickness, acquisition planes). Without these details, it is difficult to assess heterogeneity or design harmonization strategies, which limits the practical value of the dataset for neuroimaging research.3. The quality control strategy is not described at a level expected for large-scale neuroimaging resources. The current approach focuses on rule-based filtering (DICOM tags, NLP exclusions) and manual annotation, but does not include quantitative image quality metrics (e.g., SNR, motion, artifacts) or standardized QC frameworks (e.g., MRIQC-like measures). It is also unclear what proportion of images were excluded due to poor quality versus metadata-based criteria. Without quantitative QC reporting, the usability of the retained dataset remains difficult to evaluate.4. The manuscript would benefit from a clearer demonstration of the dataset's analytical value. Although this is presented as a Data Note, the current outputs are primarily structural (sequence labeling, report parsing, cohort construction), and there is limited illustration of how the curated data enable downstream scientific or clinical analyses. A more concrete example (even exploratory) of how the dataset can be used for a neuroimaging or clinical research question would strengthen the justification of its utility.

Level of Interest

Please indicate how interesting you found the manuscript: Choose an item.

Quality of Written English

Please indicate the quality of language in the manuscript: Choose an item.

Declaration of Competing Interests

Please complete a declaration of competing interests, considering the following questions:

- Have you in the past five years received reimbursements, fees, funding, or salary from an organisation that may in any way gain or lose financially from the publication of this manuscript, either now or in the future?
- Do you hold any stocks or shares in an organisation that may in any way gain or lose financially from the publication of this manuscript, either now or in the future?
- Do you hold or are you currently applying for any patents relating to the content of the manuscript?
- Have you received reimbursements, fees, funding, or salary from an organization that holds or has applied for patents relating to the content of the manuscript?
- Do you have any other financial competing interests?
- Do you have any non-financial competing interests in relation to this paper?

If you can answer no to all of the above, write 'I declare that I have no competing interests' below. If your reply is yes to any, please give details below.

I declare that I have no competing interests

I agree to the open peer review policy of the journal. I understand that my name will be included on my report to the authors and, if the manuscript is accepted for publication, my named report including any attachments I upload will be posted on the website along with the authors' responses. I agree for my report to be made available under an Open Access Creative Commons CC-BY license (http://creativecommons.org/licenses/by/4.0/). I understand that any comments which I do not wish to be included in my named report can be included as confidential comments to the editors, which will not be published.

I agree to the open peer review policy of the journal

To further support our reviewers, we have joined with Publons, where you can gain additional credit to further highlight your hard work (see: https://publons.com/journal/530/gigascience). On publication of this paper, your review will be automatically added to Publons, you can then choose whether or not to claim your Publons credit. I understand this statement.

Yes Choose an item.
