# Supplementary material for: A large dataset of brain imaging linked to health systems data: curation and access to a whole system national cohort from NHS Scotland
Source: Gigascience. 2026 Jun 9;15:giag072. doi: 10.1093/gigascience/giag072 (PMC13347094; doi:10.1093/gigascience/giag072)
Supplement: giag072_Reviewer_2_Report_original_submission — Reviewer 2 -- 12/21/2025 [file giag072_reviewer_2_report_original_submission.pdf]

Reviewer Report

Title: A large dataset of brain imaging linked to health systems data: curation and access to a whole system national cohort from NHS Scotland

Version: Original Submission **Date:** 12/21/2025

Reviewer name: Reviewer 2

Reviewer Comments to Author:

This Data Note describes the design, governance, and technical curation of the Brain Health Data (BHD) initiative, a large-scale, population-wide brain imaging dataset from NHS Scotland that links MRI and CT scans with longitudinal electronic health records to support dementia and neurodegenerative disease research.The paper's main strengths lie in the scale and ambition of the effort, which brings together multiple working groups to curate and operationalise a nationwide clinical brain imaging resource that reflects real-world clinical practice. The authors clearly recognise the importance of making such a resource available to the wider research community, and the emphasis on governed but streamlined access underscores a genuine commitment to data sharing and reuse. Particularly commendable is the open and honest discussion of the practical, technical, and governance challenges encountered in building and maintaining the dataset, which provides valuable insight for others undertaking similar work and enhances the potential impact of this resource for the dementia research community.Overall, the ambition and scope are clear, but the presentation sometimes obscures the main narrative: the sequencing of methods, validation steps, and resulting datasets can be difficult to follow, and readers may struggle to form a clear mental model of how individual processing steps connect to the final cohorts and tables. Improved signposting and tighter alignment between described processes, figures, and reported results would substantially enhance clarity.My detailed comments are as follows:* Several figures aim to provide an overview of the data curation and processing pipeline, but the relationship between the overall SCANDAN project, the specific scope of this Data Note, and the individual curation steps described in the Methods is not always clear. In particular, Figure 1 illustrates the work packages of the overall SCANDAN project; however, it is not immediately clear how these relate to the specific focus of this manuscript. While SCANDAN aims to develop and evaluate dementia classifiers, this paper appears to focus primarily on the data curation and preparation framework. I encourage the authors to clarify in the text and figure legend which components of Figure 1 are directly covered in this manuscript, and which are shown for broader project context.* Multiple data selection and refinement steps are described across the manuscript, including DICOM-based filtering, NLP-driven exclusions, manual annotation, and iterative re-annotation, but their sequencing and respective roles can be difficult to follow. Figure 4 appears to attempt to bring these elements together, yet it is not explicitly referenced in the text, and its legend lacks sufficient explanation to map the visual elements (including the numbered markers) to the corresponding methodological steps. More explicit cross-referencing between text and figures, and a concise summary (e.g. a short paragraph, table, or expanded figure legend) outlining the full sequence of automated and manual steps, would help clarify how each contributes to the final curated dataset.* Data sources section: the description of data linkage and

processing (e.g. "We linked each study…", "All data was processed and stored…") would benefit from clarification on which steps are automated and which require manual intervention.* NLP tool: it would be useful to clarify whether the NLP tool is openly available (e.g. as code or a container), as this could be of significant value to the community. In addition, the sentence on page 6 describing the "enhancement of the detection of section boundaries" is highly technical and difficult to follow in this context; a brief rephrasing or explanation of what is meant by section boundaries would help. More generally, a short high-level description of the NLP inputs, outputs, and how these outputs are used within the broader framework would improve readability.* Phenotyping dementia: this section appears to be missing a reference to Table 1. In addition, Table 1 would benefit from a more informative legend explaining how it should be interpreted. For example, it is unclear why a BNF code is reported only for Alzheimer's disease. While the use of wildcards is understandable, the rationale and intended use of these wildcards should be explicitly stated in the table legend.* Cohort building: the description of cohort selection (e.g. "We first selected… gone through the pipeline… and had no NLP label") would benefit from clearer specification of the order of steps, and of which steps are automated versus manual. Explicit input/output descriptions and a reference to a visual flowchart would help clarify this process.* DICOM tags: similarly, the description of how DICOM-derived labels are combined with NLP outputs would benefit from clearer sequencing and explicit reference to a flowchart illustrating the full pipeline.* Data validation and quality control: Table 2 appears to relate to this section but is only explicitly referenced in the Results section. It is also unclear what components are included in the "automated labelling process described above" (e.g. DICOM-based rules only, NLP-derived information, or both). Clarification would also be helpful on what constitutes the "ground truth" used for this comparison, in particular whether the results in Table 2 reflect the final labels after all rounds of annotation, GLAD aggregation, and expert adjudication.* Use of GLAD: the role of GLAD in the workflow would benefit from further clarification. Specifically, it would be helpful to briefly state what inputs are provided to GLAD, what outputs are produced, and how these outputs are used in the evaluation of the automated labelling. It is currently unclear whether the results in Table 2 are based on GLAD-inferred labels or on subsequent manually adjudicated labels.* Results of data validation and quality control: again, clarification on what is included under "automated tools" would help ensure consistency with the workflow described in the Methods. The authors might also consider illustrating the iterative annotation and adjudication process in a dedicated flowchart.* Discussion: the authors note that the dataset will continue to expand in size; a brief summary of future plans for cohort growth and linkage would be of interest.Minor points:* Phenotyping dementia - a reference appears to be missing.* Figure 3 legend: consider expanding it to include the explanation of colours and acronyms currently provided only in the text.* Page 11: "sizer" appears to be a typo and should read "size".

Level of Interest

Please indicate how interesting you found the manuscript: Choose an item.

Quality of Written English

Please indicate the quality of language in the manuscript: Choose an item.

Declaration of Competing Interests

Please complete a declaration of competing interests, considering the following questions:

- Have you in the past five years received reimbursements, fees, funding, or salary from an organisation that may in any way gain or lose financially from the publication of this manuscript, either now or in the future?
- Do you hold any stocks or shares in an organisation that may in any way gain or lose financially from the publication of this manuscript, either now or in the future?
- Do you hold or are you currently applying for any patents relating to the content of the manuscript?
- Have you received reimbursements, fees, funding, or salary from an organization that holds or has applied for patents relating to the content of the manuscript?
- Do you have any other financial competing interests?
- Do you have any non-financial competing interests in relation to this paper?

If you can answer no to all of the above, write 'I declare that I have no competing interests' below. If your reply is yes to any, please give details below.

I declare that I have no competing interests

I agree to the open peer review policy of the journal. I understand that my name will be included on my report to the authors and, if the manuscript is accepted for publication, my named report including any attachments I upload will be posted on the website along with the authors' responses. I agree for my report to be made available under an Open Access Creative Commons CC-BY license (http://creativecommons.org/licenses/by/4.0/). I understand that any comments which I do not wish to be included in my named report can be included as confidential comments to the editors, which will not be published.

Choose an item.

To further support our reviewers, we have joined with Publons, where you can gain additional credit to further highlight your hard work (see: https://publons.com/journal/530/gigascience). On publication of this paper, your review will be automatically added to Publons, you can then choose whether or not to claim your Publons credit. I understand this statement.

Yes Choose an item.
